# Supplementary material for: A Novel Team-Based Learning Approach for an Internal Medicine Residency: Medication-Assisted Treatments for Substance Use Disorders
Source: MedEdPORTAL. 2021 Feb 1;17:11085. doi: 10.15766/mep_2374-8265.11085 (PMC7852341; doi:10.15766/mep_2374-8265.11085)
Supplement: Supplementary file 1 — iRAT without Answers.docxiRAT with Answers.docxTeam Application Exercise.pptxFacilitators Guide to the Team App Exercise.docxResident Evaluation of the TBL Activity.docx [file mep_2374-8265.11085-s001.zip › A. iRAT without Answers.docx]

**Substance Use Disorder TBL iRAT**

1. Fill in the following chart regarding screening for substance use disorders:

| Substance | Who should get screened? | What tool(s) can you use? |
| --- | --- | --- |
| Tobacco |  |  |
| Alcohol |  |  |
| Illicit drugs |  |  |

1. What are some non-pharmacologic AND pharmacologic treatments for alcohol use disorder?
2. What are some contraindications for the below medications?

Naltrexone:

Acamprosate:

Disulfiram:

1. What is the mechanism of action of buprenorphine/naloxone?
