# Supplementary material for: A Novel Team-Based Learning Approach for an Internal Medicine Residency: Medication-Assisted Treatments for Substance Use Disorders
Source: MedEdPORTAL. 2021 Feb 1;17:11085. doi: 10.15766/mep_2374-8265.11085 (PMC7852341; doi:10.15766/mep_2374-8265.11085)
Supplement: Supplementary file 1 — iRAT without Answers.docxiRAT with Answers.docxTeam Application Exercise.pptxFacilitators Guide to the Team App Exercise.docxResident Evaluation of the TBL Activity.docx [file mep_2374-8265.11085-s001.zip › B. iRAT with Answers.docx]

**ATTENTION, STUDENTS:** If you are accessing this material BEFORE it is used in your course, please do NOT read this document prior to the class session. An answer key is included in this module, which is designed to lead you through a learning experience that reinforces your knowledge of the content. Early review or dissemination of this material to others will diminish the learning opportunity and be considered academic misconduct.

**Substance Use Disorder TBL iRAT**

1. Fill in the following chart regarding screening for substance use disorders:

| Substance | Who should get screened? | What tool(s) can you use? |
| --- | --- | --- |
| Tobacco | Every adult | 5As (Ask, Advise, Assess, Assist, Arrange) |
| Alcohol | Every adult | AUDIT (Alcohol Use Disorders identification test), AUDIT-C (Three items from AUDIT), single-item screening |
| Illicit drugs | No clear guidelines, maybe high risk? | DAST (Drug Abuse Screening Test): similar to AUDIT but for illicit drugs |

1. What are some non-pharmacologic AND pharmacologic treatments for alcohol use disorder?

The primary treatment of alcohol use disorder is psychosocial interventions (counseling, motivational interviewing, CBT, residential care, peer support groups such as Alcoholics Anonymous). Pharmacotherapy (naltrexone, disulfiram, gabapentin, topiramate, and acamprosate) can be used in combination with psychosocial treatment.

1. What are some contraindications for the below medications?

Naltrexone: contraindicated in patients receiving or withdrawing from any opioid and in those with liver failure (acute) or hepatitis, not studied in cirrhotics and not currently recommended

Acamprosate: current EtOH use (ideally patient should stop drinking before starting acamprosate, making it less than ideal), contraindicated in advanced kidney disease

Disulfiram: current EtOH use, cirrhosis, coronary artery disease, severe myocardial disease

4. What is the mechanism of action of buprenorphine/naloxone?

Buprenorphine: Partial opioid agonist. Strong affinity to µ-opioid receptor. It provides the patient with milder effects through the opioid receptor (providing some analgesia) but has lower abuse potential and lower dependency. At higher doses buprenorphine’s agonist effects can plateau and it can start to act as an antagonist. It has better oral bioavailability than naloxone

Naloxone: Pure Opioid antagonist (highest for µ-opioid receptor). However, it has poor oral bioavailability.

When the combination drug is taken orally, buprenorphine predominates. However, if the patient attempts to inject (IV or IM), naloxone takes predominant effect as an antagonist, either blocking the euphoric effects of buprenorphine or even precipitating withdrawal symptoms in those dependent on other opioids.
