# Supplementary material for: A Novel Team-Based Learning Approach for an Internal Medicine Residency: Medication-Assisted Treatments for Substance Use Disorders
Source: MedEdPORTAL. 2021 Feb 1;17:11085. doi: 10.15766/mep_2374-8265.11085 (PMC7852341; doi:10.15766/mep_2374-8265.11085)
Supplement: Supplementary file 1 — iRAT without Answers.docxiRAT with Answers.docxTeam Application Exercise.pptxFacilitators Guide to the Team App Exercise.docxResident Evaluation of the TBL Activity.docx [file mep_2374-8265.11085-s001.zip › C. Team Application Exercise.pptx]

## Slide 1
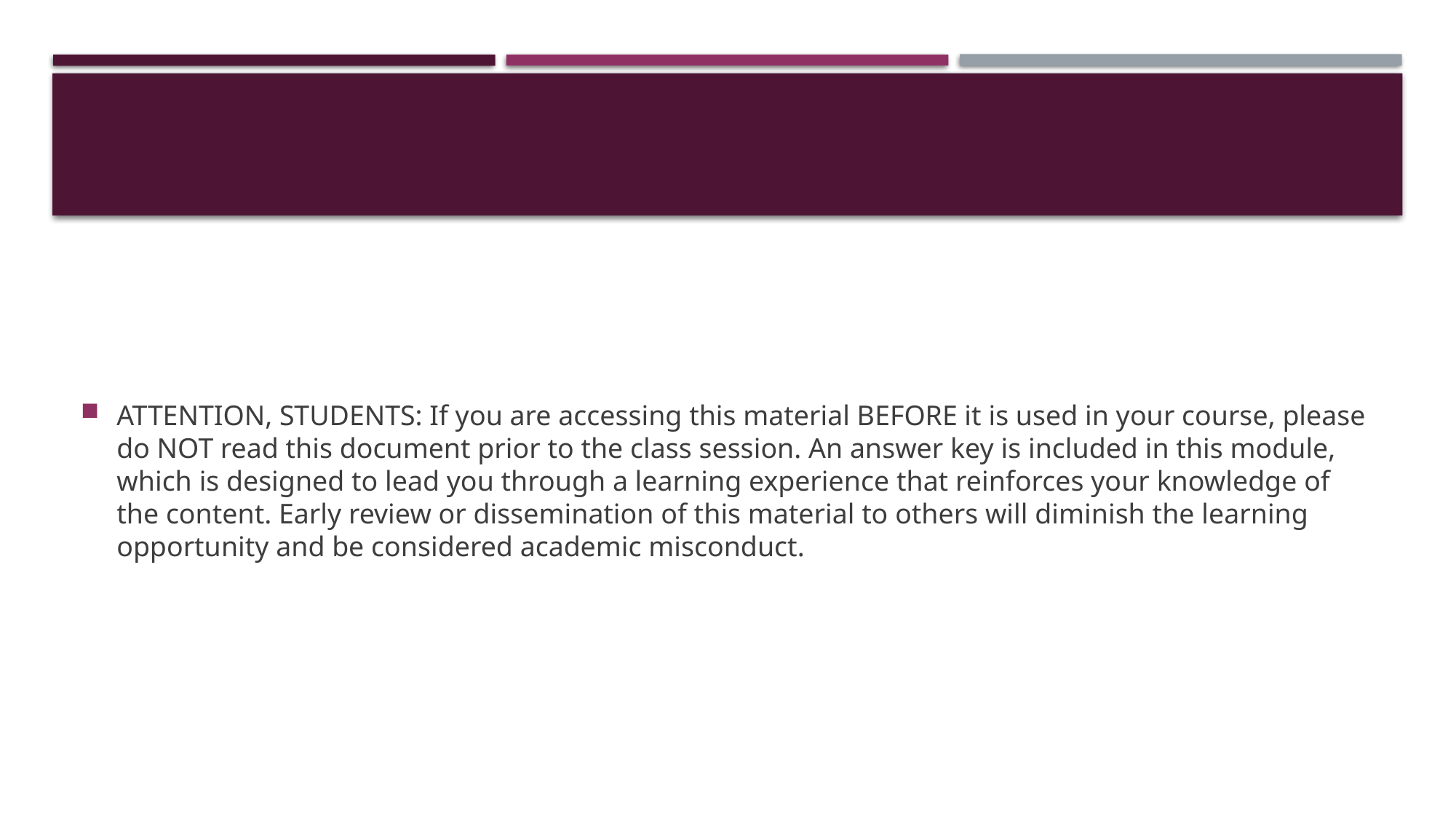

#
ATTENTION, STUDENTS: If you are accessing this material BEFORE it is used in your course, please do NOT read this document prior to the class session. An answer key is included in this module, which is designed to lead you through a learning experience that reinforces your knowledge of the content. Early review or dissemination of this material to others will diminish the learning opportunity and be considered academic misconduct.

## Slide 2
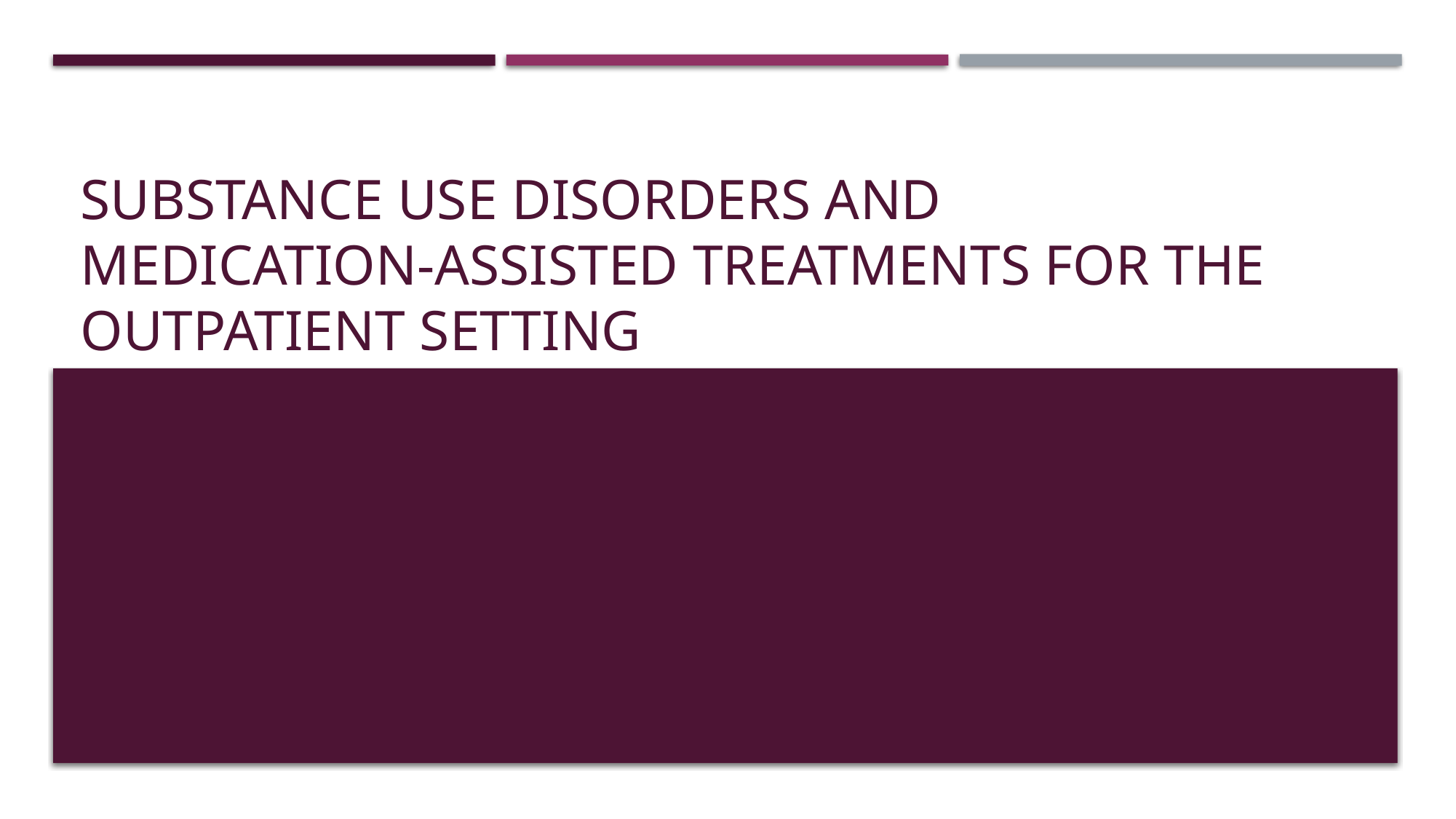

# Substance use Disorders and medication-assisted treatments For the Outpatient Setting

## Slide 3
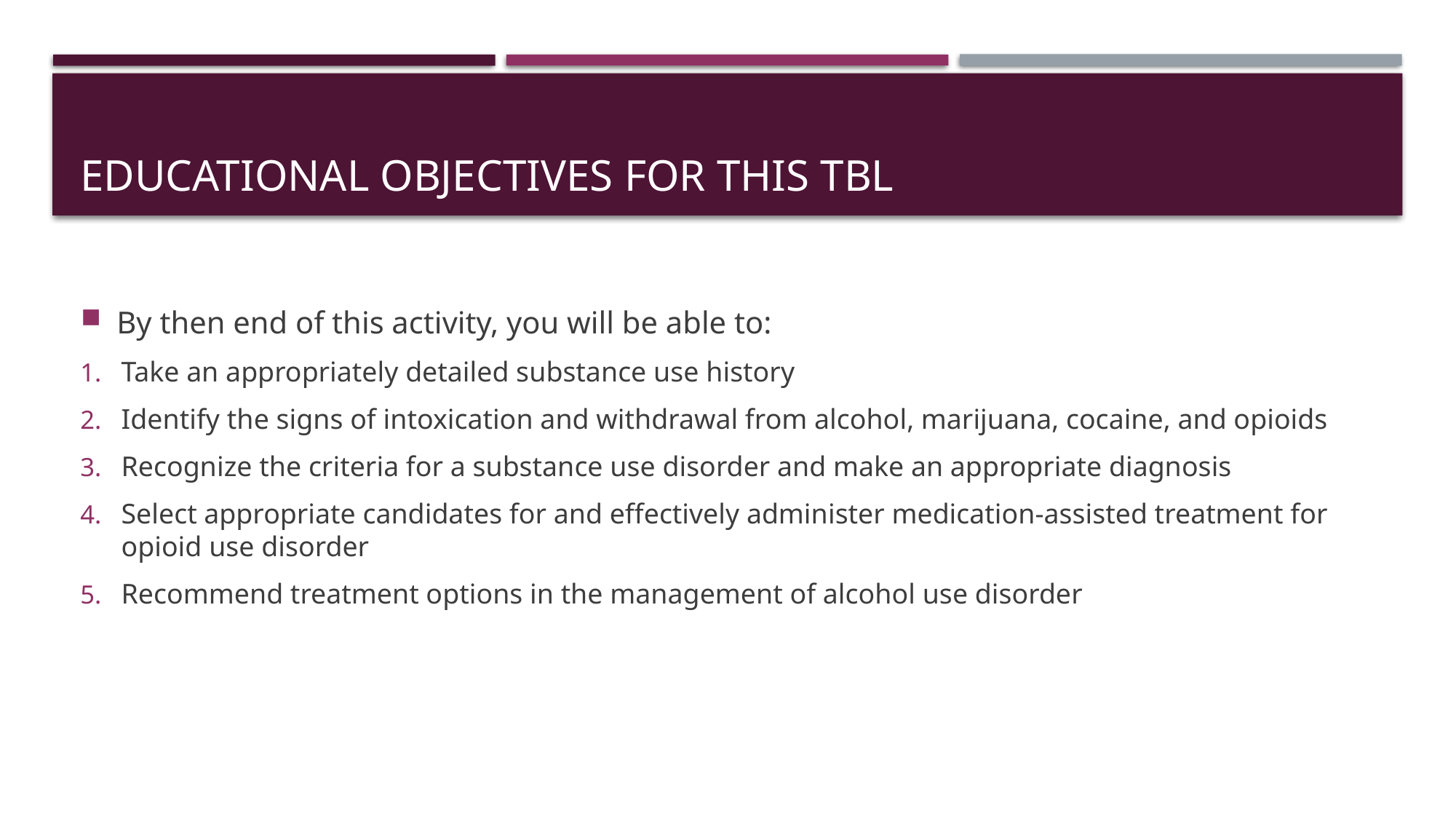

# Educational objectives for this tbl
By then end of this activity, you will be able to:
Take an appropriately detailed substance use history
Identify the signs of intoxication and withdrawal from alcohol, marijuana, cocaine, and opioids
Recognize the criteria for a substance use disorder and make an appropriate diagnosis
Select appropriate candidates for and effectively administer medication-assisted treatment for opioid use disorder
Recommend treatment options in the management of alcohol use disorder

## Slide 4
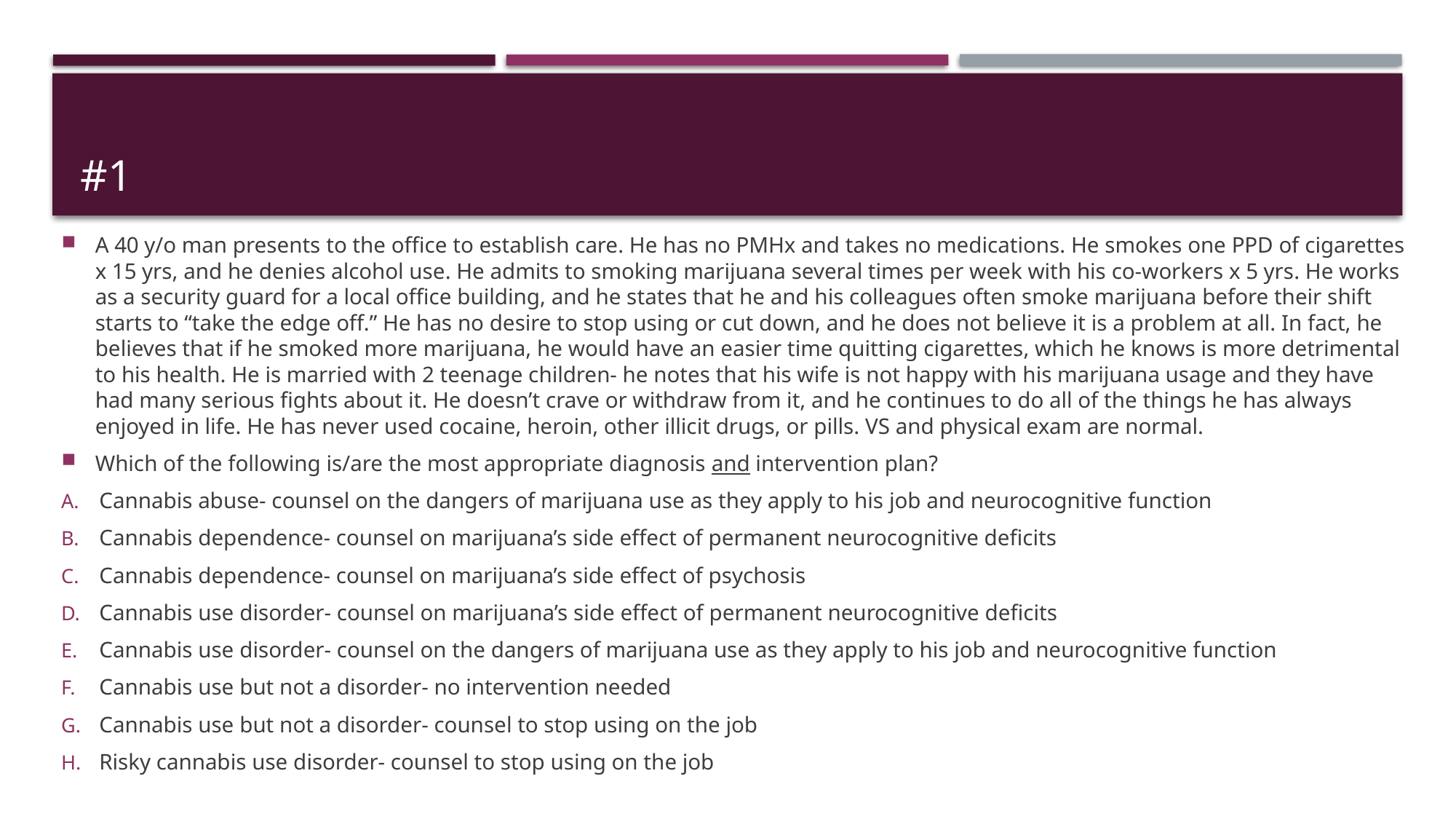

# #1
A 40 y/o man presents to the office to establish care. He has no PMHx and takes no medications. He smokes one PPD of cigarettes x 15 yrs, and he denies alcohol use. He admits to smoking marijuana several times per week with his co-workers x 5 yrs. He works as a security guard for a local office building, and he states that he and his colleagues often smoke marijuana before their shift starts to “take the edge off.” He has no desire to stop using or cut down, and he does not believe it is a problem at all. In fact, he believes that if he smoked more marijuana, he would have an easier time quitting cigarettes, which he knows is more detrimental to his health. He is married with 2 teenage children- he notes that his wife is not happy with his marijuana usage and they have had many serious fights about it. He doesn’t crave or withdraw from it, and he continues to do all of the things he has always enjoyed in life. He has never used cocaine, heroin, other illicit drugs, or pills. VS and physical exam are normal.
Which of the following is/are the most appropriate diagnosis and intervention plan?
Cannabis abuse- counsel on the dangers of marijuana use as they apply to his job and neurocognitive function
Cannabis dependence- counsel on marijuana’s side effect of permanent neurocognitive deficits
Cannabis dependence- counsel on marijuana’s side effect of psychosis
Cannabis use disorder- counsel on marijuana’s side effect of permanent neurocognitive deficits
Cannabis use disorder- counsel on the dangers of marijuana use as they apply to his job and neurocognitive function
Cannabis use but not a disorder- no intervention needed
Cannabis use but not a disorder- counsel to stop using on the job
Risky cannabis use disorder- counsel to stop using on the job

## Slide 5
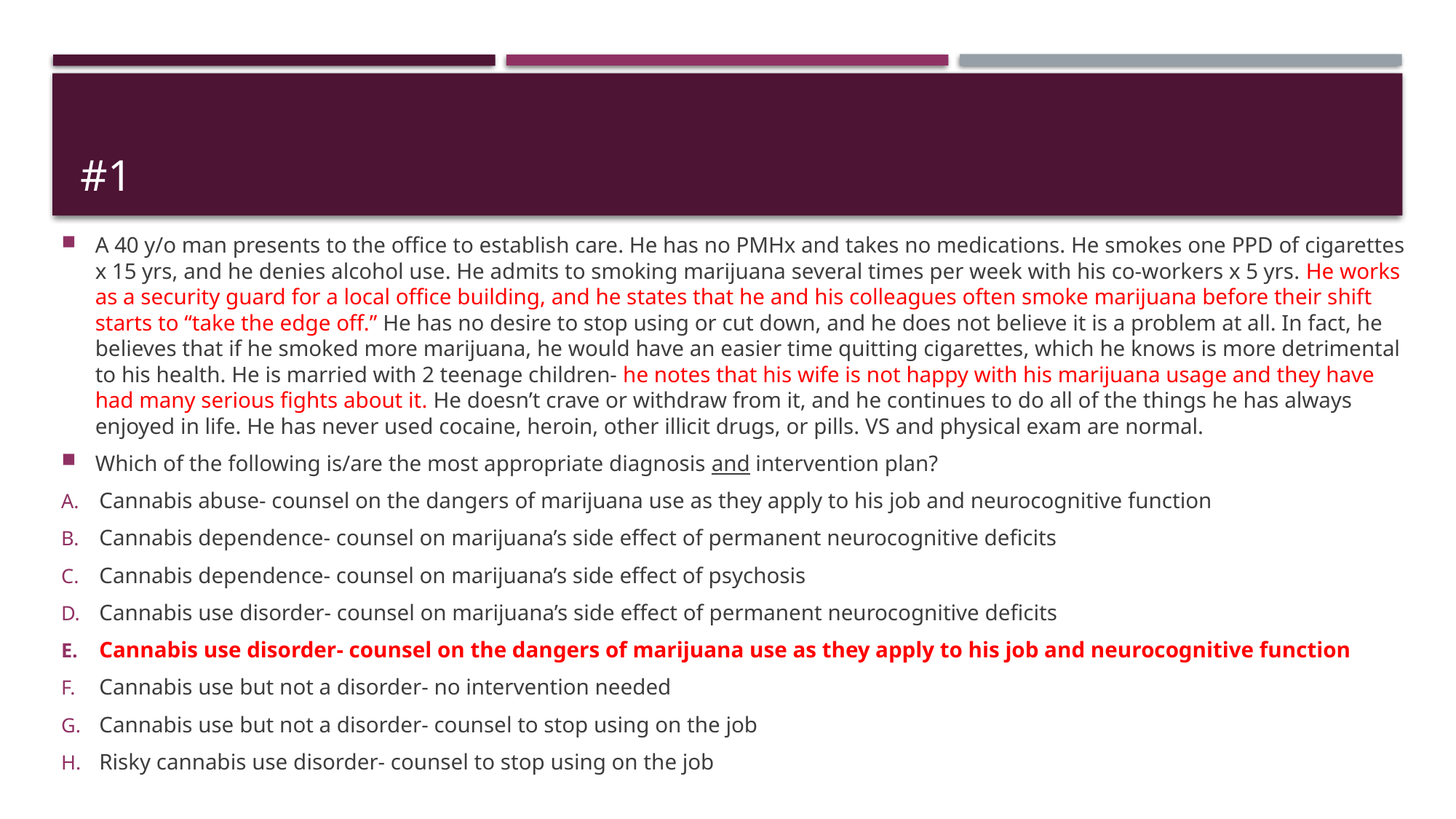

# #1
A 40 y/o man presents to the office to establish care. He has no PMHx and takes no medications. He smokes one PPD of cigarettes x 15 yrs, and he denies alcohol use. He admits to smoking marijuana several times per week with his co-workers x 5 yrs. He works as a security guard for a local office building, and he states that he and his colleagues often smoke marijuana before their shift starts to “take the edge off.” He has no desire to stop using or cut down, and he does not believe it is a problem at all. In fact, he believes that if he smoked more marijuana, he would have an easier time quitting cigarettes, which he knows is more detrimental to his health. He is married with 2 teenage children- he notes that his wife is not happy with his marijuana usage and they have had many serious fights about it. He doesn’t crave or withdraw from it, and he continues to do all of the things he has always enjoyed in life. He has never used cocaine, heroin, other illicit drugs, or pills. VS and physical exam are normal.
Which of the following is/are the most appropriate diagnosis and intervention plan?
Cannabis abuse- counsel on the dangers of marijuana use as they apply to his job and neurocognitive function
Cannabis dependence- counsel on marijuana’s side effect of permanent neurocognitive deficits
Cannabis dependence- counsel on marijuana’s side effect of psychosis
Cannabis use disorder- counsel on marijuana’s side effect of permanent neurocognitive deficits
Cannabis use disorder- counsel on the dangers of marijuana use as they apply to his job and neurocognitive function
Cannabis use but not a disorder- no intervention needed
Cannabis use but not a disorder- counsel to stop using on the job
Risky cannabis use disorder- counsel to stop using on the job

## Slide 6
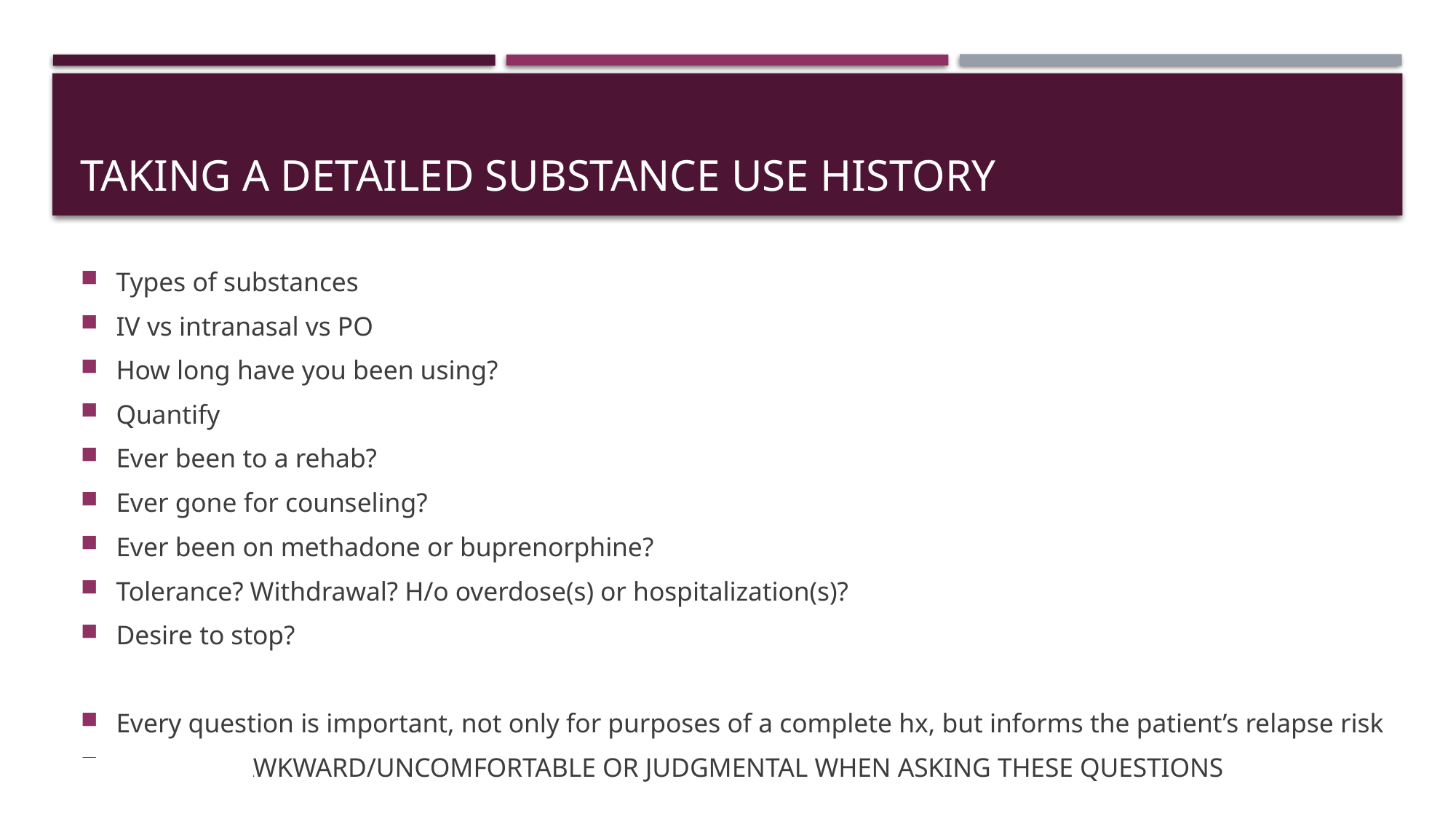

# Taking a detailed substance use history
Types of substances
IV vs intranasal vs PO
How long have you been using?
Quantify
Ever been to a rehab?
Ever gone for counseling?
Ever been on methadone or buprenorphine?
Tolerance? Withdrawal? H/o overdose(s) or hospitalization(s)?
Desire to stop?
Every question is important, not only for purposes of a complete hx, but informs the patient’s relapse risk
DON’T BE AWKWARD/UNCOMFORTABLE OR JUDGMENTAL WHEN ASKING THESE QUESTIONS

## Slide 7
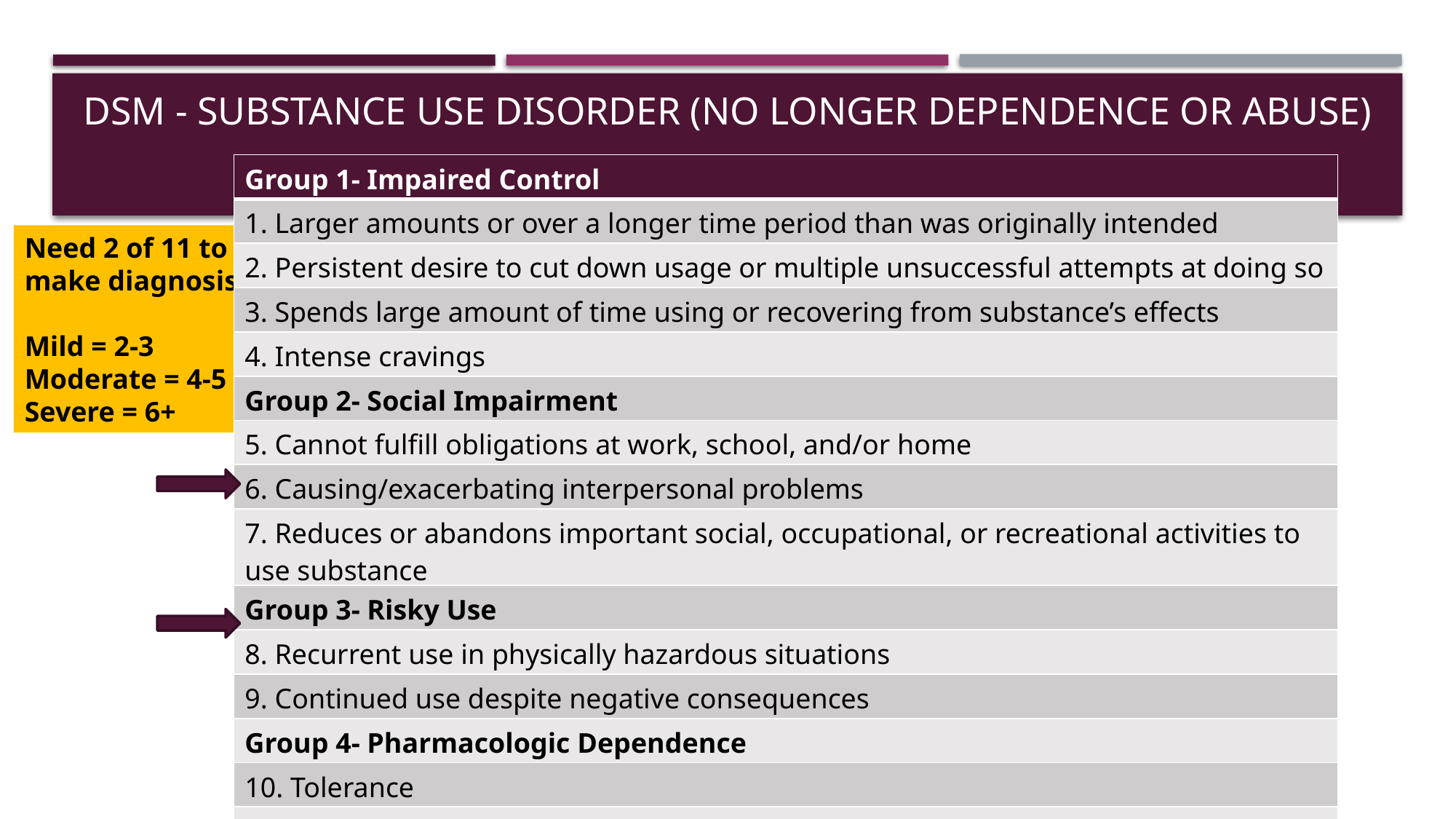

# DSM - Substance Use Disorder (no longer dependence or abuse)
| Group 1- Impaired Control |
| --- |
| 1. Larger amounts or over a longer time period than was originally intended |
| 2. Persistent desire to cut down usage or multiple unsuccessful attempts at doing so |
| 3. Spends large amount of time using or recovering from substance’s effects |
| 4. Intense cravings |
| Group 2- Social Impairment |
| 5. Cannot fulfill obligations at work, school, and/or home |
| 6. Causing/exacerbating interpersonal problems |
| 7. Reduces or abandons important social, occupational, or recreational activities to use substance |
| Group 3- Risky Use |
| 8. Recurrent use in physically hazardous situations |
| 9. Continued use despite negative consequences |
| Group 4- Pharmacologic Dependence |
| 10. Tolerance |
| 11. Withdrawal |
Need 2 of 11 to make diagnosis
Mild = 2-3
Moderate = 4-5
Severe = 6+

## Slide 8
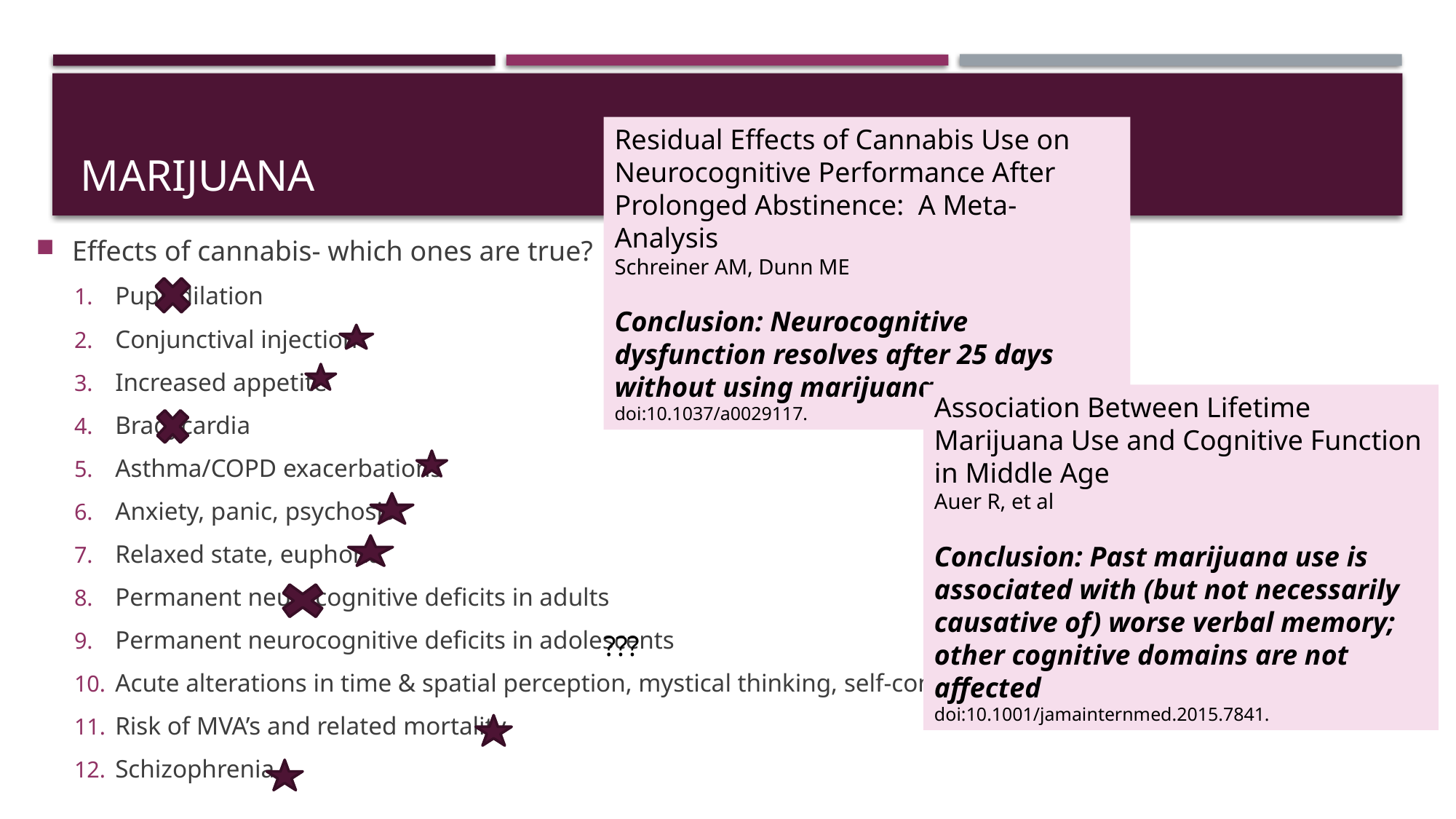

# Marijuana
Residual Effects of Cannabis Use on Neurocognitive Performance After Prolonged Abstinence: A Meta-Analysis
Schreiner AM, Dunn ME
Conclusion: Neurocognitive dysfunction resolves after 25 days without using marijuana
doi:10.1037/a0029117.
Effects of cannabis- which ones are true?
Pupil dilation
Conjunctival injection
Increased appetite
Bradycardia
Asthma/COPD exacerbations
Anxiety, panic, psychosis
Relaxed state, euphoria
Permanent neurocognitive deficits in adults
Permanent neurocognitive deficits in adolescents
Acute alterations in time & spatial perception, mystical thinking, self-consciousness
Risk of MVA’s and related mortality
Schizophrenia
Association Between Lifetime Marijuana Use and Cognitive Function in Middle Age
Auer R, et al
Conclusion: Past marijuana use is associated with (but not necessarily causative of) worse verbal memory; other cognitive domains are not affected
doi:10.1001/jamainternmed.2015.7841.
???

## Slide 9
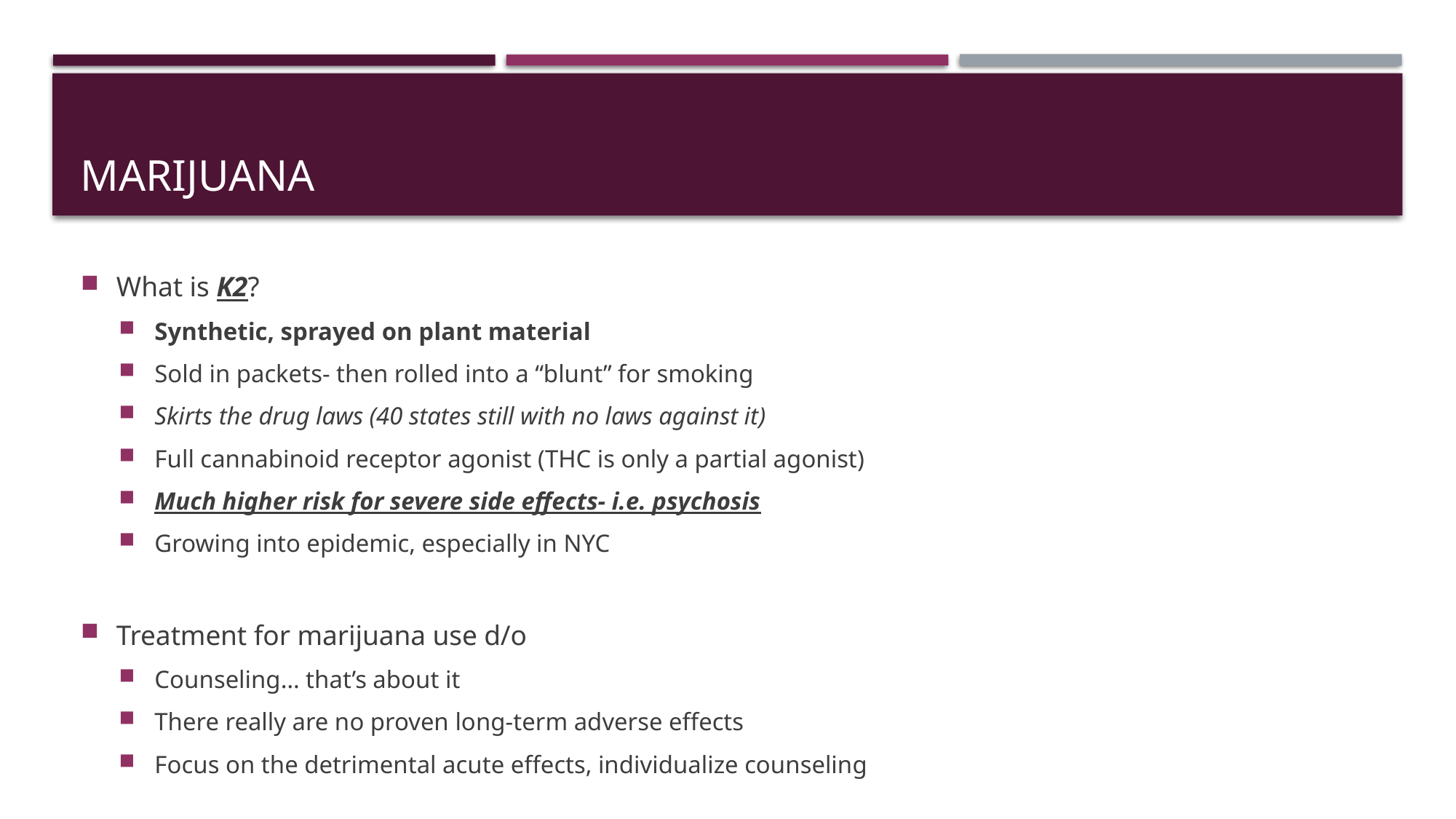

# Marijuana
What is K2?
Synthetic, sprayed on plant material
Sold in packets- then rolled into a “blunt” for smoking
Skirts the drug laws (40 states still with no laws against it)
Full cannabinoid receptor agonist (THC is only a partial agonist)
Much higher risk for severe side effects- i.e. psychosis
Growing into epidemic, especially in NYC
Treatment for marijuana use d/o
Counseling… that’s about it
There really are no proven long-term adverse effects
Focus on the detrimental acute effects, individualize counseling

## Slide 10
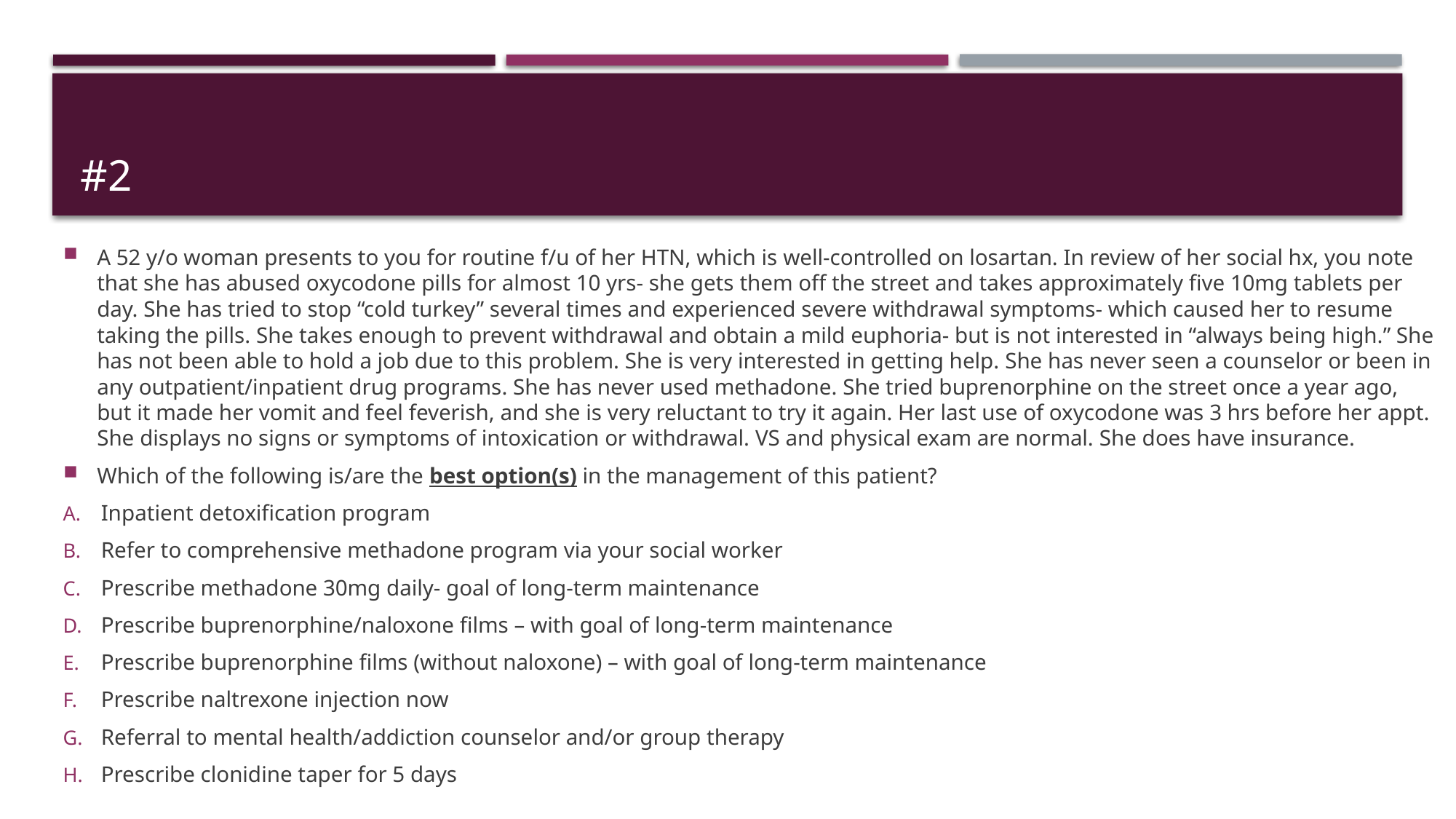

# #2
A 52 y/o woman presents to you for routine f/u of her HTN, which is well-controlled on losartan. In review of her social hx, you note that she has abused oxycodone pills for almost 10 yrs- she gets them off the street and takes approximately five 10mg tablets per day. She has tried to stop “cold turkey” several times and experienced severe withdrawal symptoms- which caused her to resume taking the pills. She takes enough to prevent withdrawal and obtain a mild euphoria- but is not interested in “always being high.” She has not been able to hold a job due to this problem. She is very interested in getting help. She has never seen a counselor or been in any outpatient/inpatient drug programs. She has never used methadone. She tried buprenorphine on the street once a year ago, but it made her vomit and feel feverish, and she is very reluctant to try it again. Her last use of oxycodone was 3 hrs before her appt. She displays no signs or symptoms of intoxication or withdrawal. VS and physical exam are normal. She does have insurance.
Which of the following is/are the best option(s) in the management of this patient?
Inpatient detoxification program
Refer to comprehensive methadone program via your social worker
Prescribe methadone 30mg daily- goal of long-term maintenance
Prescribe buprenorphine/naloxone films – with goal of long-term maintenance
Prescribe buprenorphine films (without naloxone) – with goal of long-term maintenance
Prescribe naltrexone injection now
Referral to mental health/addiction counselor and/or group therapy
Prescribe clonidine taper for 5 days

## Slide 11
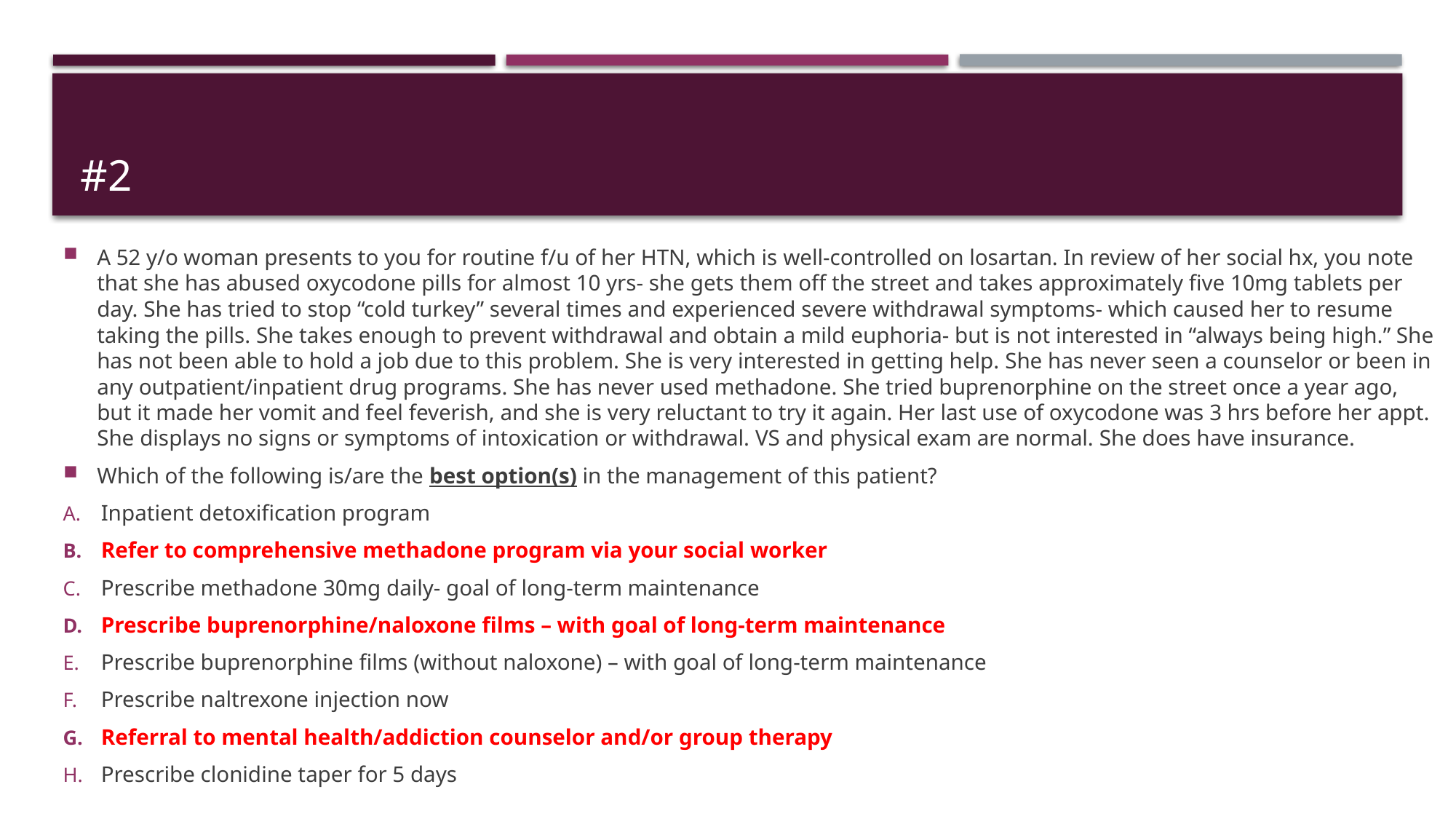

# #2
A 52 y/o woman presents to you for routine f/u of her HTN, which is well-controlled on losartan. In review of her social hx, you note that she has abused oxycodone pills for almost 10 yrs- she gets them off the street and takes approximately five 10mg tablets per day. She has tried to stop “cold turkey” several times and experienced severe withdrawal symptoms- which caused her to resume taking the pills. She takes enough to prevent withdrawal and obtain a mild euphoria- but is not interested in “always being high.” She has not been able to hold a job due to this problem. She is very interested in getting help. She has never seen a counselor or been in any outpatient/inpatient drug programs. She has never used methadone. She tried buprenorphine on the street once a year ago, but it made her vomit and feel feverish, and she is very reluctant to try it again. Her last use of oxycodone was 3 hrs before her appt. She displays no signs or symptoms of intoxication or withdrawal. VS and physical exam are normal. She does have insurance.
Which of the following is/are the best option(s) in the management of this patient?
Inpatient detoxification program
Refer to comprehensive methadone program via your social worker
Prescribe methadone 30mg daily- goal of long-term maintenance
Prescribe buprenorphine/naloxone films – with goal of long-term maintenance
Prescribe buprenorphine films (without naloxone) – with goal of long-term maintenance
Prescribe naltrexone injection now
Referral to mental health/addiction counselor and/or group therapy
Prescribe clonidine taper for 5 days

## Slide 12
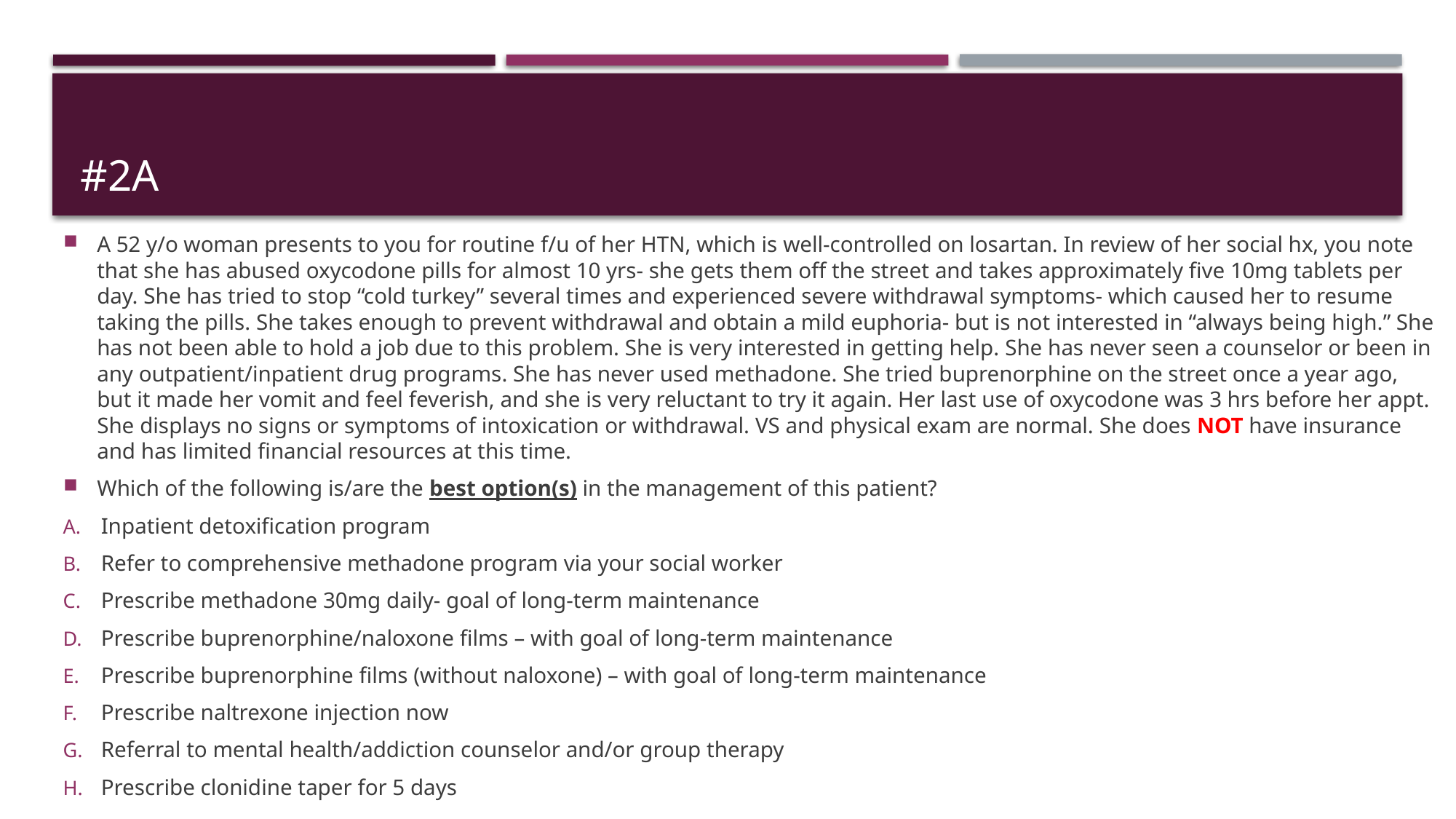

# #2A
A 52 y/o woman presents to you for routine f/u of her HTN, which is well-controlled on losartan. In review of her social hx, you note that she has abused oxycodone pills for almost 10 yrs- she gets them off the street and takes approximately five 10mg tablets per day. She has tried to stop “cold turkey” several times and experienced severe withdrawal symptoms- which caused her to resume taking the pills. She takes enough to prevent withdrawal and obtain a mild euphoria- but is not interested in “always being high.” She has not been able to hold a job due to this problem. She is very interested in getting help. She has never seen a counselor or been in any outpatient/inpatient drug programs. She has never used methadone. She tried buprenorphine on the street once a year ago, but it made her vomit and feel feverish, and she is very reluctant to try it again. Her last use of oxycodone was 3 hrs before her appt. She displays no signs or symptoms of intoxication or withdrawal. VS and physical exam are normal. She does NOT have insurance and has limited financial resources at this time.
Which of the following is/are the best option(s) in the management of this patient?
Inpatient detoxification program
Refer to comprehensive methadone program via your social worker
Prescribe methadone 30mg daily- goal of long-term maintenance
Prescribe buprenorphine/naloxone films – with goal of long-term maintenance
Prescribe buprenorphine films (without naloxone) – with goal of long-term maintenance
Prescribe naltrexone injection now
Referral to mental health/addiction counselor and/or group therapy
Prescribe clonidine taper for 5 days

## Slide 13
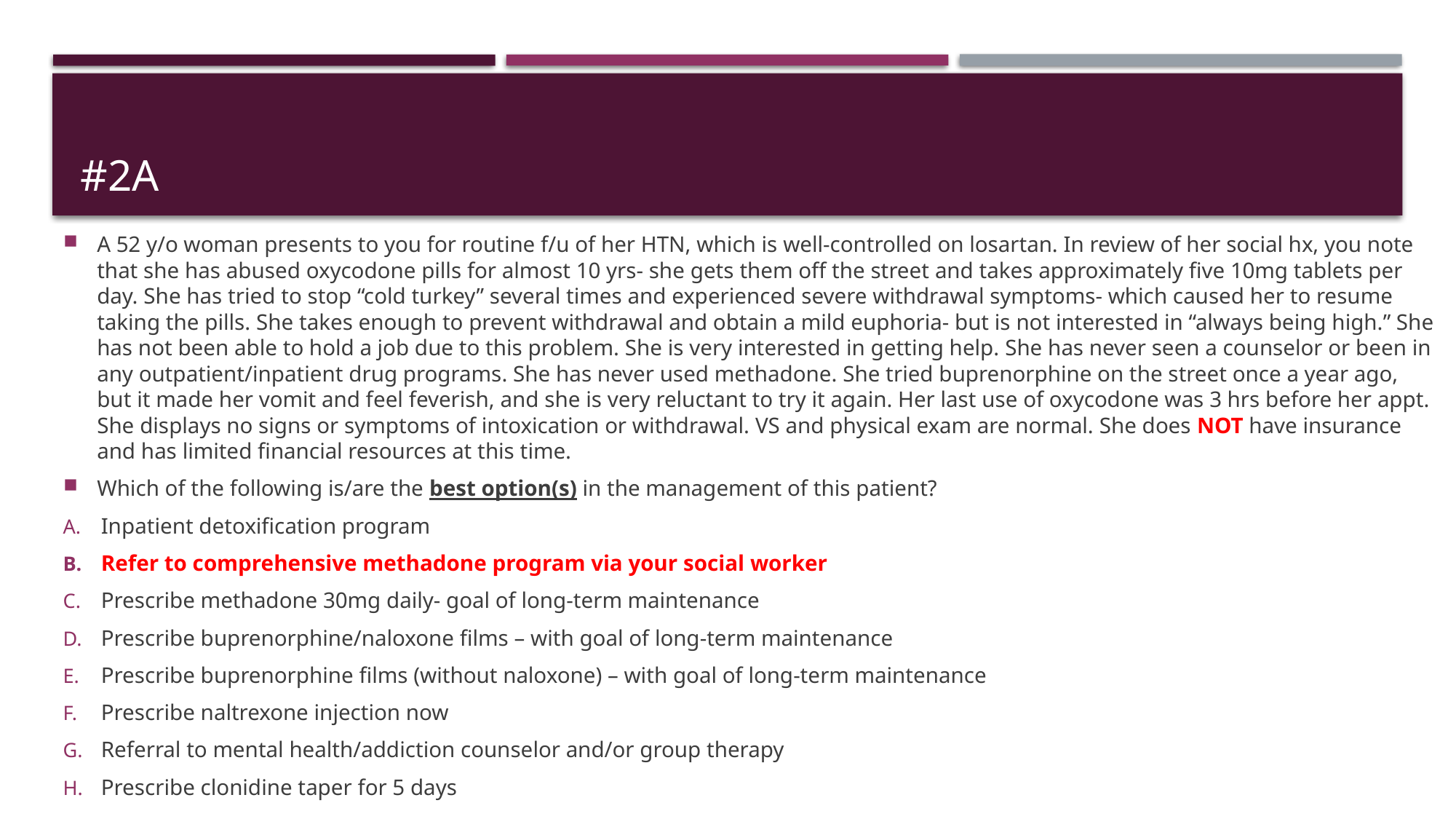

# #2A
A 52 y/o woman presents to you for routine f/u of her HTN, which is well-controlled on losartan. In review of her social hx, you note that she has abused oxycodone pills for almost 10 yrs- she gets them off the street and takes approximately five 10mg tablets per day. She has tried to stop “cold turkey” several times and experienced severe withdrawal symptoms- which caused her to resume taking the pills. She takes enough to prevent withdrawal and obtain a mild euphoria- but is not interested in “always being high.” She has not been able to hold a job due to this problem. She is very interested in getting help. She has never seen a counselor or been in any outpatient/inpatient drug programs. She has never used methadone. She tried buprenorphine on the street once a year ago, but it made her vomit and feel feverish, and she is very reluctant to try it again. Her last use of oxycodone was 3 hrs before her appt. She displays no signs or symptoms of intoxication or withdrawal. VS and physical exam are normal. She does NOT have insurance and has limited financial resources at this time.
Which of the following is/are the best option(s) in the management of this patient?
Inpatient detoxification program
Refer to comprehensive methadone program via your social worker
Prescribe methadone 30mg daily- goal of long-term maintenance
Prescribe buprenorphine/naloxone films – with goal of long-term maintenance
Prescribe buprenorphine films (without naloxone) – with goal of long-term maintenance
Prescribe naltrexone injection now
Referral to mental health/addiction counselor and/or group therapy
Prescribe clonidine taper for 5 days

## Slide 14
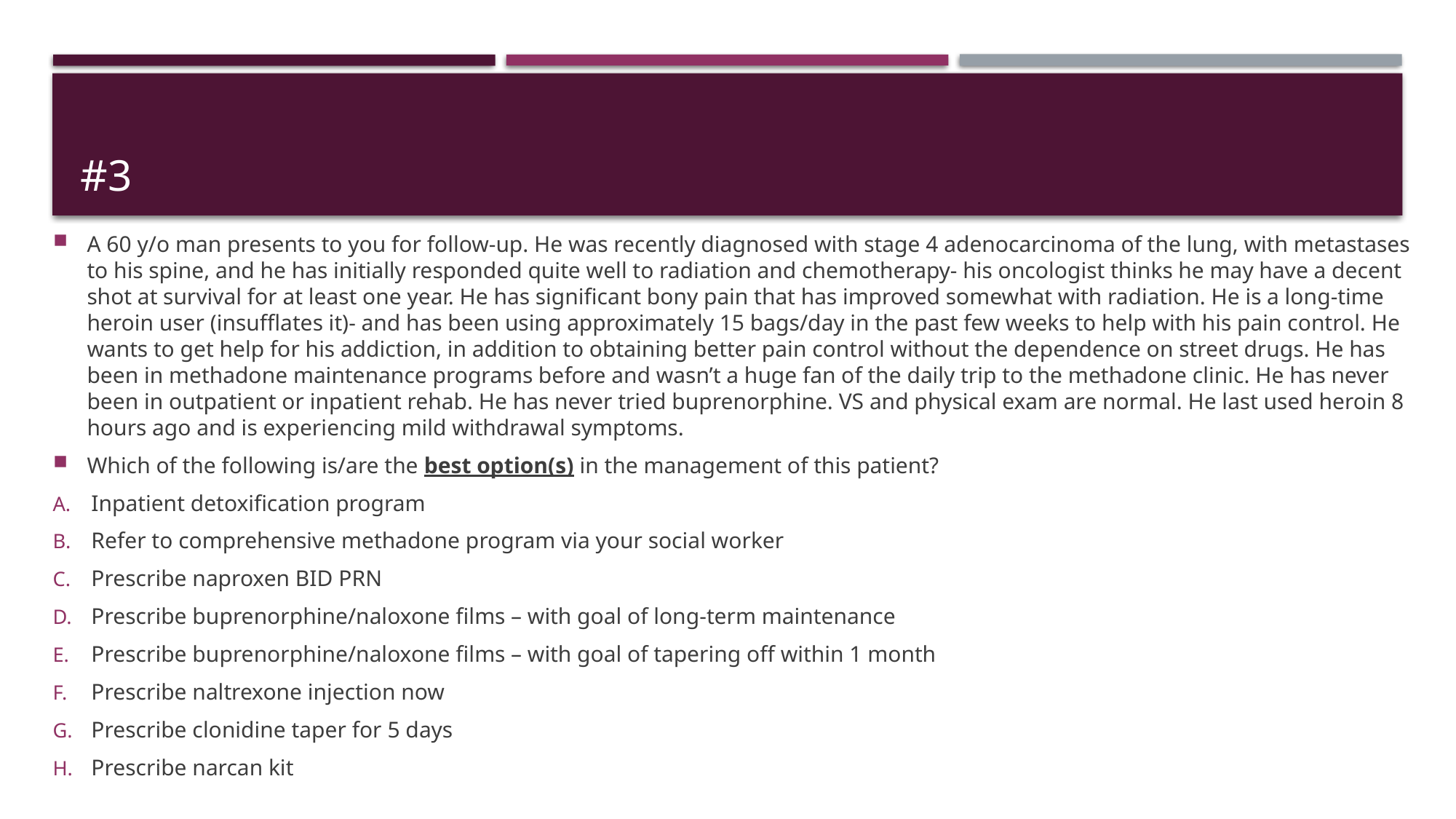

# #3
A 60 y/o man presents to you for follow-up. He was recently diagnosed with stage 4 adenocarcinoma of the lung, with metastases to his spine, and he has initially responded quite well to radiation and chemotherapy- his oncologist thinks he may have a decent shot at survival for at least one year. He has significant bony pain that has improved somewhat with radiation. He is a long-time heroin user (insufflates it)- and has been using approximately 15 bags/day in the past few weeks to help with his pain control. He wants to get help for his addiction, in addition to obtaining better pain control without the dependence on street drugs. He has been in methadone maintenance programs before and wasn’t a huge fan of the daily trip to the methadone clinic. He has never been in outpatient or inpatient rehab. He has never tried buprenorphine. VS and physical exam are normal. He last used heroin 8 hours ago and is experiencing mild withdrawal symptoms.
Which of the following is/are the best option(s) in the management of this patient?
Inpatient detoxification program
Refer to comprehensive methadone program via your social worker
Prescribe naproxen BID PRN
Prescribe buprenorphine/naloxone films – with goal of long-term maintenance
Prescribe buprenorphine/naloxone films – with goal of tapering off within 1 month
Prescribe naltrexone injection now
Prescribe clonidine taper for 5 days
Prescribe narcan kit

## Slide 15
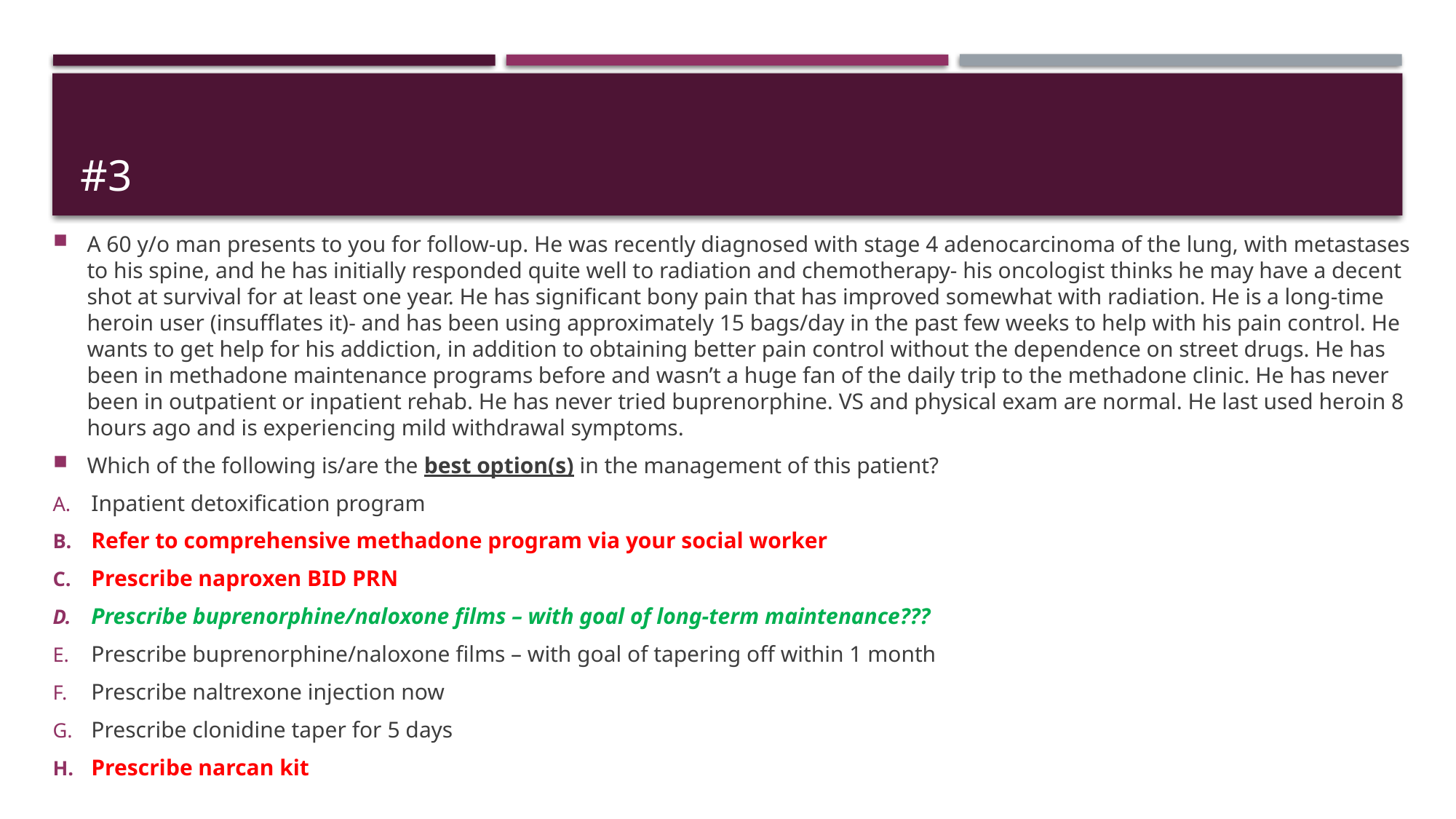

# #3
A 60 y/o man presents to you for follow-up. He was recently diagnosed with stage 4 adenocarcinoma of the lung, with metastases to his spine, and he has initially responded quite well to radiation and chemotherapy- his oncologist thinks he may have a decent shot at survival for at least one year. He has significant bony pain that has improved somewhat with radiation. He is a long-time heroin user (insufflates it)- and has been using approximately 15 bags/day in the past few weeks to help with his pain control. He wants to get help for his addiction, in addition to obtaining better pain control without the dependence on street drugs. He has been in methadone maintenance programs before and wasn’t a huge fan of the daily trip to the methadone clinic. He has never been in outpatient or inpatient rehab. He has never tried buprenorphine. VS and physical exam are normal. He last used heroin 8 hours ago and is experiencing mild withdrawal symptoms.
Which of the following is/are the best option(s) in the management of this patient?
Inpatient detoxification program
Refer to comprehensive methadone program via your social worker
Prescribe naproxen BID PRN
Prescribe buprenorphine/naloxone films – with goal of long-term maintenance???
Prescribe buprenorphine/naloxone films – with goal of tapering off within 1 month
Prescribe naltrexone injection now
Prescribe clonidine taper for 5 days
Prescribe narcan kit

## Slide 16
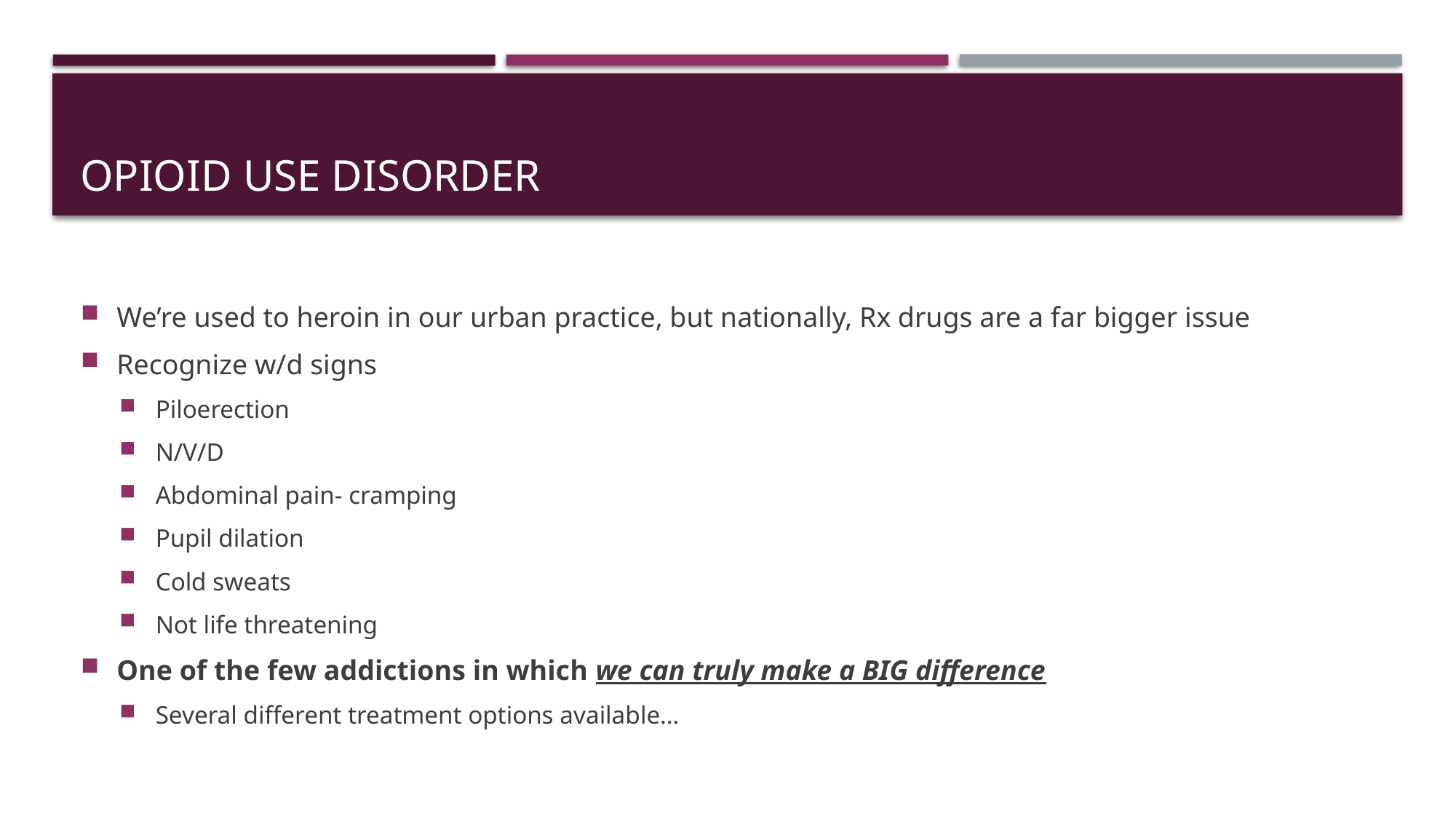

# Opioid use disorder
We’re used to heroin in our urban practice, but nationally, Rx drugs are a far bigger issue
Recognize w/d signs
Piloerection
N/V/D
Abdominal pain- cramping
Pupil dilation
Cold sweats
Not life threatening
One of the few addictions in which we can truly make a BIG difference
Several different treatment options available…

## Slide 17
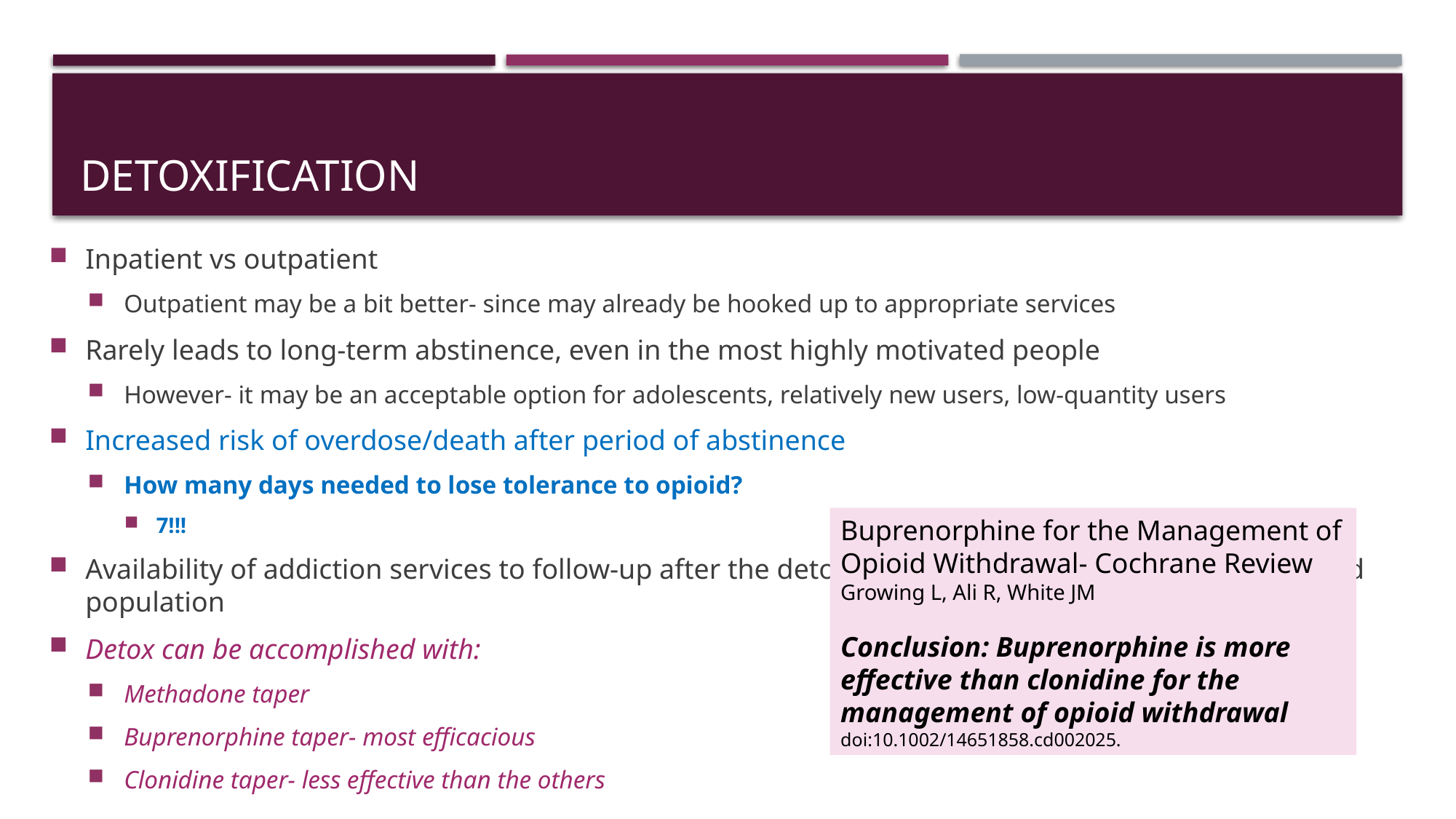

# Detoxification
Inpatient vs outpatient
Outpatient may be a bit better- since may already be hooked up to appropriate services
Rarely leads to long-term abstinence, even in the most highly motivated people
However- it may be an acceptable option for adolescents, relatively new users, low-quantity users
Increased risk of overdose/death after period of abstinence
How many days needed to lose tolerance to opioid?
7!!!
Availability of addiction services to follow-up after the detox is not robust, especially in an uninsured population
Detox can be accomplished with:
Methadone taper
Buprenorphine taper- most efficacious
Clonidine taper- less effective than the others
Buprenorphine for the Management of Opioid Withdrawal- Cochrane Review
Growing L, Ali R, White JM
Conclusion: Buprenorphine is more effective than clonidine for the management of opioid withdrawal
doi:10.1002/14651858.cd002025.

## Slide 18
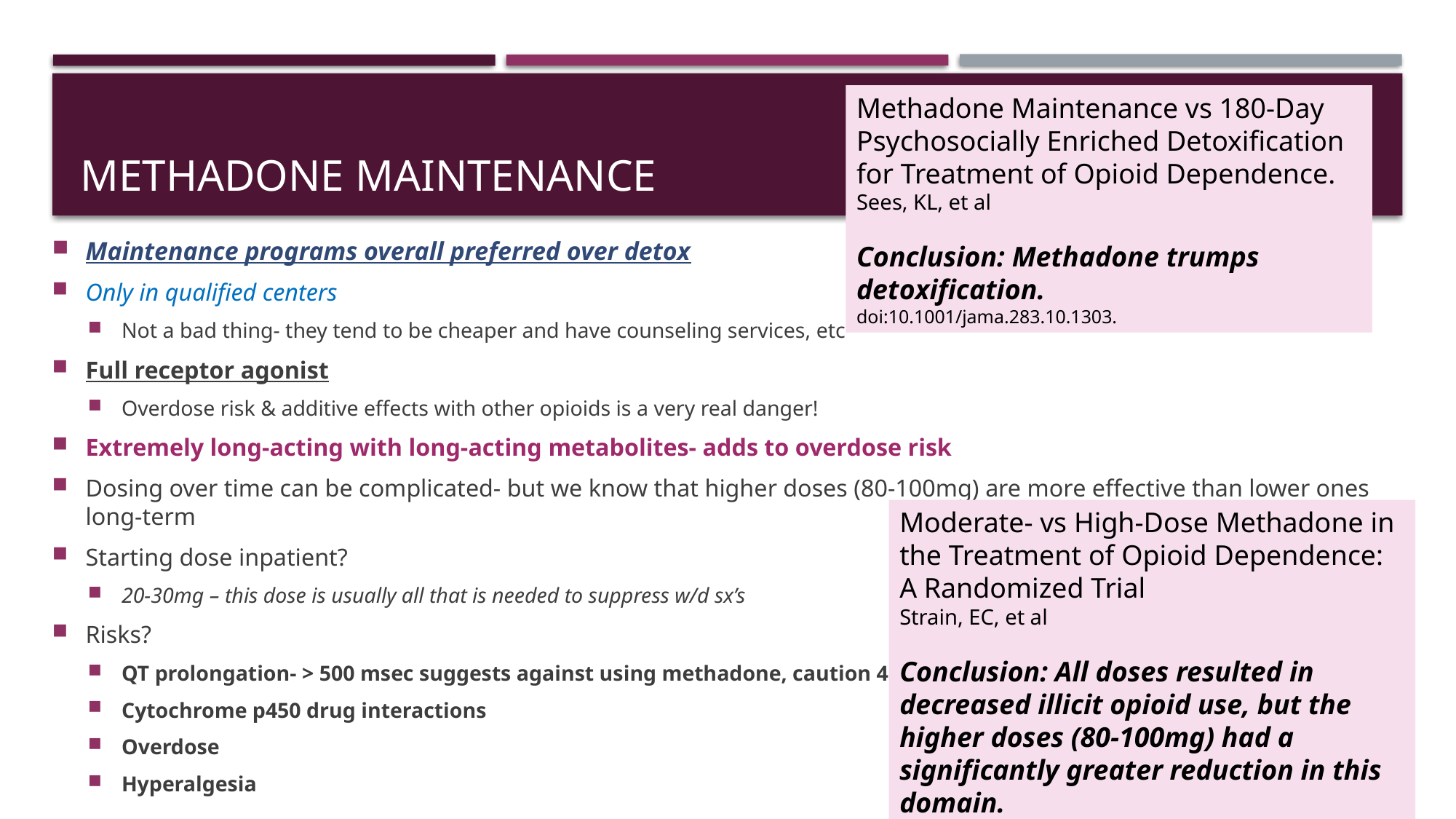

# Methadone maintenance
Methadone Maintenance vs 180-Day Psychosocially Enriched Detoxification for Treatment of Opioid Dependence.
Sees, KL, et al
Conclusion: Methadone trumps detoxification.
doi:10.1001/jama.283.10.1303.
Maintenance programs overall preferred over detox
Only in qualified centers
Not a bad thing- they tend to be cheaper and have counseling services, etc
Full receptor agonist
Overdose risk & additive effects with other opioids is a very real danger!
Extremely long-acting with long-acting metabolites- adds to overdose risk
Dosing over time can be complicated- but we know that higher doses (80-100mg) are more effective than lower ones long-term
Starting dose inpatient?
20-30mg – this dose is usually all that is needed to suppress w/d sx’s
Risks?
QT prolongation- > 500 msec suggests against using methadone, caution 450-500
Cytochrome p450 drug interactions
Overdose
Hyperalgesia
Moderate- vs High-Dose Methadone in the Treatment of Opioid Dependence: A Randomized Trial
Strain, EC, et al
Conclusion: All doses resulted in decreased illicit opioid use, but the higher doses (80-100mg) had a significantly greater reduction in this domain.
doi:10.1001/jama.281.11.1000.

## Slide 19
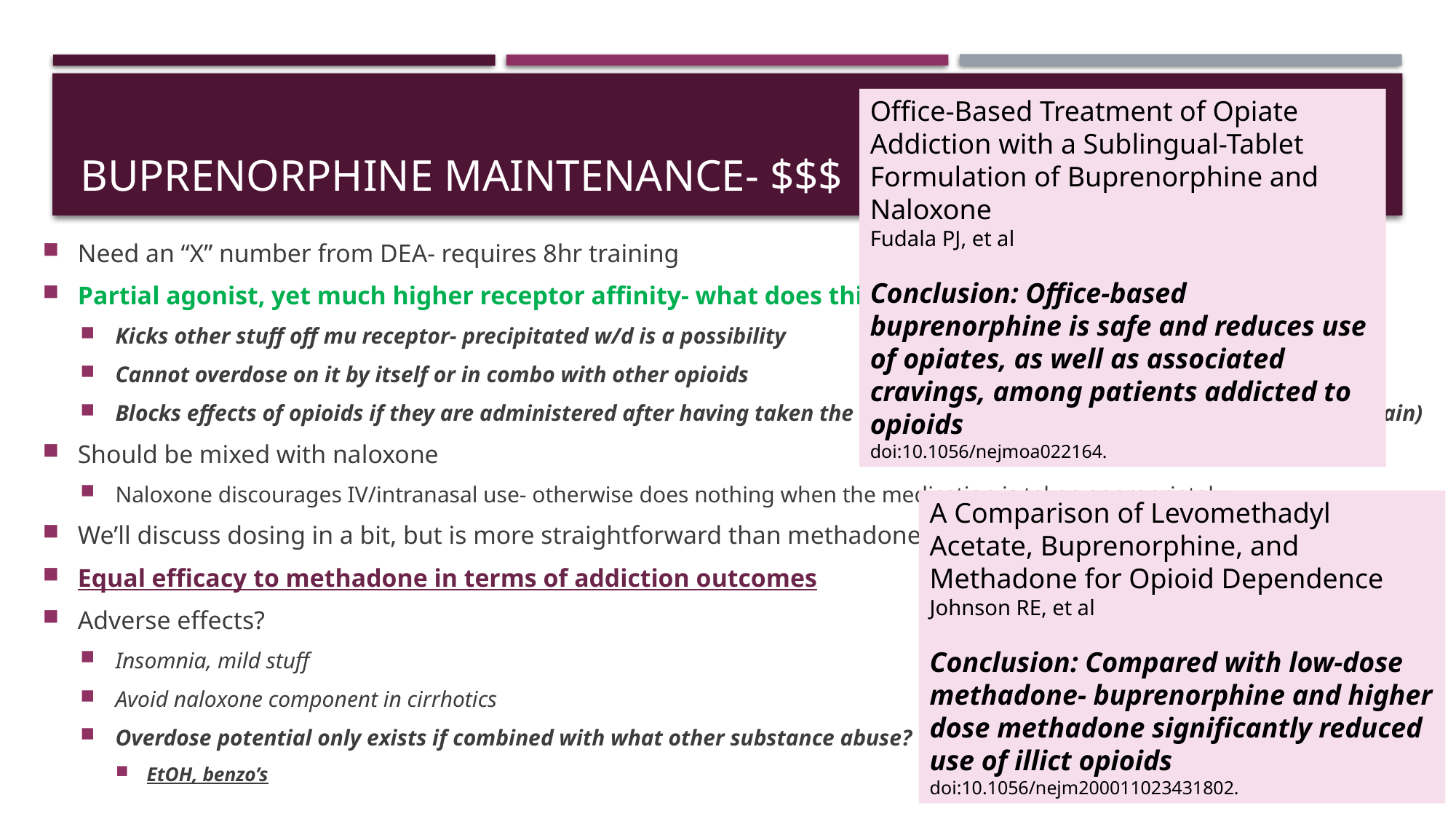

# Buprenorphine maintenance- $$$
Office-Based Treatment of Opiate Addiction with a Sublingual-Tablet Formulation of Buprenorphine and Naloxone
Fudala PJ, et al
Conclusion: Office-based buprenorphine is safe and reduces use of opiates, as well as associated cravings, among patients addicted to opioids
doi:10.1056/nejmoa022164.
Need an “X” number from DEA- requires 8hr training
Partial agonist, yet much higher receptor affinity- what does this mean?
Kicks other stuff off mu receptor- precipitated w/d is a possibility
Cannot overdose on it by itself or in combo with other opioids
Blocks effects of opioids if they are administered after having taken the buprenorphine (this is bad in the setting of acute pain)
Should be mixed with naloxone
Naloxone discourages IV/intranasal use- otherwise does nothing when the medication is taken appropriately
We’ll discuss dosing in a bit, but is more straightforward than methadone
Equal efficacy to methadone in terms of addiction outcomes
Adverse effects?
Insomnia, mild stuff
Avoid naloxone component in cirrhotics
Overdose potential only exists if combined with what other substance abuse?
EtOH, benzo’s
A Comparison of Levomethadyl Acetate, Buprenorphine, and Methadone for Opioid Dependence
Johnson RE, et al
Conclusion: Compared with low-dose methadone- buprenorphine and higher dose methadone significantly reduced use of illict opioids
doi:10.1056/nejm200011023431802.

## Slide 20
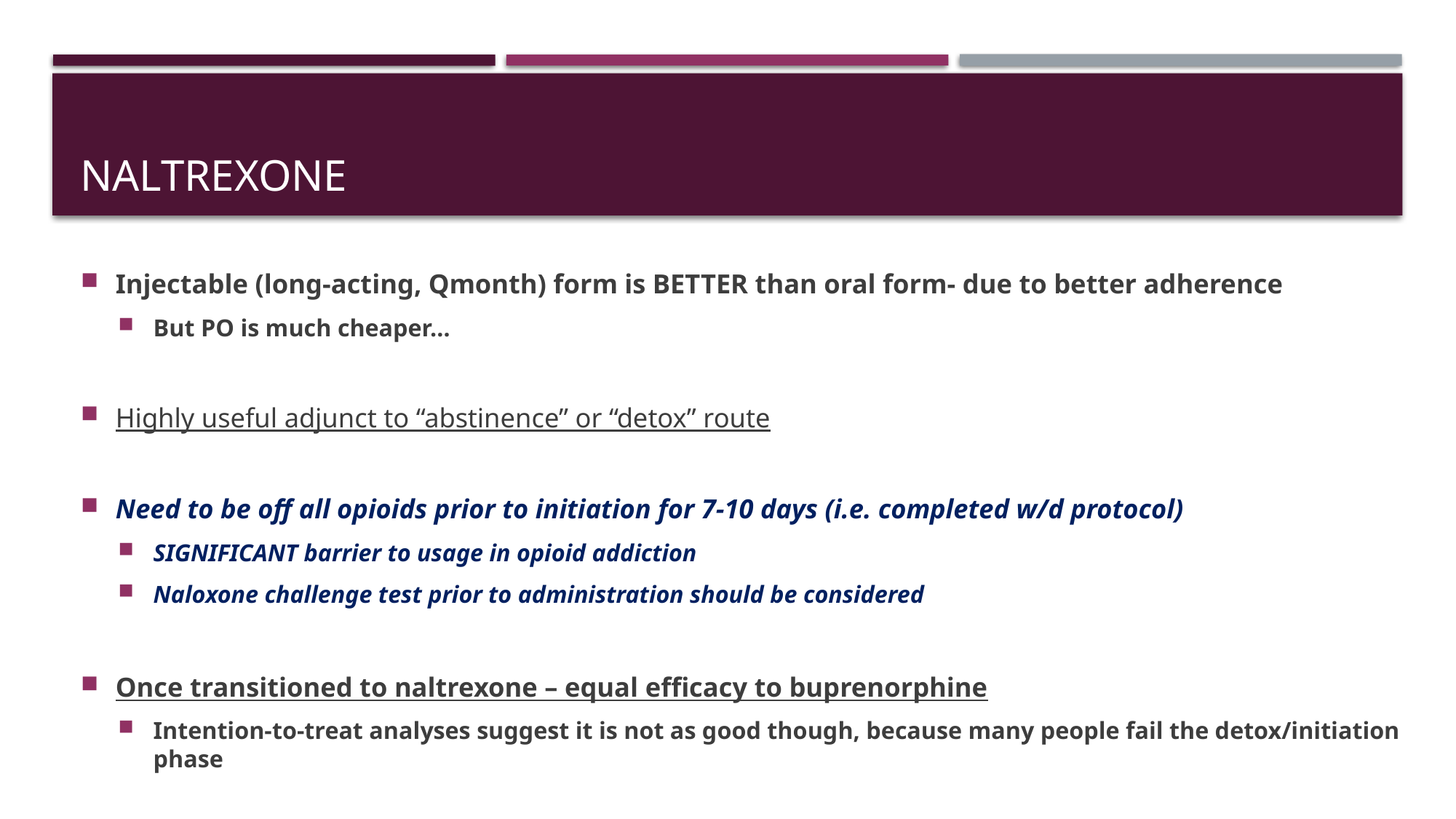

# Naltrexone
Injectable (long-acting, Qmonth) form is BETTER than oral form- due to better adherence
But PO is much cheaper…
Highly useful adjunct to “abstinence” or “detox” route
Need to be off all opioids prior to initiation for 7-10 days (i.e. completed w/d protocol)
SIGNIFICANT barrier to usage in opioid addiction
Naloxone challenge test prior to administration should be considered
Once transitioned to naltrexone – equal efficacy to buprenorphine
Intention-to-treat analyses suggest it is not as good though, because many people fail the detox/initiation phase

## Slide 21
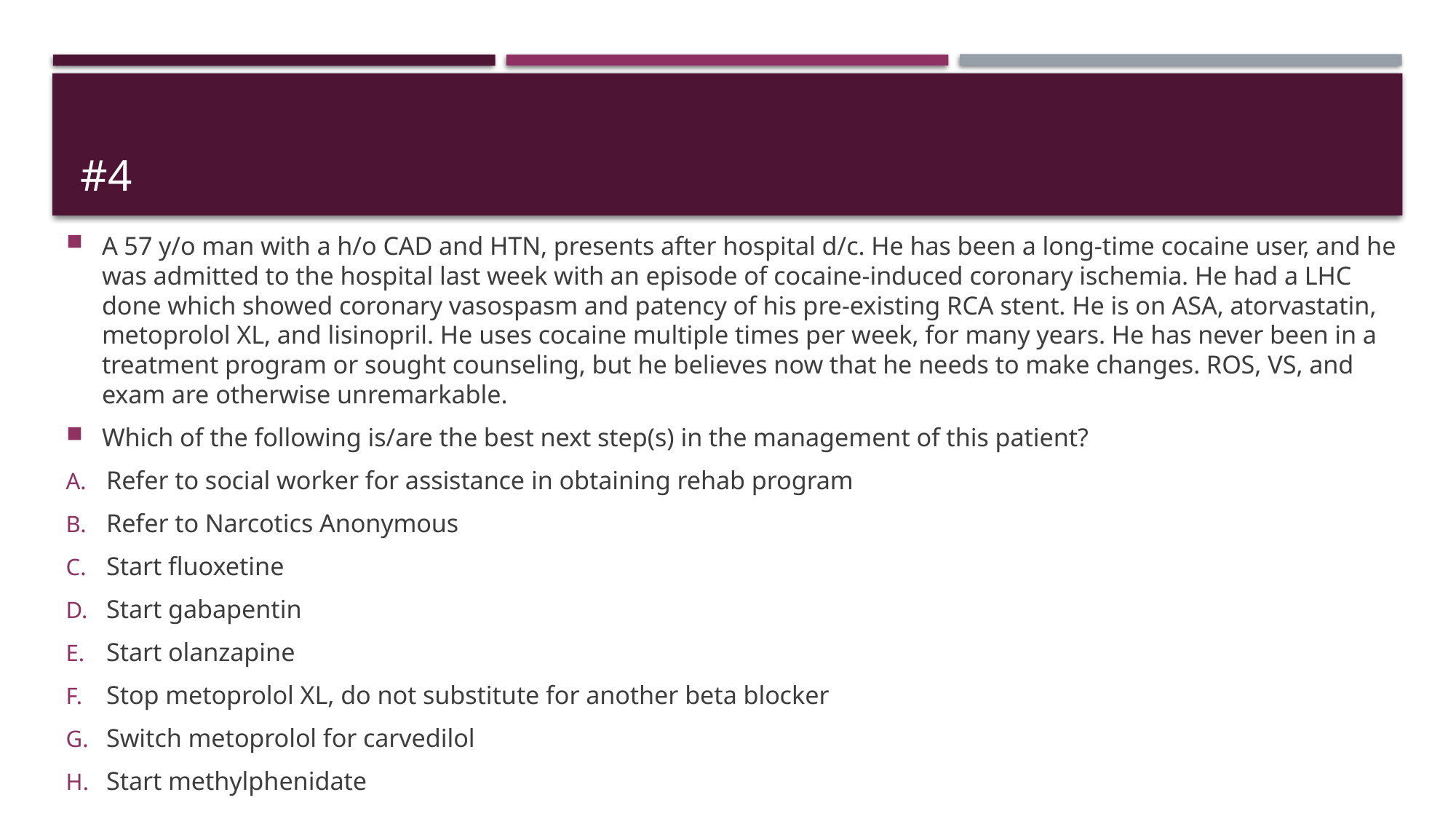

# #4
A 57 y/o man with a h/o CAD and HTN, presents after hospital d/c. He has been a long-time cocaine user, and he was admitted to the hospital last week with an episode of cocaine-induced coronary ischemia. He had a LHC done which showed coronary vasospasm and patency of his pre-existing RCA stent. He is on ASA, atorvastatin, metoprolol XL, and lisinopril. He uses cocaine multiple times per week, for many years. He has never been in a treatment program or sought counseling, but he believes now that he needs to make changes. ROS, VS, and exam are otherwise unremarkable.
Which of the following is/are the best next step(s) in the management of this patient?
Refer to social worker for assistance in obtaining rehab program
Refer to Narcotics Anonymous
Start fluoxetine
Start gabapentin
Start olanzapine
Stop metoprolol XL, do not substitute for another beta blocker
Switch metoprolol for carvedilol
Start methylphenidate

## Slide 22
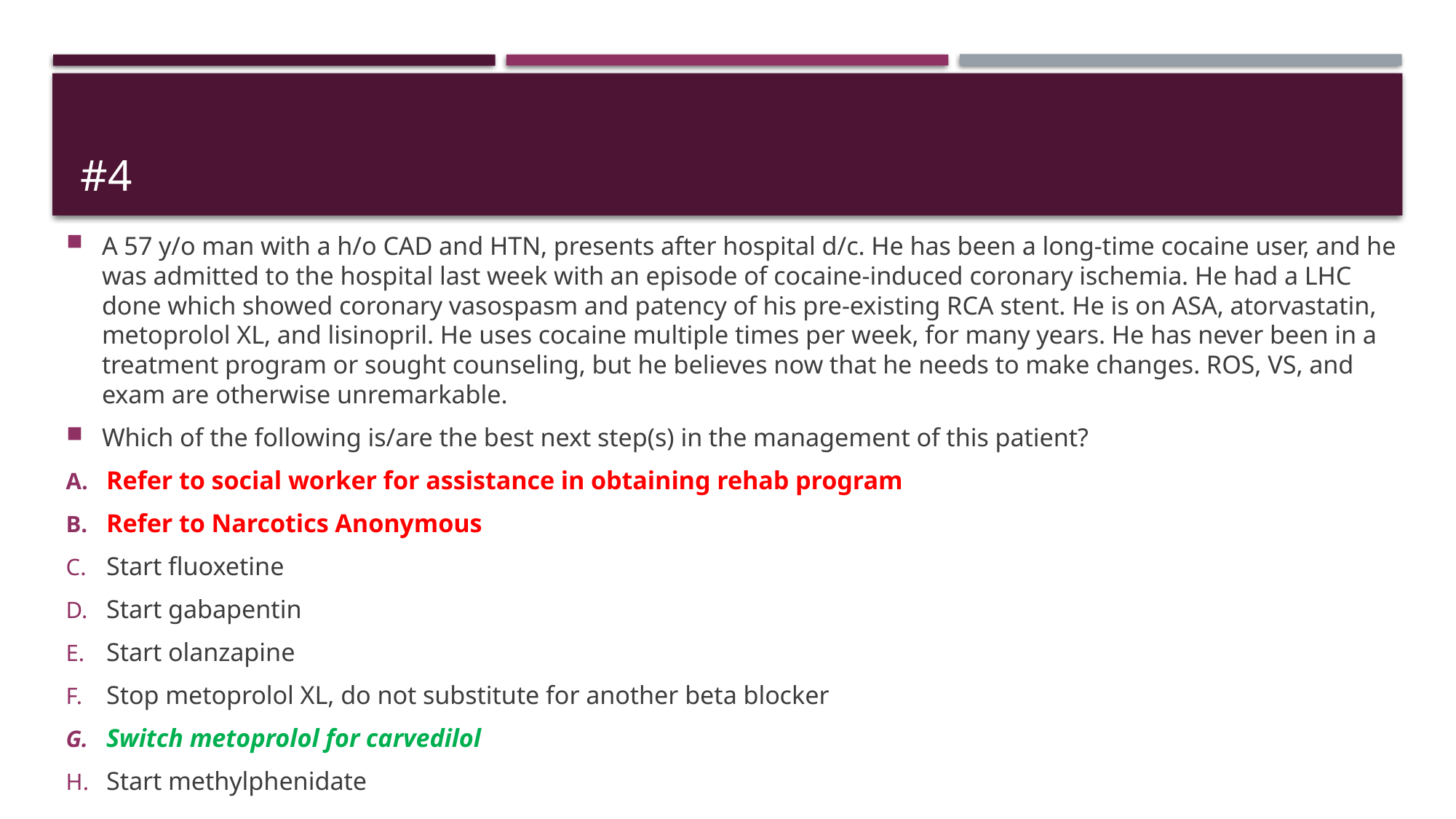

# #4
A 57 y/o man with a h/o CAD and HTN, presents after hospital d/c. He has been a long-time cocaine user, and he was admitted to the hospital last week with an episode of cocaine-induced coronary ischemia. He had a LHC done which showed coronary vasospasm and patency of his pre-existing RCA stent. He is on ASA, atorvastatin, metoprolol XL, and lisinopril. He uses cocaine multiple times per week, for many years. He has never been in a treatment program or sought counseling, but he believes now that he needs to make changes. ROS, VS, and exam are otherwise unremarkable.
Which of the following is/are the best next step(s) in the management of this patient?
Refer to social worker for assistance in obtaining rehab program
Refer to Narcotics Anonymous
Start fluoxetine
Start gabapentin
Start olanzapine
Stop metoprolol XL, do not substitute for another beta blocker
Switch metoprolol for carvedilol
Start methylphenidate

## Slide 23
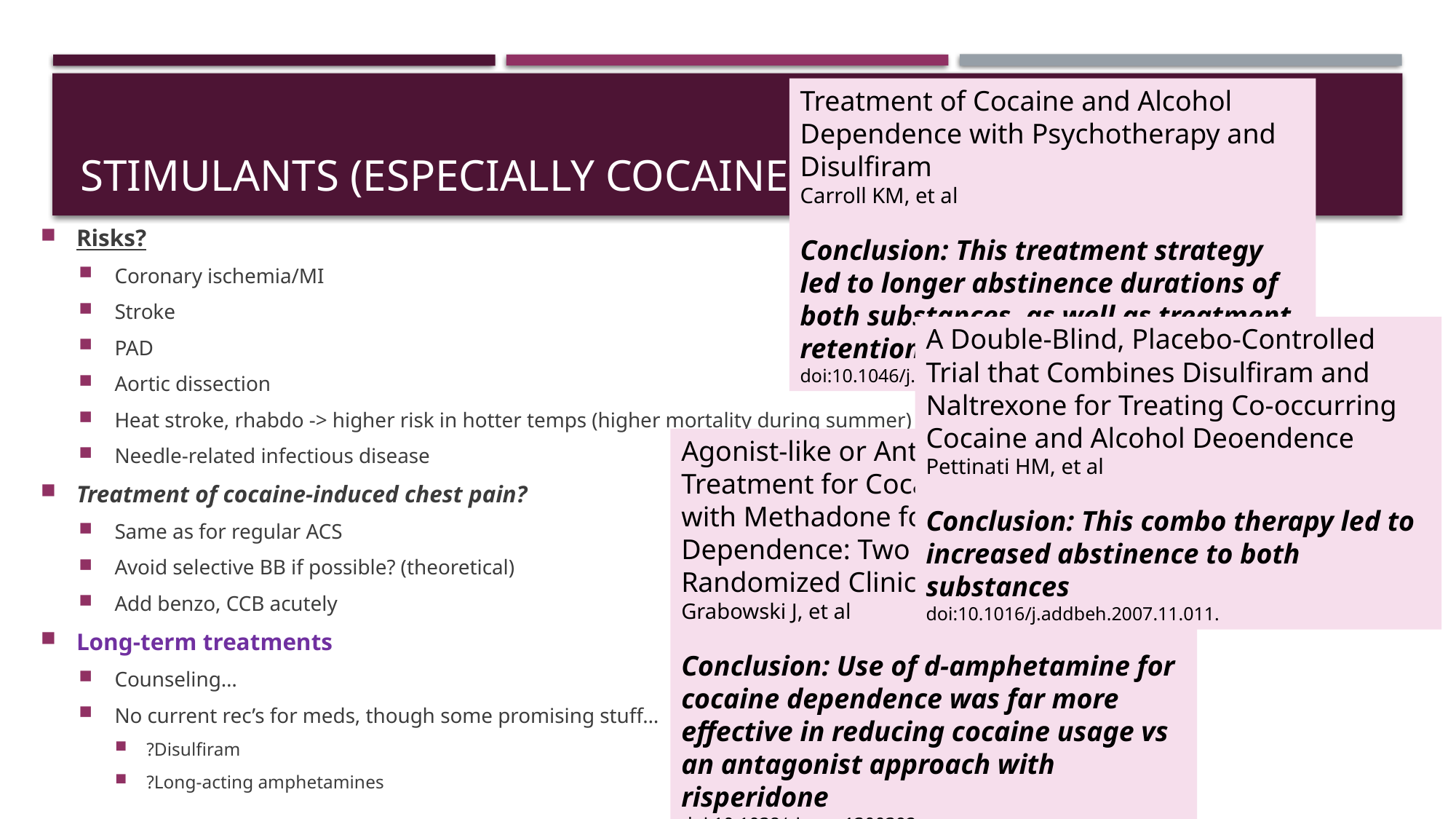

Treatment of Cocaine and Alcohol Dependence with Psychotherapy and Disulfiram
Carroll KM, et al
Conclusion: This treatment strategy led to longer abstinence durations of both substances, as well as treatment retention
doi:10.1046/j.1360-0443.1998.9357137.x.
# Stimulants (especially cocaine)
Risks?
Coronary ischemia/MI
Stroke
PAD
Aortic dissection
Heat stroke, rhabdo -> higher risk in hotter temps (higher mortality during summer)
Needle-related infectious disease
Treatment of cocaine-induced chest pain?
Same as for regular ACS
Avoid selective BB if possible? (theoretical)
Add benzo, CCB acutely
Long-term treatments
Counseling…
No current rec’s for meds, though some promising stuff…
?Disulfiram
?Long-acting amphetamines
A Double-Blind, Placebo-Controlled Trial that Combines Disulfiram and Naltrexone for Treating Co-occurring Cocaine and Alcohol Deoendence
Pettinati HM, et al
Conclusion: This combo therapy led to increased abstinence to both substances
doi:10.1016/j.addbeh.2007.11.011.
Agonist-like or Antagonist-like Treatment for Cocaine Dependence with Methadone for Heroin Dependence: Two Double-Blind Randomized Clinical Trials
Grabowski J, et al
Conclusion: Use of d-amphetamine for cocaine dependence was far more effective in reducing cocaine usage vs an antagonist approach with risperidone
doi:10.1038/sj.npp.1300392.

## Slide 24
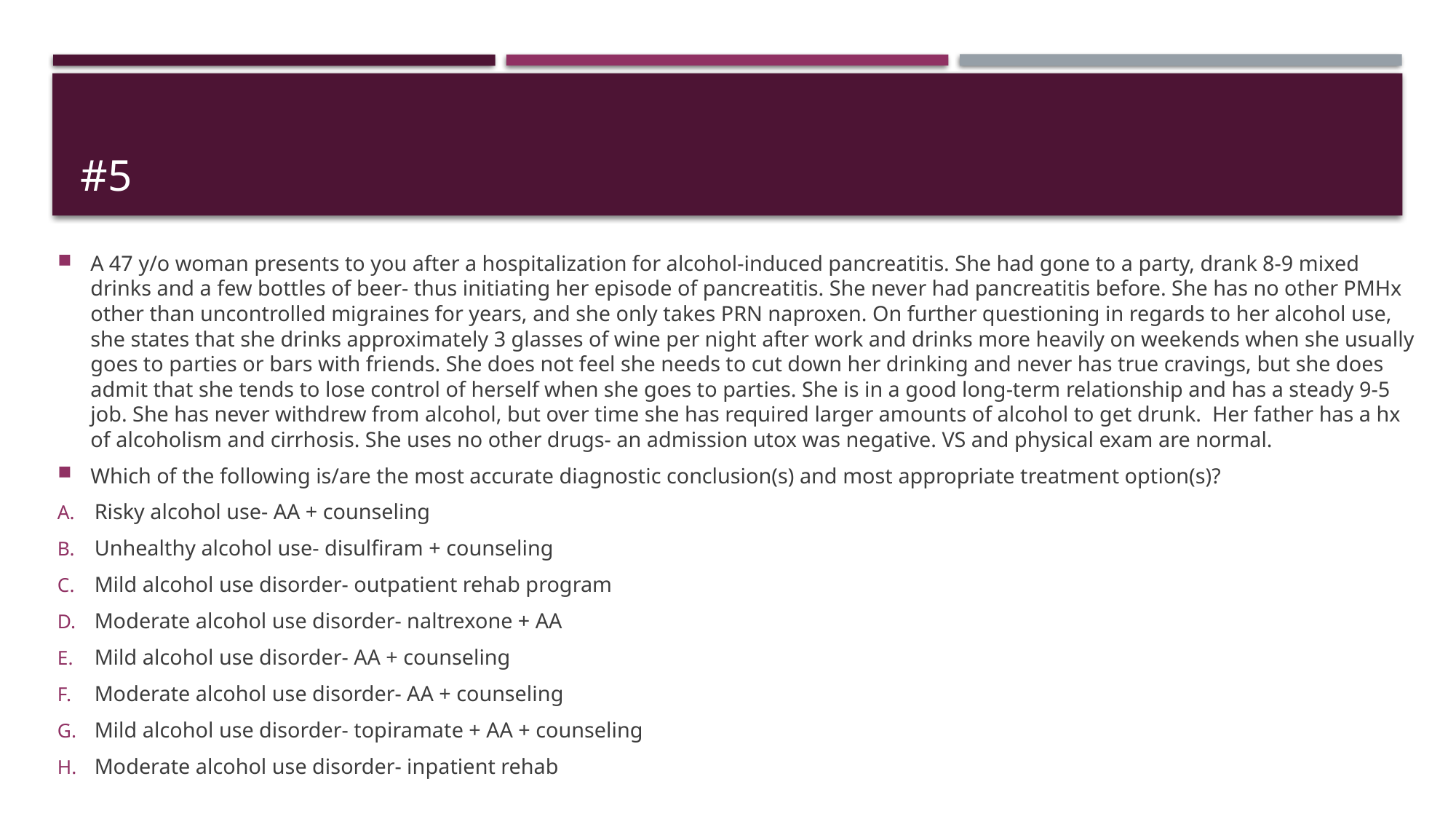

# #5
A 47 y/o woman presents to you after a hospitalization for alcohol-induced pancreatitis. She had gone to a party, drank 8-9 mixed drinks and a few bottles of beer- thus initiating her episode of pancreatitis. She never had pancreatitis before. She has no other PMHx other than uncontrolled migraines for years, and she only takes PRN naproxen. On further questioning in regards to her alcohol use, she states that she drinks approximately 3 glasses of wine per night after work and drinks more heavily on weekends when she usually goes to parties or bars with friends. She does not feel she needs to cut down her drinking and never has true cravings, but she does admit that she tends to lose control of herself when she goes to parties. She is in a good long-term relationship and has a steady 9-5 job. She has never withdrew from alcohol, but over time she has required larger amounts of alcohol to get drunk. Her father has a hx of alcoholism and cirrhosis. She uses no other drugs- an admission utox was negative. VS and physical exam are normal.
Which of the following is/are the most accurate diagnostic conclusion(s) and most appropriate treatment option(s)?
Risky alcohol use- AA + counseling
Unhealthy alcohol use- disulfiram + counseling
Mild alcohol use disorder- outpatient rehab program
Moderate alcohol use disorder- naltrexone + AA
Mild alcohol use disorder- AA + counseling
Moderate alcohol use disorder- AA + counseling
Mild alcohol use disorder- topiramate + AA + counseling
Moderate alcohol use disorder- inpatient rehab

## Slide 25
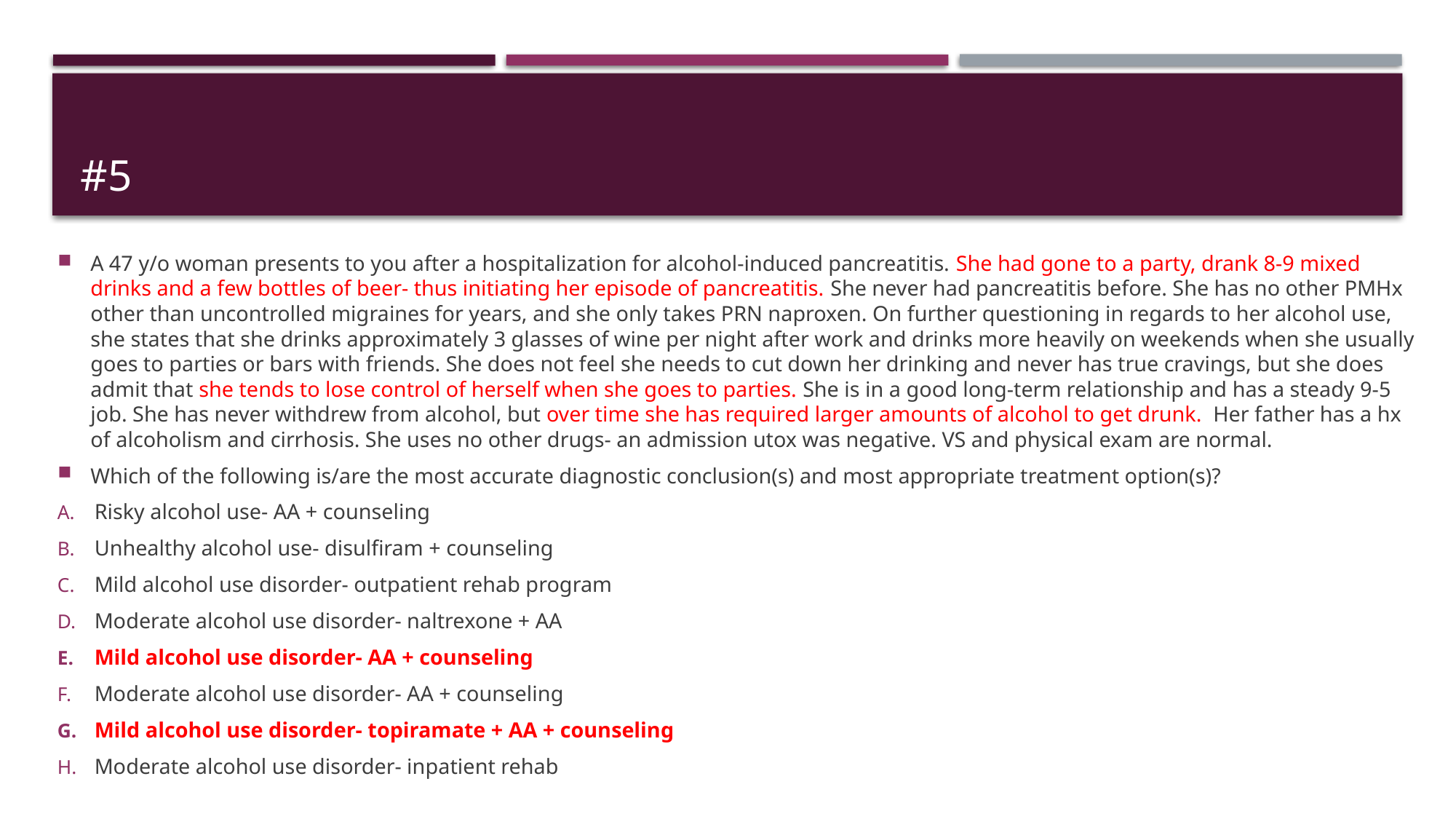

# #5
A 47 y/o woman presents to you after a hospitalization for alcohol-induced pancreatitis. She had gone to a party, drank 8-9 mixed drinks and a few bottles of beer- thus initiating her episode of pancreatitis. She never had pancreatitis before. She has no other PMHx other than uncontrolled migraines for years, and she only takes PRN naproxen. On further questioning in regards to her alcohol use, she states that she drinks approximately 3 glasses of wine per night after work and drinks more heavily on weekends when she usually goes to parties or bars with friends. She does not feel she needs to cut down her drinking and never has true cravings, but she does admit that she tends to lose control of herself when she goes to parties. She is in a good long-term relationship and has a steady 9-5 job. She has never withdrew from alcohol, but over time she has required larger amounts of alcohol to get drunk. Her father has a hx of alcoholism and cirrhosis. She uses no other drugs- an admission utox was negative. VS and physical exam are normal.
Which of the following is/are the most accurate diagnostic conclusion(s) and most appropriate treatment option(s)?
Risky alcohol use- AA + counseling
Unhealthy alcohol use- disulfiram + counseling
Mild alcohol use disorder- outpatient rehab program
Moderate alcohol use disorder- naltrexone + AA
Mild alcohol use disorder- AA + counseling
Moderate alcohol use disorder- AA + counseling
Mild alcohol use disorder- topiramate + AA + counseling
Moderate alcohol use disorder- inpatient rehab

## Slide 26
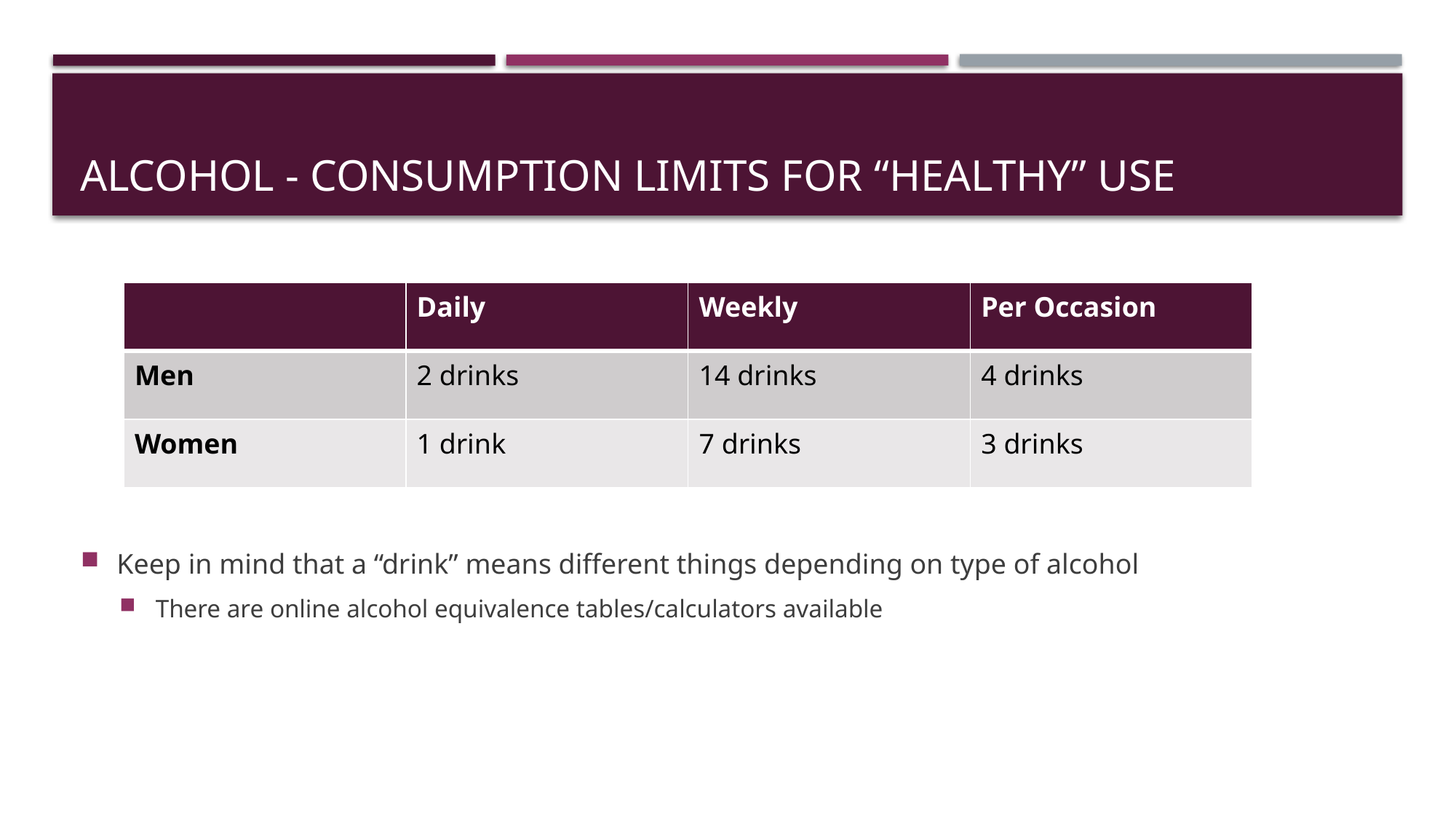

# Alcohol - consumption limits for “healthy” use
| | Daily | Weekly | Per Occasion |
| --- | --- | --- | --- |
| Men | 2 drinks | 14 drinks | 4 drinks |
| Women | 1 drink | 7 drinks | 3 drinks |
Keep in mind that a “drink” means different things depending on type of alcohol
There are online alcohol equivalence tables/calculators available

## Slide 27
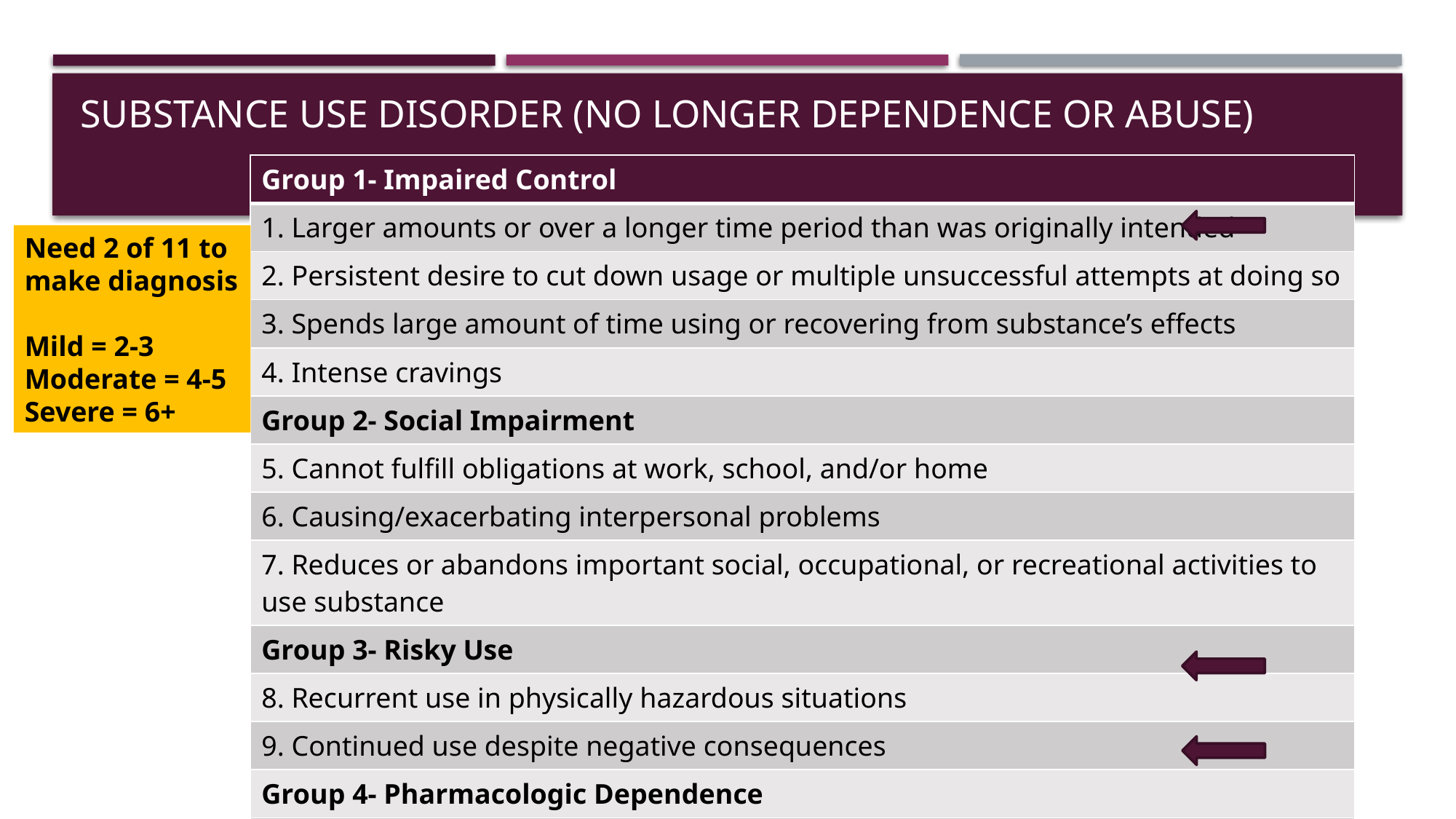

# Substance Use Disorder (no longer dependence or abuse)
| Group 1- Impaired Control |
| --- |
| 1. Larger amounts or over a longer time period than was originally intended |
| 2. Persistent desire to cut down usage or multiple unsuccessful attempts at doing so |
| 3. Spends large amount of time using or recovering from substance’s effects |
| 4. Intense cravings |
| Group 2- Social Impairment |
| 5. Cannot fulfill obligations at work, school, and/or home |
| 6. Causing/exacerbating interpersonal problems |
| 7. Reduces or abandons important social, occupational, or recreational activities to use substance |
| Group 3- Risky Use |
| 8. Recurrent use in physically hazardous situations |
| 9. Continued use despite negative consequences |
| Group 4- Pharmacologic Dependence |
| 10. Tolerance |
| 11. Withdrawal |
Need 2 of 11 to make diagnosis
Mild = 2-3
Moderate = 4-5
Severe = 6+

## Slide 28
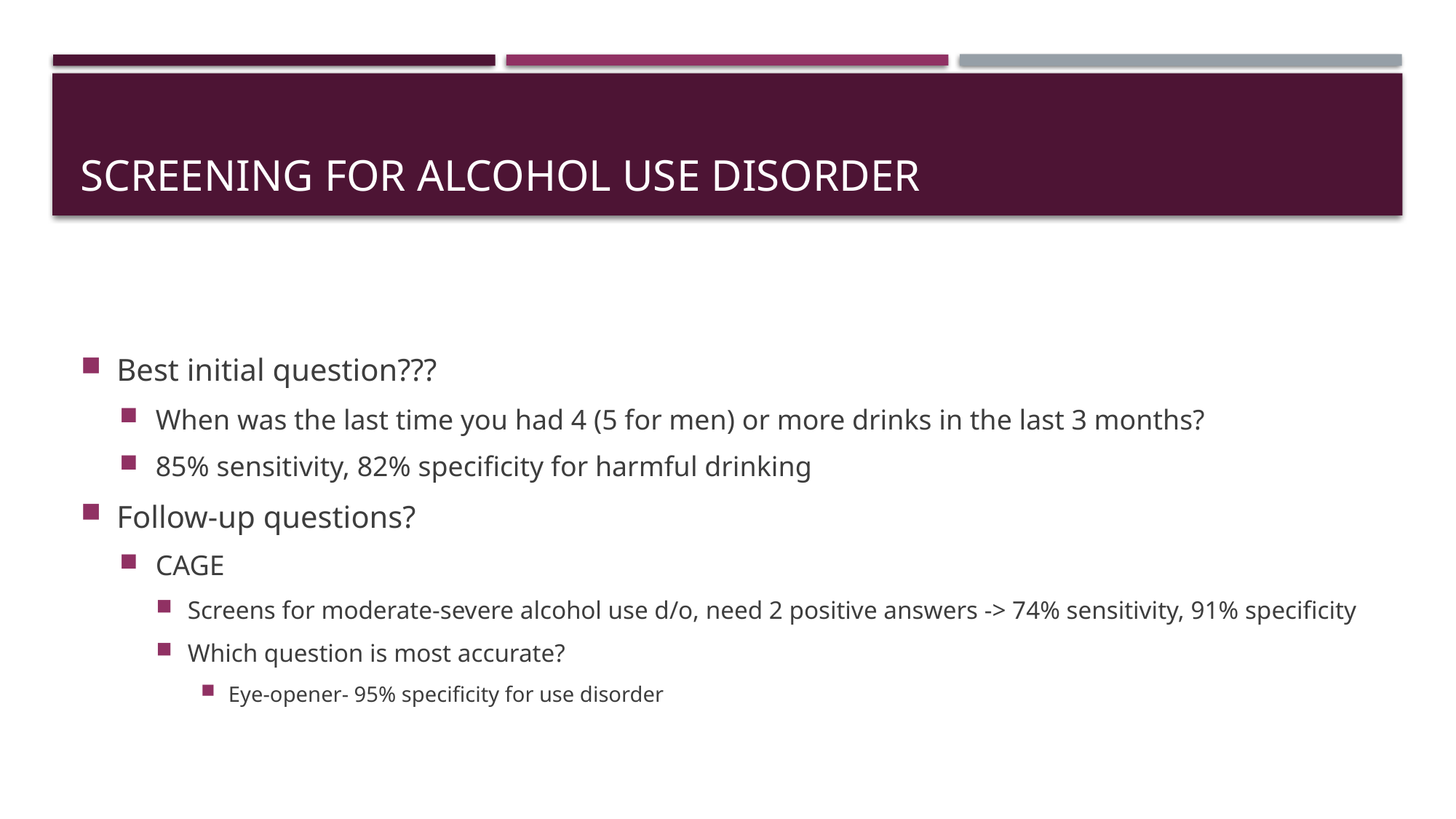

# Screening for alcohol use disorder
Best initial question???
When was the last time you had 4 (5 for men) or more drinks in the last 3 months?
85% sensitivity, 82% specificity for harmful drinking
Follow-up questions?
CAGE
Screens for moderate-severe alcohol use d/o, need 2 positive answers -> 74% sensitivity, 91% specificity
Which question is most accurate?
Eye-opener- 95% specificity for use disorder

## Slide 29
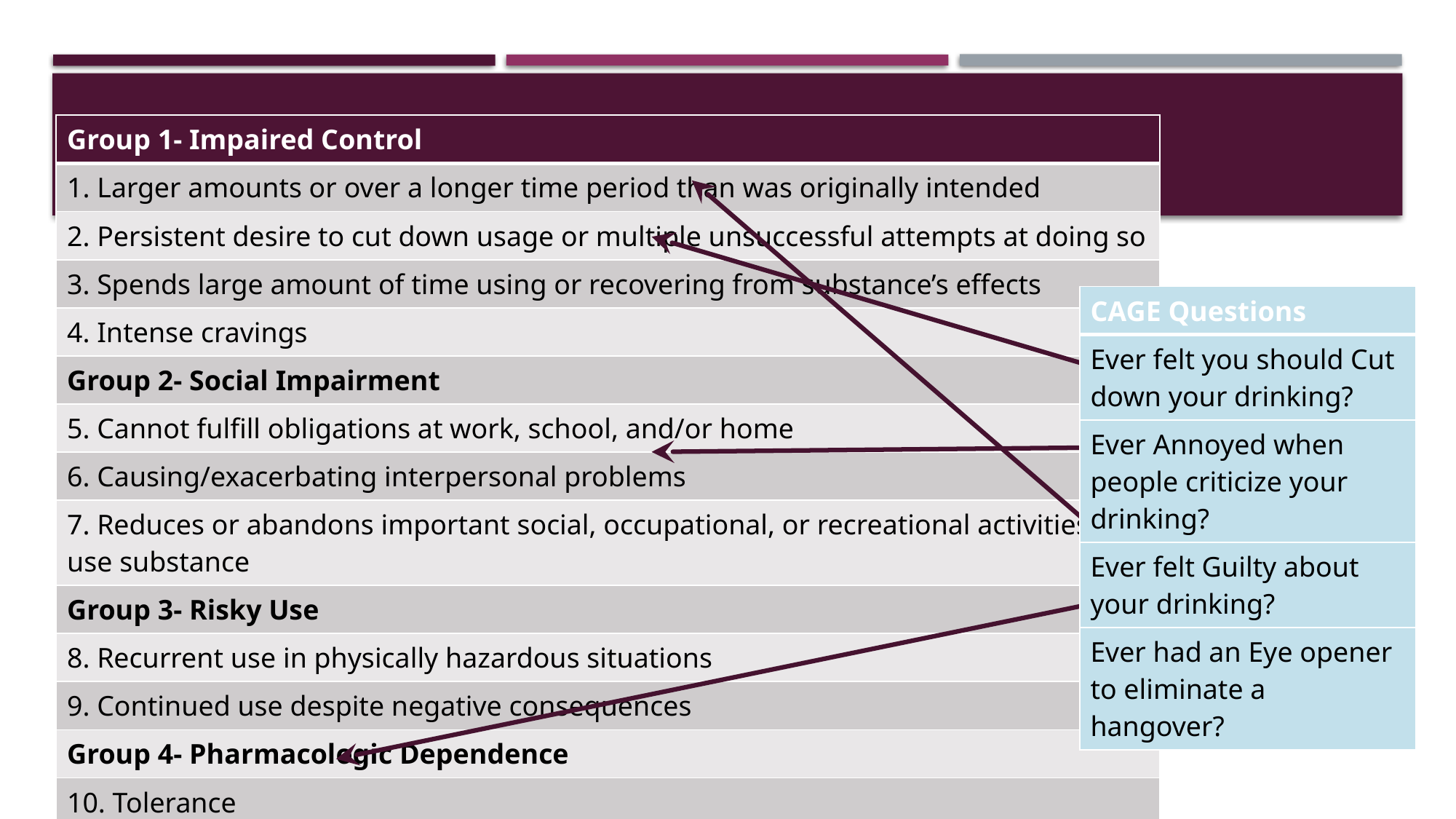

#
| Group 1- Impaired Control |
| --- |
| 1. Larger amounts or over a longer time period than was originally intended |
| 2. Persistent desire to cut down usage or multiple unsuccessful attempts at doing so |
| 3. Spends large amount of time using or recovering from substance’s effects |
| 4. Intense cravings |
| Group 2- Social Impairment |
| 5. Cannot fulfill obligations at work, school, and/or home |
| 6. Causing/exacerbating interpersonal problems |
| 7. Reduces or abandons important social, occupational, or recreational activities to use substance |
| Group 3- Risky Use |
| 8. Recurrent use in physically hazardous situations |
| 9. Continued use despite negative consequences |
| Group 4- Pharmacologic Dependence |
| 10. Tolerance |
| 11. Withdrawal |
| CAGE Questions |
| --- |
| Ever felt you should Cut down your drinking? |
| Ever Annoyed when people criticize your drinking? |
| Ever felt Guilty about your drinking? |
| Ever had an Eye opener to eliminate a hangover? |

## Slide 30
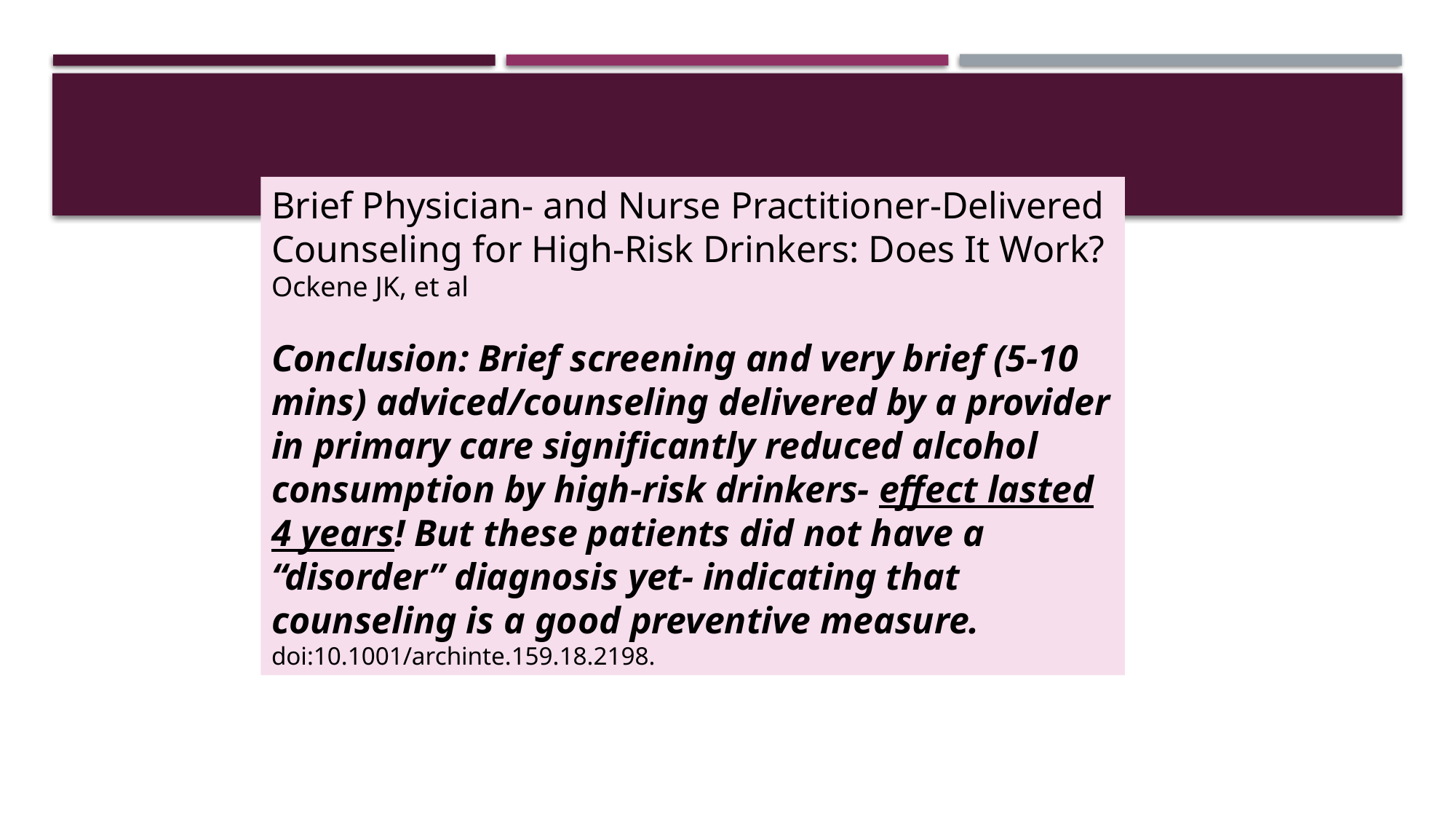

#
Brief Physician- and Nurse Practitioner-Delivered Counseling for High-Risk Drinkers: Does It Work?
Ockene JK, et al
Conclusion: Brief screening and very brief (5-10 mins) adviced/counseling delivered by a provider in primary care significantly reduced alcohol consumption by high-risk drinkers- effect lasted 4 years! But these patients did not have a “disorder” diagnosis yet- indicating that counseling is a good preventive measure.
doi:10.1001/archinte.159.18.2198.

## Slide 31
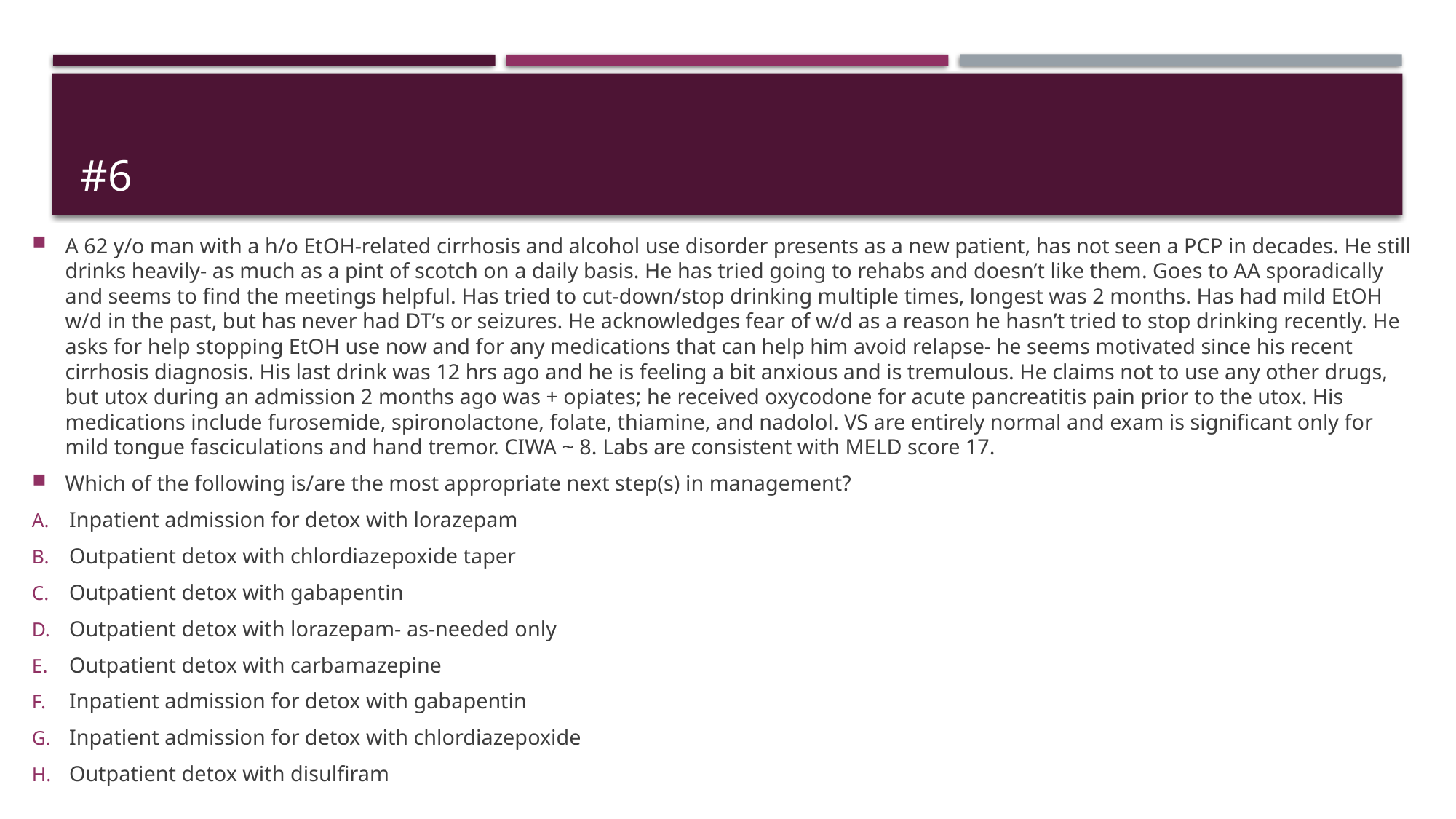

# #6
A 62 y/o man with a h/o EtOH-related cirrhosis and alcohol use disorder presents as a new patient, has not seen a PCP in decades. He still drinks heavily- as much as a pint of scotch on a daily basis. He has tried going to rehabs and doesn’t like them. Goes to AA sporadically and seems to find the meetings helpful. Has tried to cut-down/stop drinking multiple times, longest was 2 months. Has had mild EtOH w/d in the past, but has never had DT’s or seizures. He acknowledges fear of w/d as a reason he hasn’t tried to stop drinking recently. He asks for help stopping EtOH use now and for any medications that can help him avoid relapse- he seems motivated since his recent cirrhosis diagnosis. His last drink was 12 hrs ago and he is feeling a bit anxious and is tremulous. He claims not to use any other drugs, but utox during an admission 2 months ago was + opiates; he received oxycodone for acute pancreatitis pain prior to the utox. His medications include furosemide, spironolactone, folate, thiamine, and nadolol. VS are entirely normal and exam is significant only for mild tongue fasciculations and hand tremor. CIWA ~ 8. Labs are consistent with MELD score 17.
Which of the following is/are the most appropriate next step(s) in management?
Inpatient admission for detox with lorazepam
Outpatient detox with chlordiazepoxide taper
Outpatient detox with gabapentin
Outpatient detox with lorazepam- as-needed only
Outpatient detox with carbamazepine
Inpatient admission for detox with gabapentin
Inpatient admission for detox with chlordiazepoxide
Outpatient detox with disulfiram

## Slide 32
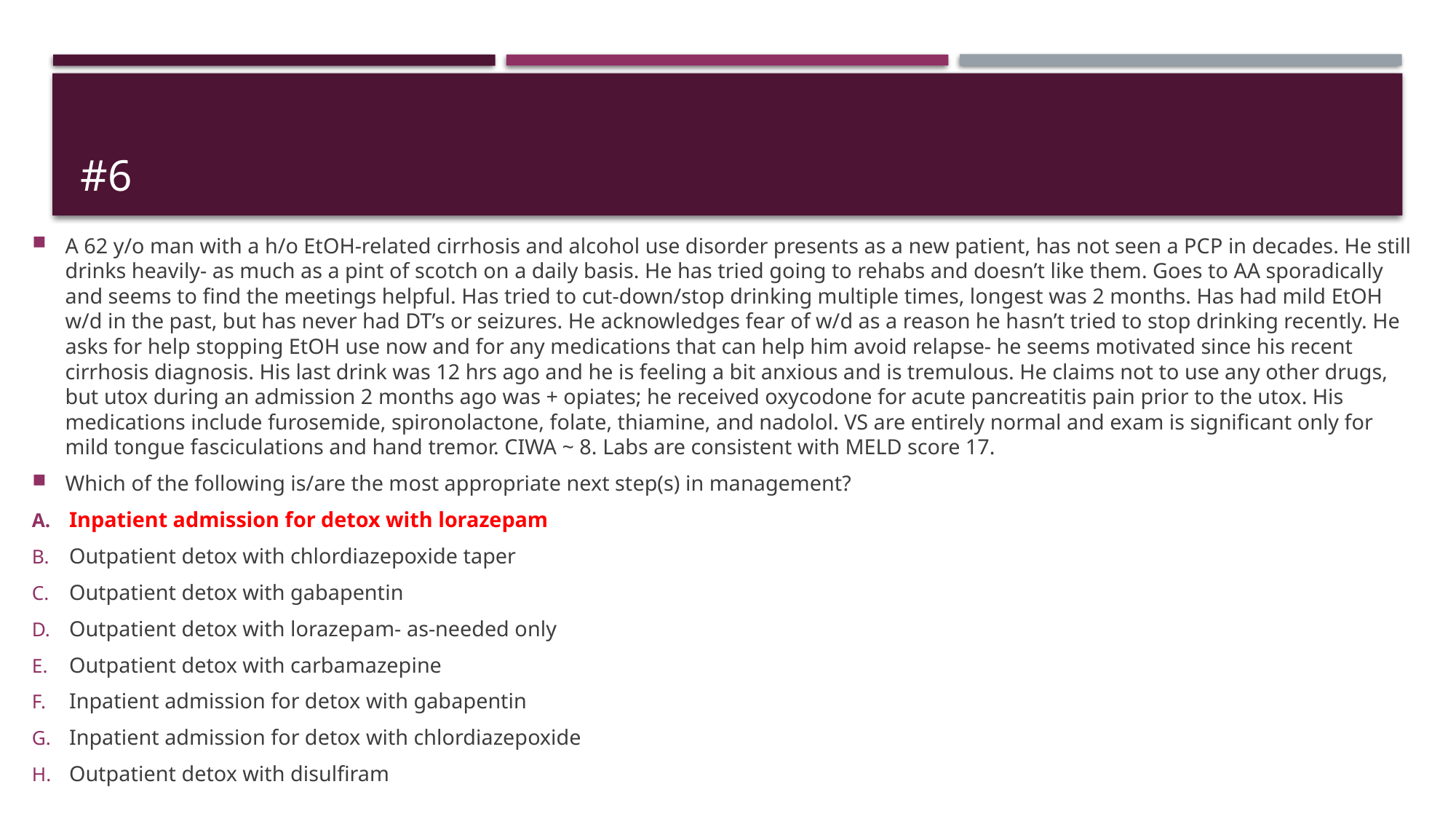

# #6
A 62 y/o man with a h/o EtOH-related cirrhosis and alcohol use disorder presents as a new patient, has not seen a PCP in decades. He still drinks heavily- as much as a pint of scotch on a daily basis. He has tried going to rehabs and doesn’t like them. Goes to AA sporadically and seems to find the meetings helpful. Has tried to cut-down/stop drinking multiple times, longest was 2 months. Has had mild EtOH w/d in the past, but has never had DT’s or seizures. He acknowledges fear of w/d as a reason he hasn’t tried to stop drinking recently. He asks for help stopping EtOH use now and for any medications that can help him avoid relapse- he seems motivated since his recent cirrhosis diagnosis. His last drink was 12 hrs ago and he is feeling a bit anxious and is tremulous. He claims not to use any other drugs, but utox during an admission 2 months ago was + opiates; he received oxycodone for acute pancreatitis pain prior to the utox. His medications include furosemide, spironolactone, folate, thiamine, and nadolol. VS are entirely normal and exam is significant only for mild tongue fasciculations and hand tremor. CIWA ~ 8. Labs are consistent with MELD score 17.
Which of the following is/are the most appropriate next step(s) in management?
Inpatient admission for detox with lorazepam
Outpatient detox with chlordiazepoxide taper
Outpatient detox with gabapentin
Outpatient detox with lorazepam- as-needed only
Outpatient detox with carbamazepine
Inpatient admission for detox with gabapentin
Inpatient admission for detox with chlordiazepoxide
Outpatient detox with disulfiram

## Slide 33
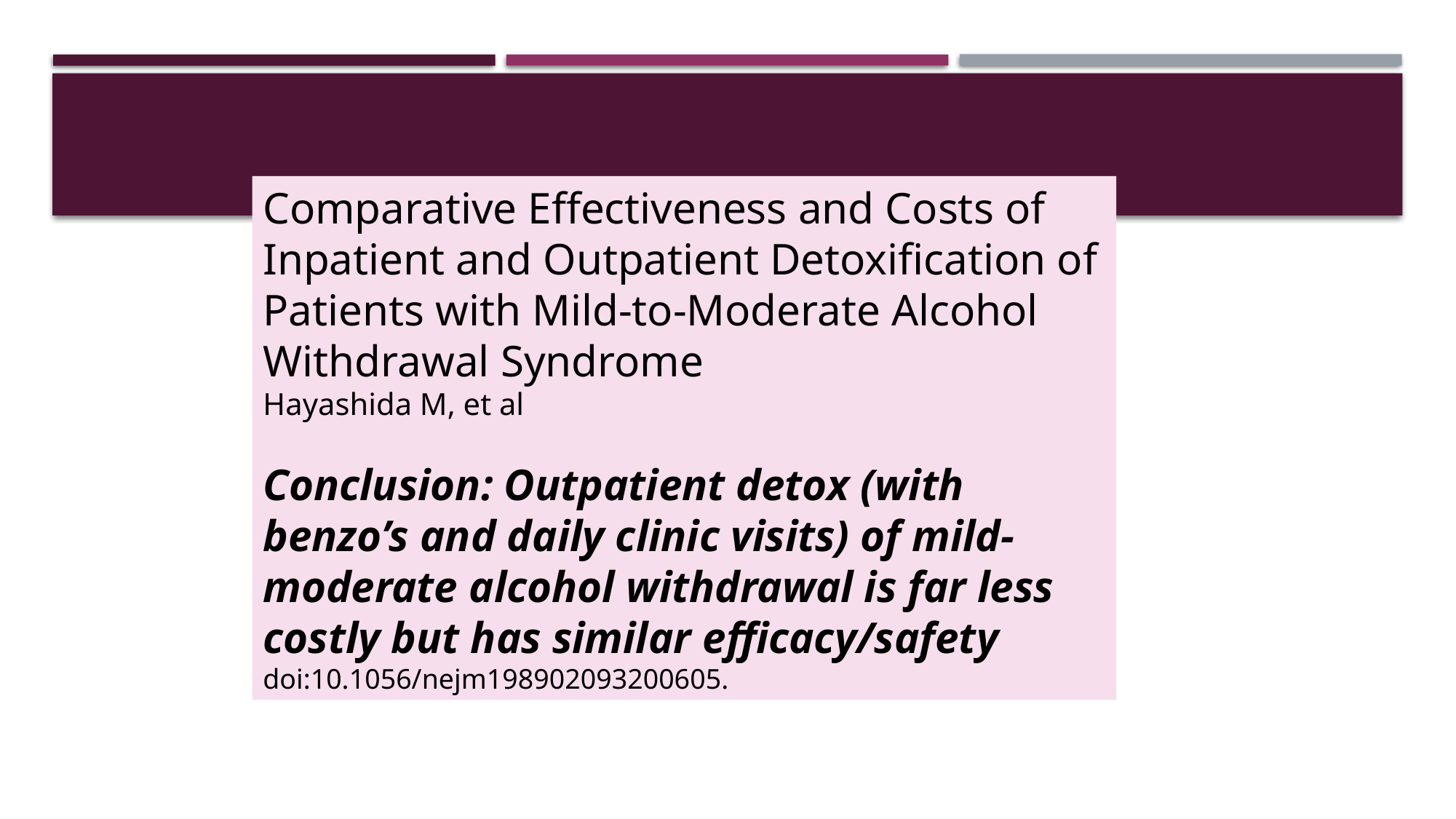

#
Comparative Effectiveness and Costs of Inpatient and Outpatient Detoxification of Patients with Mild-to-Moderate Alcohol Withdrawal Syndrome
Hayashida M, et al
Conclusion: Outpatient detox (with benzo’s and daily clinic visits) of mild-moderate alcohol withdrawal is far less costly but has similar efficacy/safety
doi:10.1056/nejm198902093200605.

## Slide 34
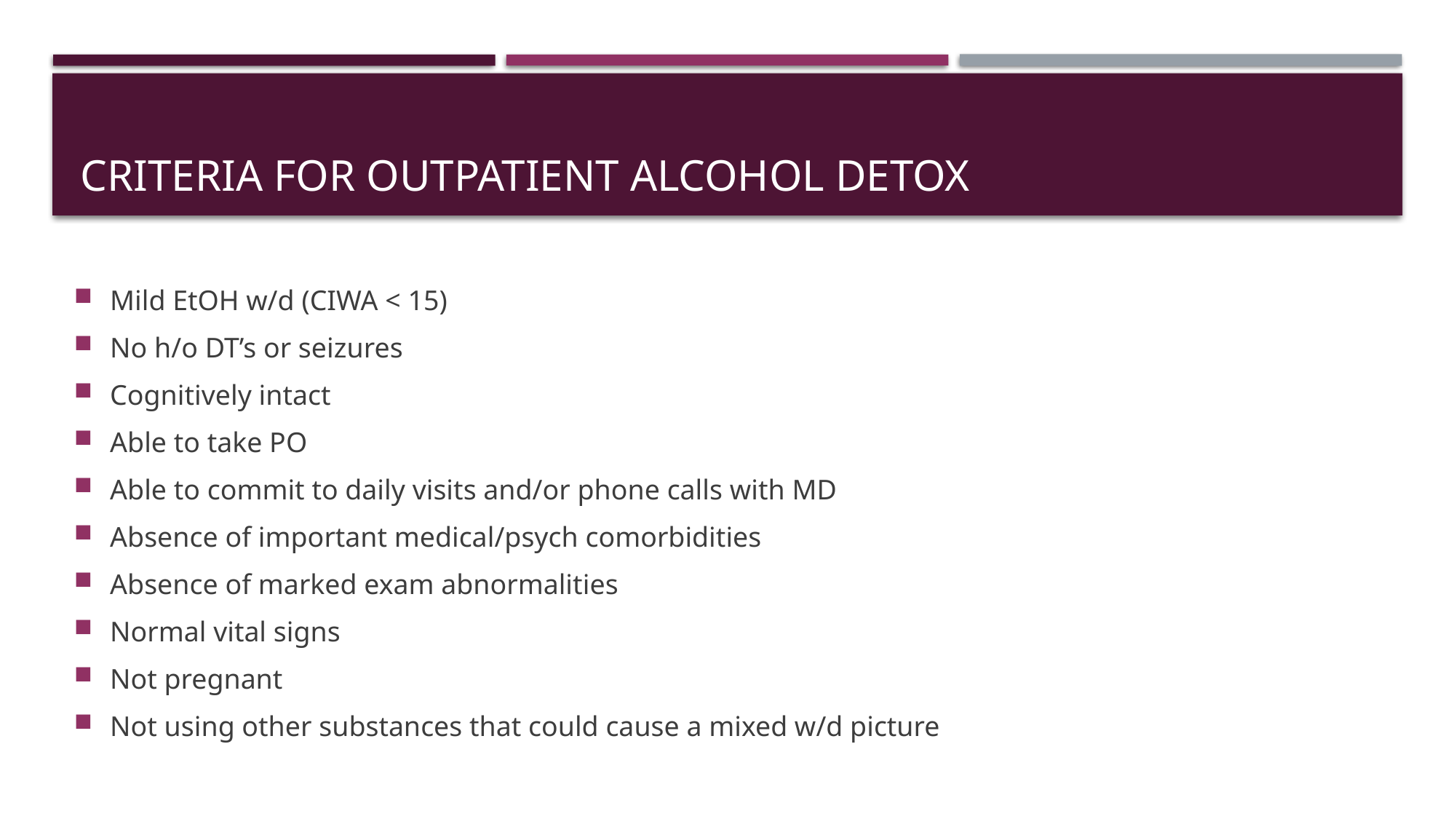

# Criteria for outpatient alcohol detox
Mild EtOH w/d (CIWA < 15)
No h/o DT’s or seizures
Cognitively intact
Able to take PO
Able to commit to daily visits and/or phone calls with MD
Absence of important medical/psych comorbidities
Absence of marked exam abnormalities
Normal vital signs
Not pregnant
Not using other substances that could cause a mixed w/d picture

## Slide 35
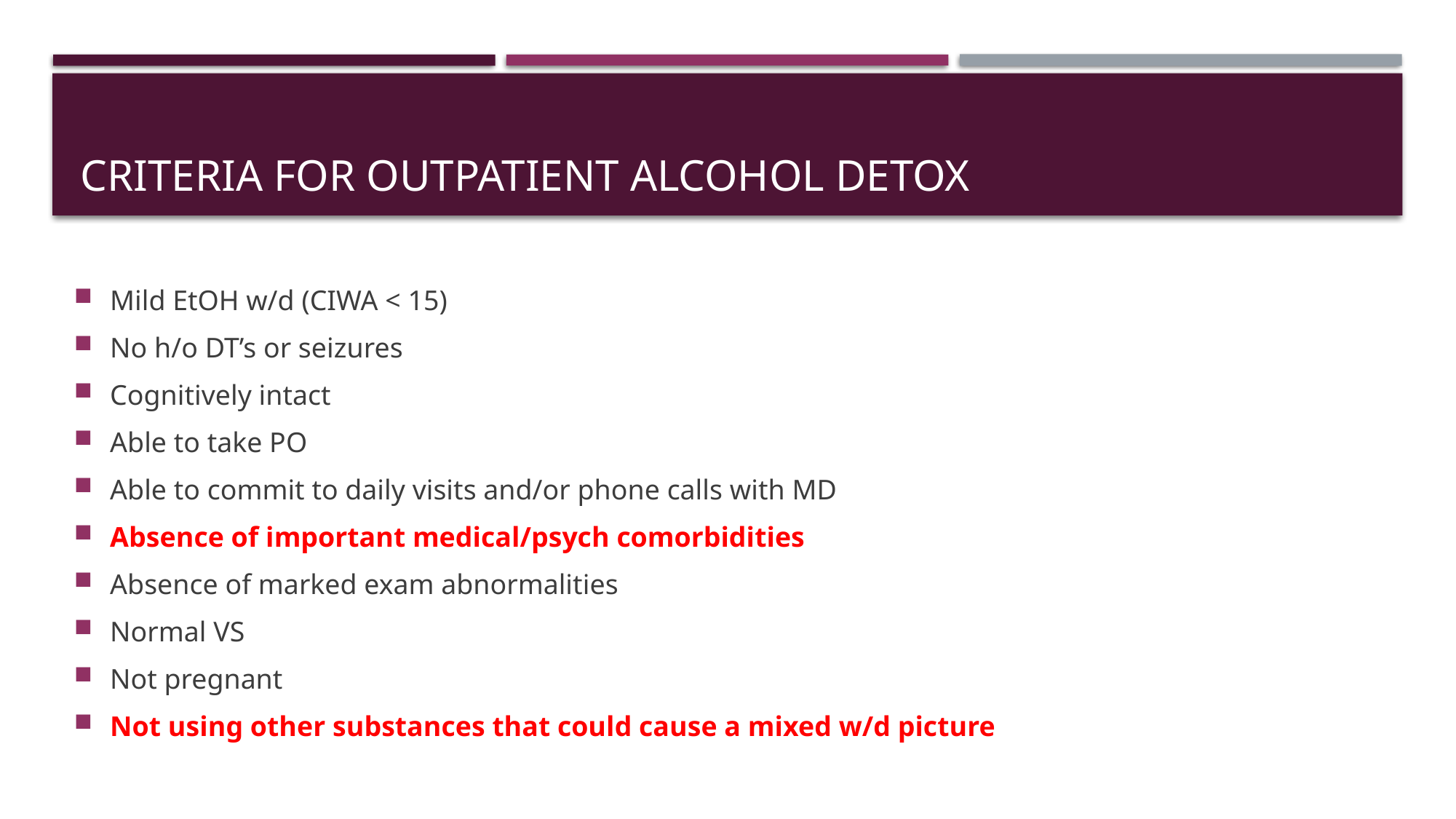

# Criteria for outpatient alcohol detox
Mild EtOH w/d (CIWA < 15)
No h/o DT’s or seizures
Cognitively intact
Able to take PO
Able to commit to daily visits and/or phone calls with MD
Absence of important medical/psych comorbidities
Absence of marked exam abnormalities
Normal VS
Not pregnant
Not using other substances that could cause a mixed w/d picture

## Slide 36
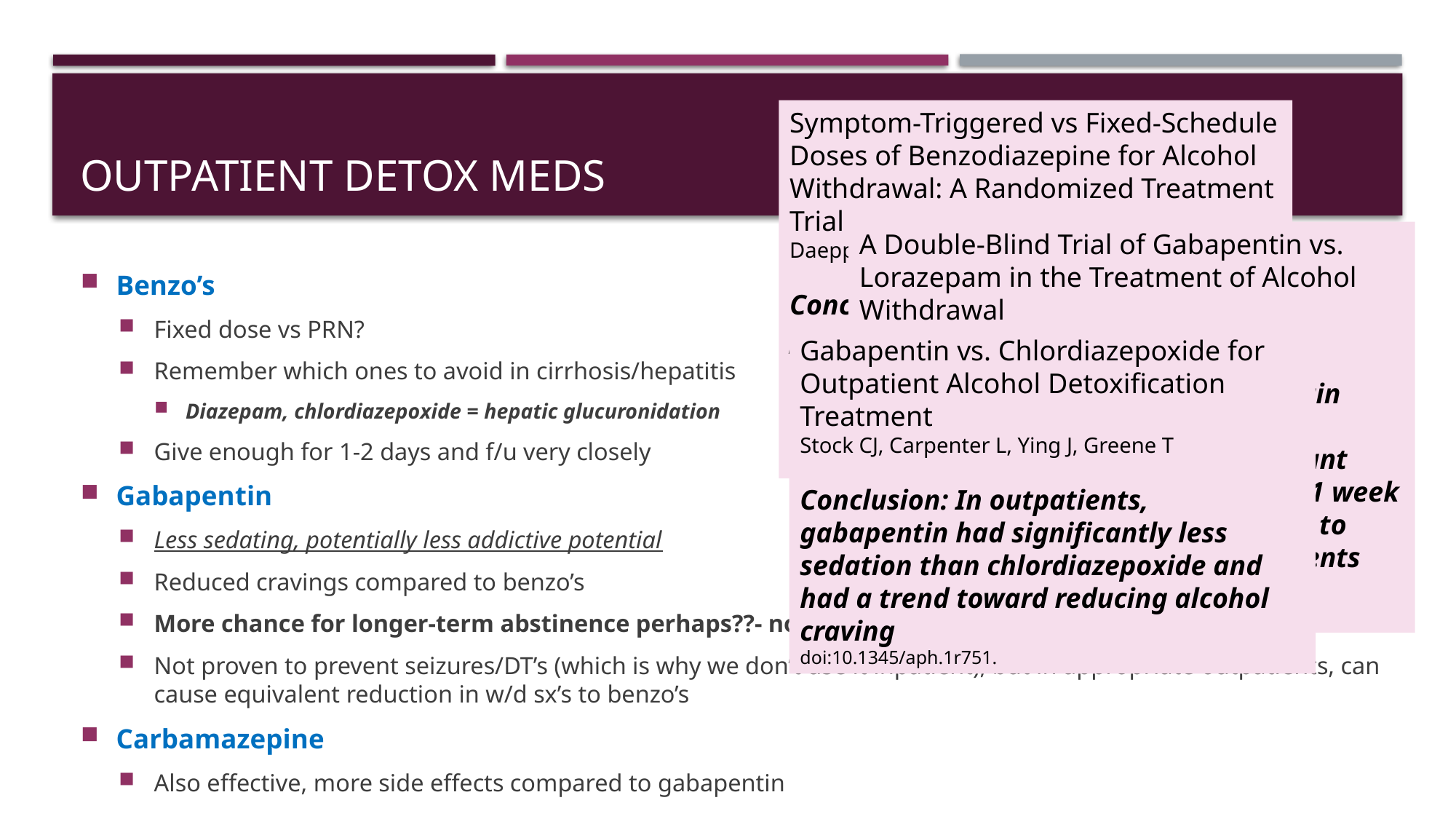

# Outpatient detox meds
Symptom-Triggered vs Fixed-Schedule Doses of Benzodiazepine for Alcohol Withdrawal: A Randomized Treatment Trial
Daeppen JB, et al
Conclusion: Symptom-triggered benzo protocols are safe, comfortable, and associated with decreased quantities of benzo usage and treatment duration
doi:10.1001/archinte.162.10.1117.
A Double-Blind Trial of Gabapentin vs. Lorazepam in the Treatment of Alcohol Withdrawal
Myrick H, et al
Conclusion: Higher dosed gabapentin effectively diminished withdrawal symptoms and led to more significant reductions in alcohol usage within 1 week of the treatment course (compared to lorazepam)- study done on outpatients only
doi:10.1111/j.1530-0277.2009.00986.x.
Benzo’s
Fixed dose vs PRN?
Remember which ones to avoid in cirrhosis/hepatitis
Diazepam, chlordiazepoxide = hepatic glucuronidation
Give enough for 1-2 days and f/u very closely
Gabapentin
Less sedating, potentially less addictive potential
Reduced cravings compared to benzo’s
More chance for longer-term abstinence perhaps??- not totally proven
Not proven to prevent seizures/DT’s (which is why we don’t use it inpatient), but in appropriate outpatients, can cause equivalent reduction in w/d sx’s to benzo’s
Carbamazepine
Also effective, more side effects compared to gabapentin
Gabapentin vs. Chlordiazepoxide for Outpatient Alcohol Detoxification Treatment
Stock CJ, Carpenter L, Ying J, Greene T
Conclusion: In outpatients, gabapentin had significantly less sedation than chlordiazepoxide and had a trend toward reducing alcohol craving
doi:10.1345/aph.1r751.

## Slide 37
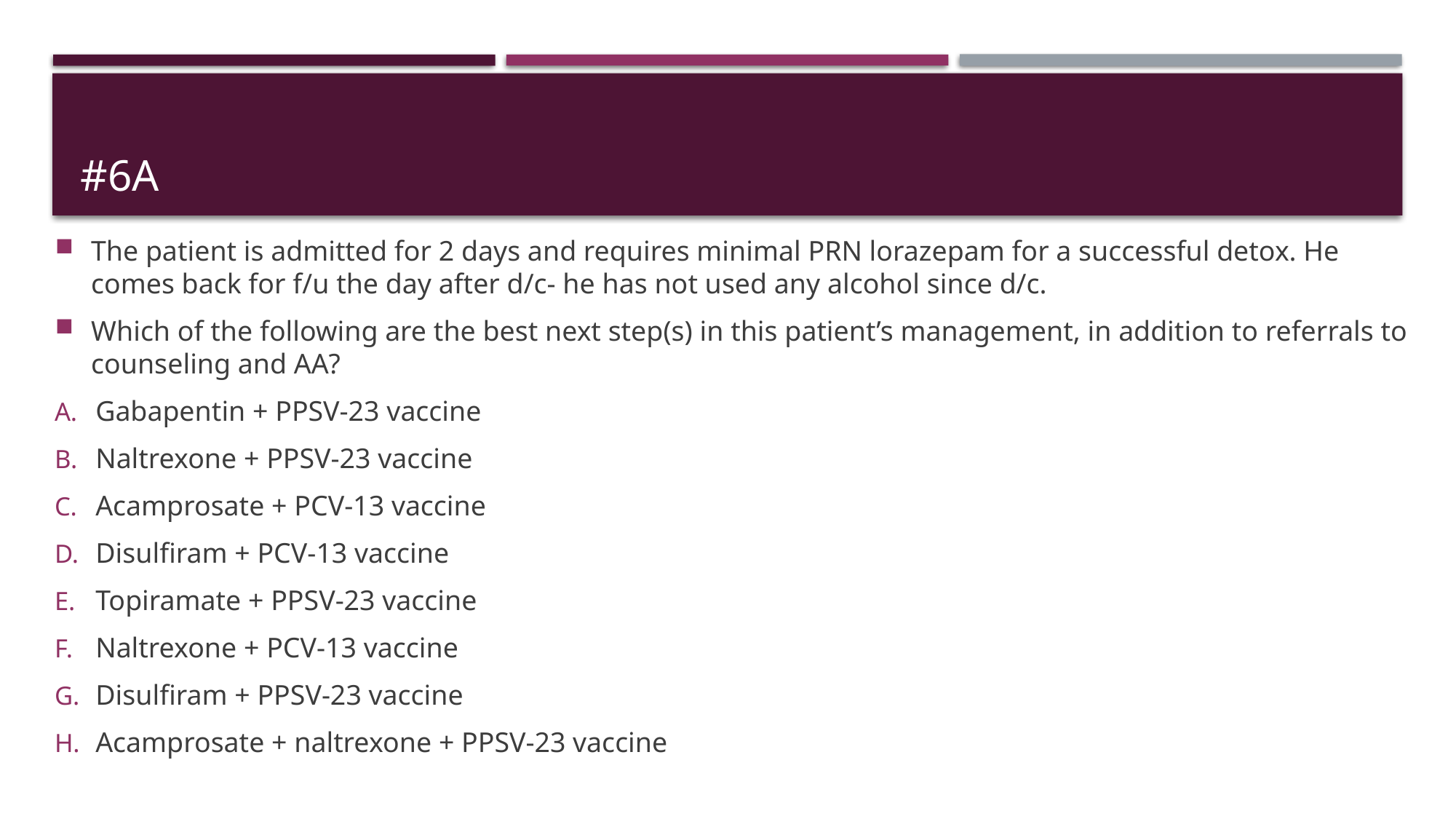

# #6A
The patient is admitted for 2 days and requires minimal PRN lorazepam for a successful detox. He comes back for f/u the day after d/c- he has not used any alcohol since d/c.
Which of the following are the best next step(s) in this patient’s management, in addition to referrals to counseling and AA?
Gabapentin + PPSV-23 vaccine
Naltrexone + PPSV-23 vaccine
Acamprosate + PCV-13 vaccine
Disulfiram + PCV-13 vaccine
Topiramate + PPSV-23 vaccine
Naltrexone + PCV-13 vaccine
Disulfiram + PPSV-23 vaccine
Acamprosate + naltrexone + PPSV-23 vaccine

## Slide 38
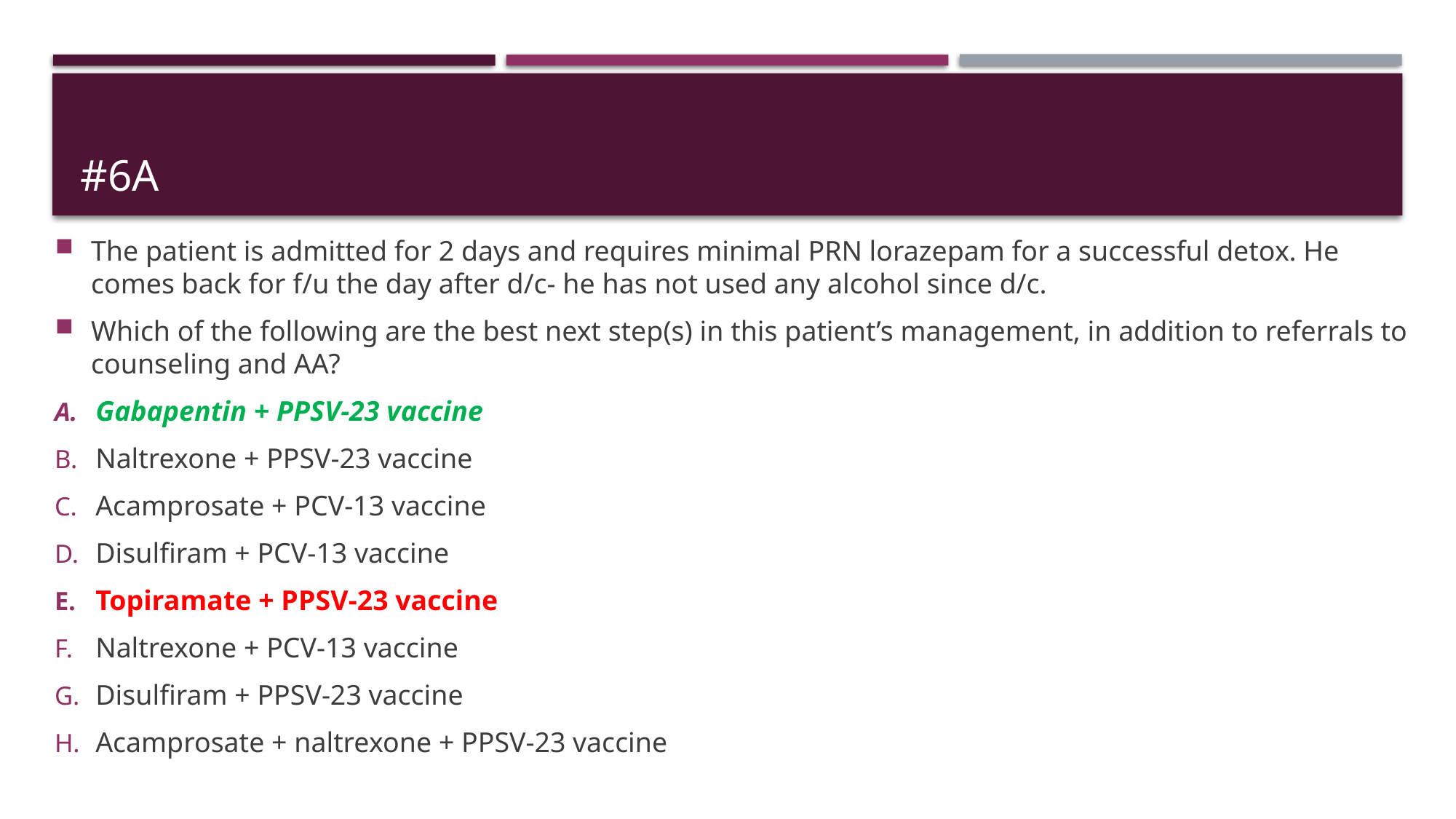

# #6A
The patient is admitted for 2 days and requires minimal PRN lorazepam for a successful detox. He comes back for f/u the day after d/c- he has not used any alcohol since d/c.
Which of the following are the best next step(s) in this patient’s management, in addition to referrals to counseling and AA?
Gabapentin + PPSV-23 vaccine
Naltrexone + PPSV-23 vaccine
Acamprosate + PCV-13 vaccine
Disulfiram + PCV-13 vaccine
Topiramate + PPSV-23 vaccine
Naltrexone + PCV-13 vaccine
Disulfiram + PPSV-23 vaccine
Acamprosate + naltrexone + PPSV-23 vaccine

## Slide 39
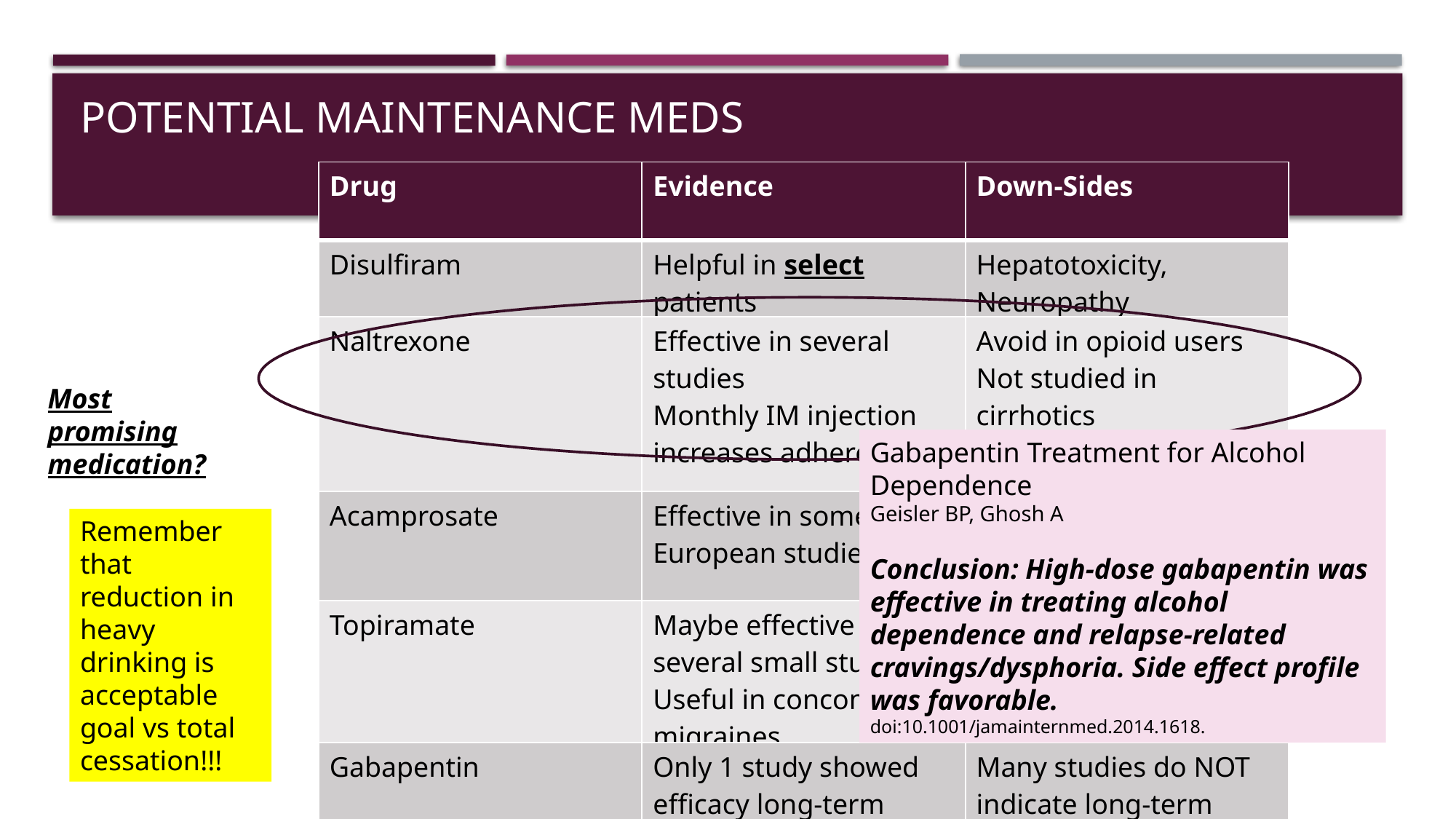

# Potential maintenance meds
| Drug | Evidence | Down-Sides |
| --- | --- | --- |
| Disulfiram | Helpful in select patients | Hepatotoxicity, Neuropathy |
| Naltrexone | Effective in several studies Monthly IM injection increases adherence | Avoid in opioid users Not studied in cirrhotics Can’t use in acute liver failure |
| Acamprosate | Effective in some European studies | Limited supportive data in USA Can’t use in GFR < 30 |
| Topiramate | Maybe effective in several small studies Useful in concomitant migraines | Side effects common |
| Gabapentin | Only 1 study showed efficacy long-term Well tolerated | Many studies do NOT indicate long-term efficacy |
Most promising medication?
Gabapentin Treatment for Alcohol Dependence
Geisler BP, Ghosh A
Conclusion: High-dose gabapentin was effective in treating alcohol dependence and relapse-related cravings/dysphoria. Side effect profile was favorable.
doi:10.1001/jamainternmed.2014.1618.
Remember that reduction in heavy drinking is acceptable goal vs total cessation!!!

## Slide 40
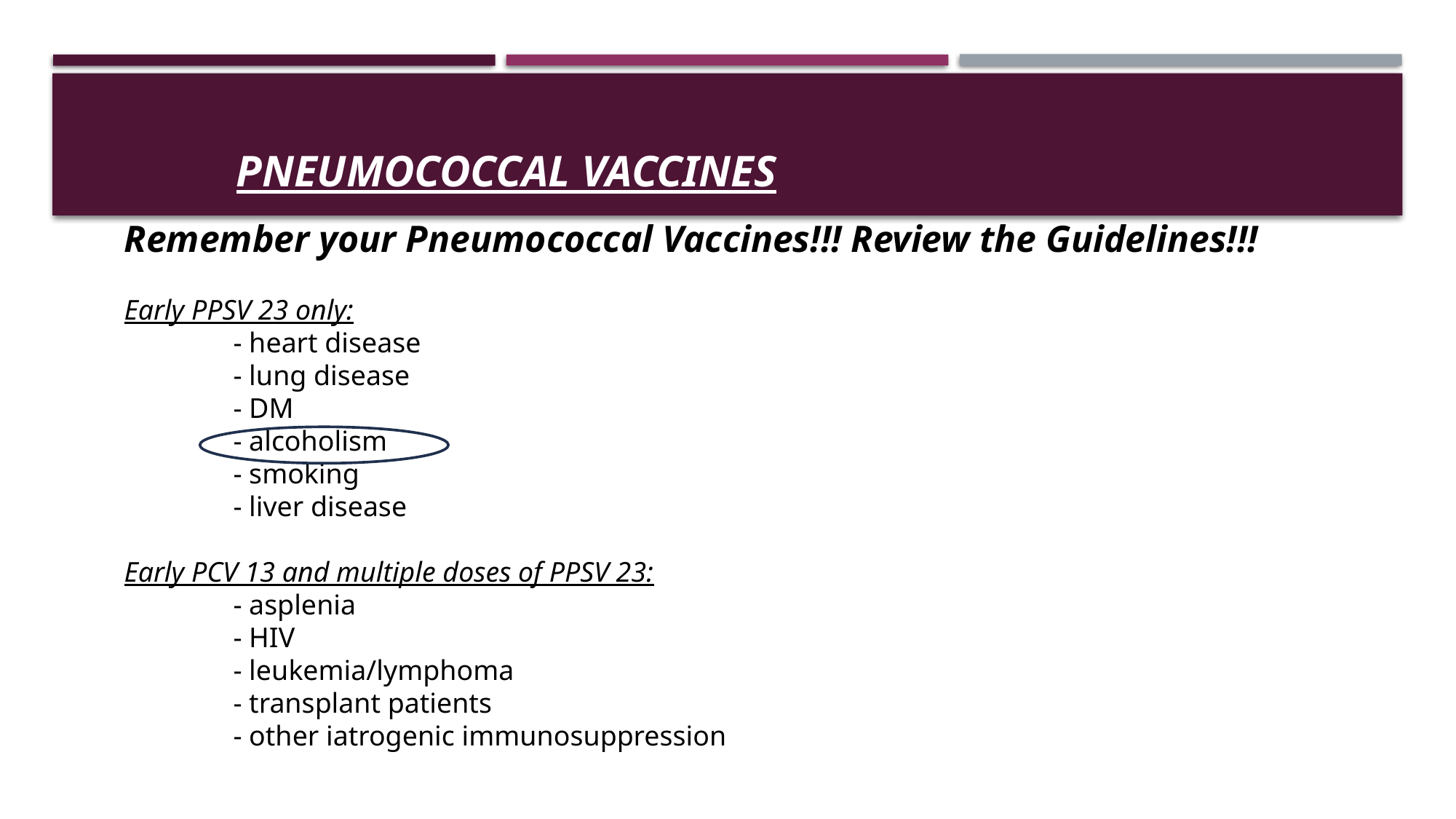

# Pneumococcal Vaccines
Remember your Pneumococcal Vaccines!!! Review the Guidelines!!!
Early PPSV 23 only:
	- heart disease
	- lung disease
	- DM
	- alcoholism
	- smoking
	- liver disease
Early PCV 13 and multiple doses of PPSV 23:
	- asplenia
	- HIV
	- leukemia/lymphoma
	- transplant patients
	- other iatrogenic immunosuppression

## Slide 41
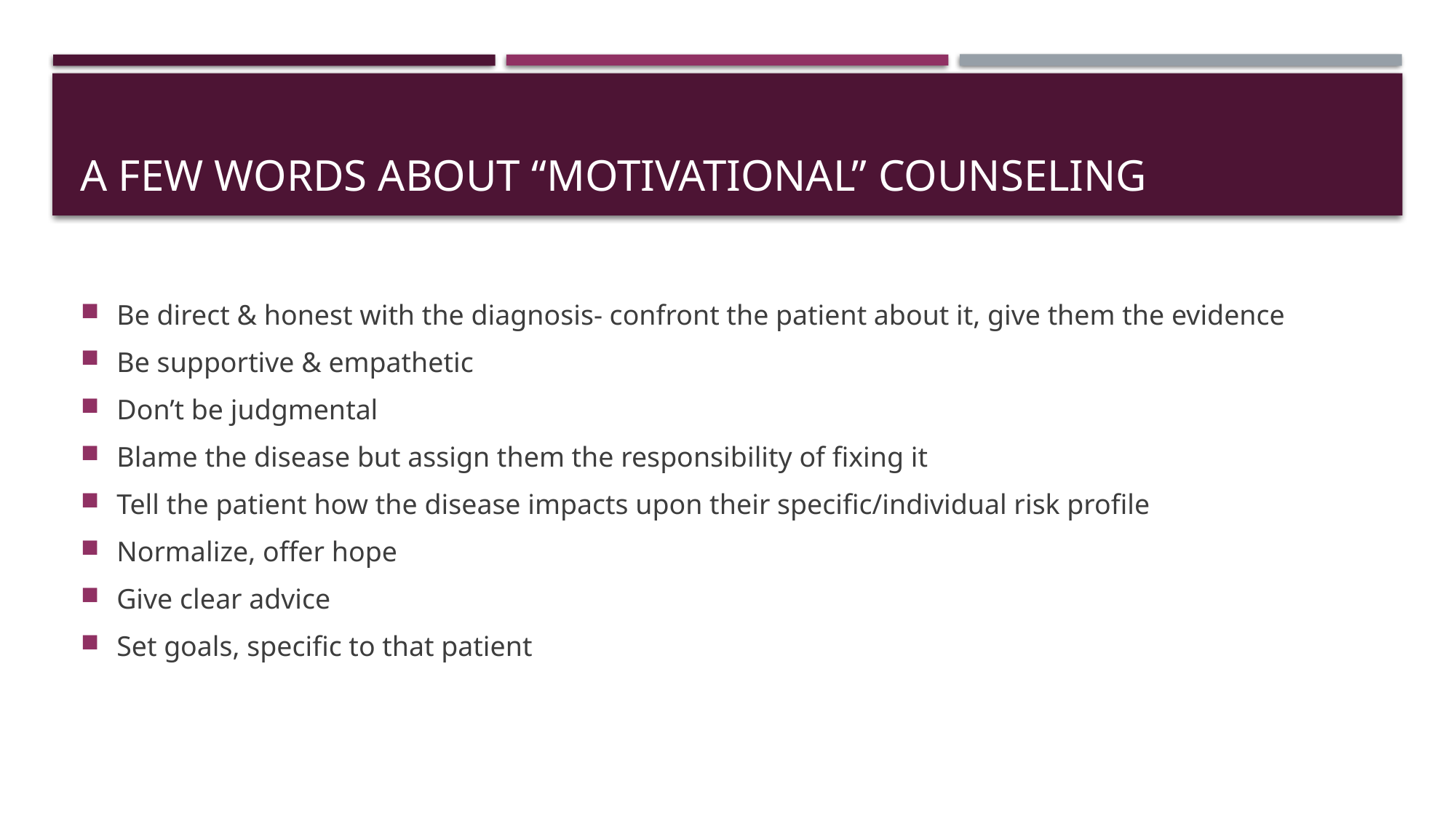

# A Few Words about “motivational” counseling
Be direct & honest with the diagnosis- confront the patient about it, give them the evidence
Be supportive & empathetic
Don’t be judgmental
Blame the disease but assign them the responsibility of fixing it
Tell the patient how the disease impacts upon their specific/individual risk profile
Normalize, offer hope
Give clear advice
Set goals, specific to that patient

## Slide 42
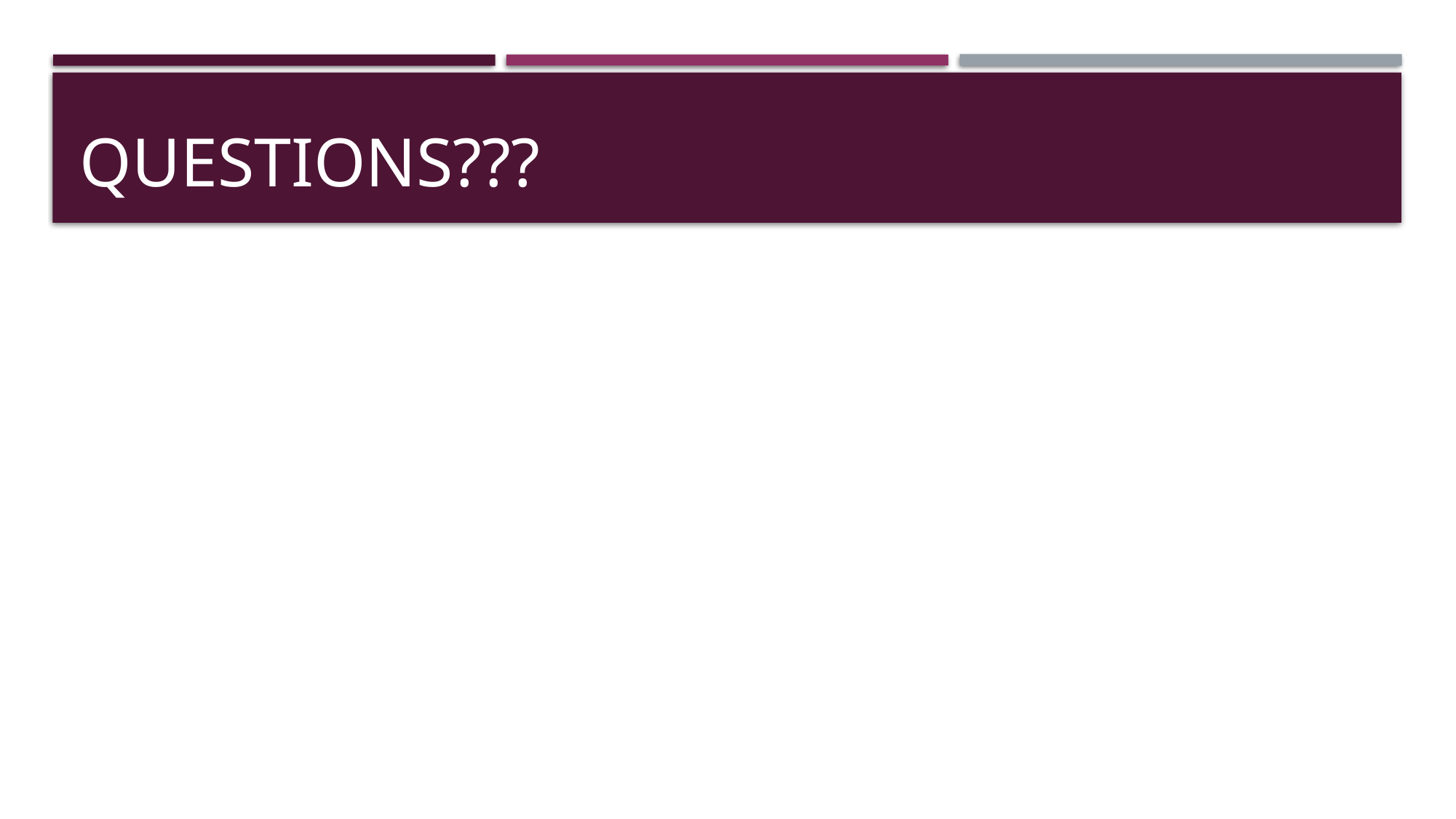

# Questions???

## Slide 43
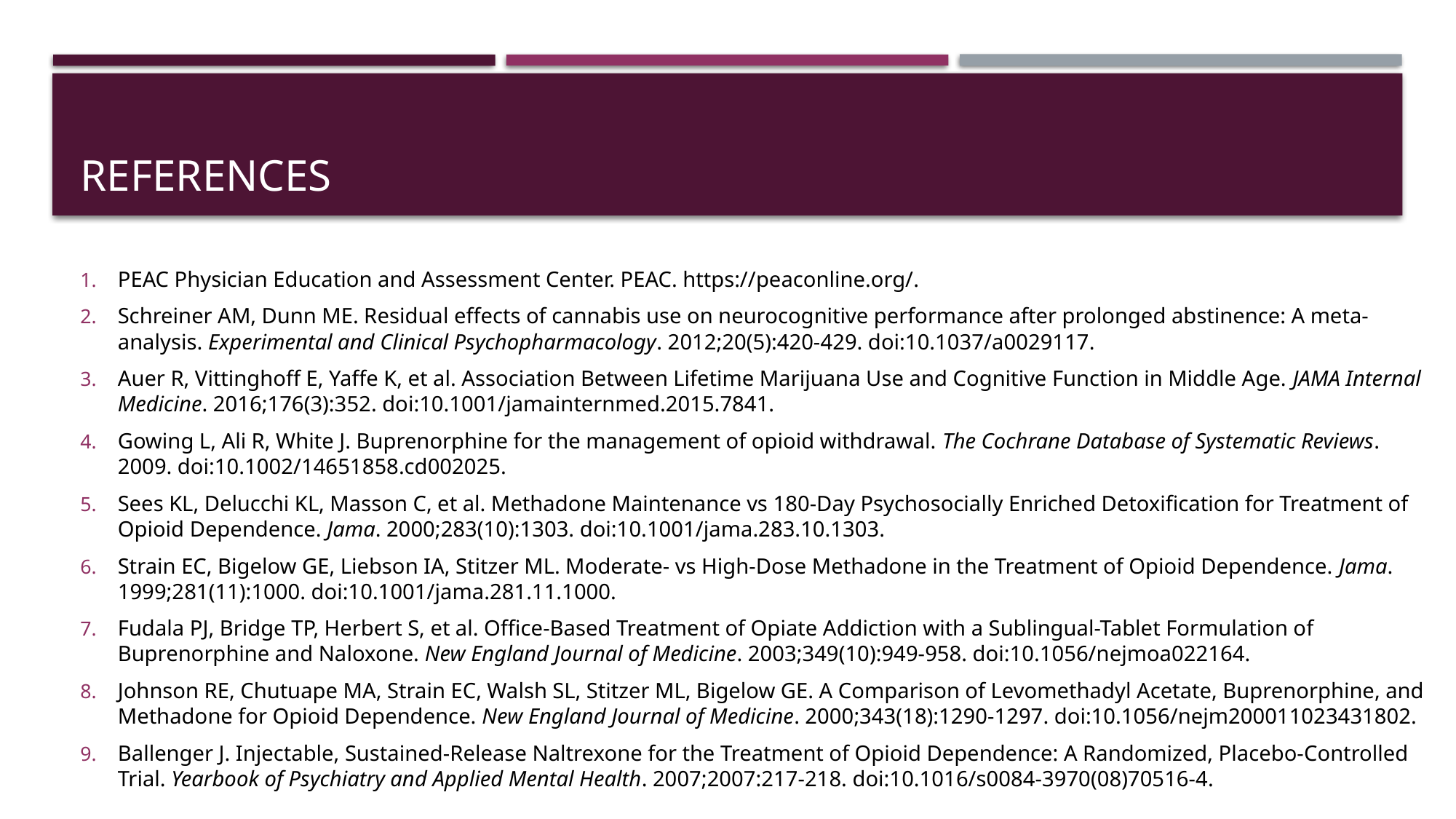

# References
PEAC Physician Education and Assessment Center. PEAC. https://peaconline.org/.
Schreiner AM, Dunn ME. Residual effects of cannabis use on neurocognitive performance after prolonged abstinence: A meta-analysis. Experimental and Clinical Psychopharmacology. 2012;20(5):420-429. doi:10.1037/a0029117.
Auer R, Vittinghoff E, Yaffe K, et al. Association Between Lifetime Marijuana Use and Cognitive Function in Middle Age. JAMA Internal Medicine. 2016;176(3):352. doi:10.1001/jamainternmed.2015.7841.
Gowing L, Ali R, White J. Buprenorphine for the management of opioid withdrawal. The Cochrane Database of Systematic Reviews. 2009. doi:10.1002/14651858.cd002025.
Sees KL, Delucchi KL, Masson C, et al. Methadone Maintenance vs 180-Day Psychosocially Enriched Detoxification for Treatment of Opioid Dependence. Jama. 2000;283(10):1303. doi:10.1001/jama.283.10.1303.
Strain EC, Bigelow GE, Liebson IA, Stitzer ML. Moderate- vs High-Dose Methadone in the Treatment of Opioid Dependence. Jama. 1999;281(11):1000. doi:10.1001/jama.281.11.1000.
Fudala PJ, Bridge TP, Herbert S, et al. Office-Based Treatment of Opiate Addiction with a Sublingual-Tablet Formulation of Buprenorphine and Naloxone. New England Journal of Medicine. 2003;349(10):949-958. doi:10.1056/nejmoa022164.
Johnson RE, Chutuape MA, Strain EC, Walsh SL, Stitzer ML, Bigelow GE. A Comparison of Levomethadyl Acetate, Buprenorphine, and Methadone for Opioid Dependence. New England Journal of Medicine. 2000;343(18):1290-1297. doi:10.1056/nejm200011023431802.
Ballenger J. Injectable, Sustained-Release Naltrexone for the Treatment of Opioid Dependence: A Randomized, Placebo-Controlled Trial. Yearbook of Psychiatry and Applied Mental Health. 2007;2007:217-218. doi:10.1016/s0084-3970(08)70516-4.

## Slide 44
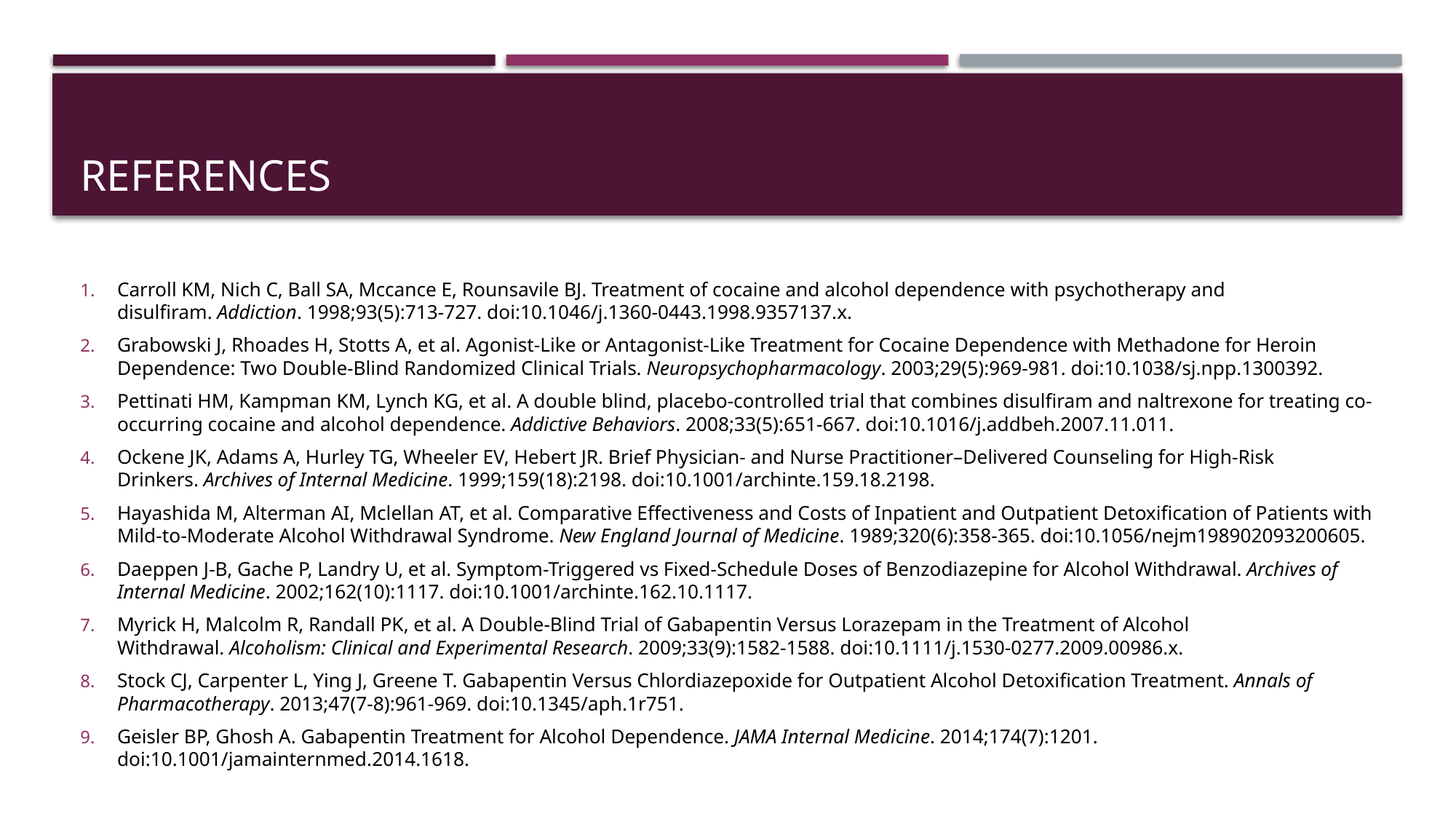

# References
Carroll KM, Nich C, Ball SA, Mccance E, Rounsavile BJ. Treatment of cocaine and alcohol dependence with psychotherapy and disulfiram. Addiction. 1998;93(5):713-727. doi:10.1046/j.1360-0443.1998.9357137.x.
Grabowski J, Rhoades H, Stotts A, et al. Agonist-Like or Antagonist-Like Treatment for Cocaine Dependence with Methadone for Heroin Dependence: Two Double-Blind Randomized Clinical Trials. Neuropsychopharmacology. 2003;29(5):969-981. doi:10.1038/sj.npp.1300392.
Pettinati HM, Kampman KM, Lynch KG, et al. A double blind, placebo-controlled trial that combines disulfiram and naltrexone for treating co-occurring cocaine and alcohol dependence. Addictive Behaviors. 2008;33(5):651-667. doi:10.1016/j.addbeh.2007.11.011.
Ockene JK, Adams A, Hurley TG, Wheeler EV, Hebert JR. Brief Physician- and Nurse Practitioner–Delivered Counseling for High-Risk Drinkers. Archives of Internal Medicine. 1999;159(18):2198. doi:10.1001/archinte.159.18.2198.
Hayashida M, Alterman AI, Mclellan AT, et al. Comparative Effectiveness and Costs of Inpatient and Outpatient Detoxification of Patients with Mild-to-Moderate Alcohol Withdrawal Syndrome. New England Journal of Medicine. 1989;320(6):358-365. doi:10.1056/nejm198902093200605.
Daeppen J-B, Gache P, Landry U, et al. Symptom-Triggered vs Fixed-Schedule Doses of Benzodiazepine for Alcohol Withdrawal. Archives of Internal Medicine. 2002;162(10):1117. doi:10.1001/archinte.162.10.1117.
Myrick H, Malcolm R, Randall PK, et al. A Double-Blind Trial of Gabapentin Versus Lorazepam in the Treatment of Alcohol Withdrawal. Alcoholism: Clinical and Experimental Research. 2009;33(9):1582-1588. doi:10.1111/j.1530-0277.2009.00986.x.
Stock CJ, Carpenter L, Ying J, Greene T. Gabapentin Versus Chlordiazepoxide for Outpatient Alcohol Detoxification Treatment. Annals of Pharmacotherapy. 2013;47(7-8):961-969. doi:10.1345/aph.1r751.
Geisler BP, Ghosh A. Gabapentin Treatment for Alcohol Dependence. JAMA Internal Medicine. 2014;174(7):1201. doi:10.1001/jamainternmed.2014.1618.
