# Supplementary material for: A Novel Team-Based Learning Approach for an Internal Medicine Residency: Medication-Assisted Treatments for Substance Use Disorders
Source: MedEdPORTAL. 2021 Feb 1;17:11085. doi: 10.15766/mep_2374-8265.11085 (PMC7852341; doi:10.15766/mep_2374-8265.11085)
Supplement: Supplementary file 1 — iRAT without Answers.docxiRAT with Answers.docxTeam Application Exercise.pptxFacilitators Guide to the Team App Exercise.docxResident Evaluation of the TBL Activity.docx [file mep_2374-8265.11085-s001.zip › D. Facilitators Guide to the Team App Exercise.docx]

**ATTENTION, STUDENTS:** If you are accessing this material BEFORE it is used in your course, please do NOT read this document prior to the class session. An answer key is included in this module, which is designed to lead you through a learning experience that reinforces your knowledge of the content. Early review or dissemination of this material to others will diminish the learning opportunity and be considered academic misconduct.

**Appendix D: Facilitator’s Guide for the Addiction TBL Application Exercise**

Remember that ALL questions could have more than one correct answer- this helps stimulate discussion and debate amongst the learners!

**Question 1:**

Educational Objective- Familiarize the resident with the appropriate terminology and criteria for a substance use disorder, as well as illustrate potential health risks with marijuana usage.

A- incorrect- wrong diagnosis terminology, although the counseling piece is correct

B- incorrect- wrong diagnosis terminology- and marijuana use has NO proven permanent neurocognitive deficits for adults after stopped- see following slides

C- incorrect- wrong diagnosis terminology- although psychosis is a potential side effect of marijuana

D- incorrect- correct diagnosis- but again there are no known permanent cognitive deficits with marijuana

**E- Correct!**

F- incorrect- the patient does meet the criteria for a diagnosis of cannabis use disorder- see next slides

G- incorrect- same reasoning as F

H- incorrect- same reasoning as F

Most groups got the correct answer here, but several selected A and H because they were not clear on the correct terminology and disorder criteria.

----------------------------------------------

**Question 2:**

Educational Objective- Identify an opioid use disorder and acceptable options for medication-assisted treatment (MAT).

A- incorrect- detoxification programs have not been shown to have long-term success, and in fact can be dangerous to some patients as they have a higher risk of overdose when relapsing after > 7 days of abstinence; head-to-head, medication-assisted treatments have always won

**B- Acceptable option!**

C- incorrect- methadone can only be prescribed in a government-approved methadone clinic

**D- Acceptable option- same efficacy as methadone but may be preferred due to an improved safety profile- more on this later…**

E- incorrect- except in cirrhosis patients, naloxone is always included in buprenorphine products to reduce divergence/misuse of the medication

F- incorrect- useful as an adjunct to detox/abstinence, but cannot be administered until at least 7 days (preferably 10 days) of opioid washout have been attained to prevent precipitating withdrawal

**G- Correct! Always a good idea if the patient is amenable**

H- incorrect- inferior to methadone and buprenorphine

D&G were popular answers for this question. Not every group knew that methadone had similar efficacy to buprenorphine, so B was not always chosen. Several groups chose A, primarily because of lack of knowledge of what those detox programs do and their general lack of efficacy compared to MAT.

---------------------------------------------------

**Question 2A: Builds off of Question 2- not meant to be an in-depth group/TBL discussion, just meant to illustrate a quick learning point**

Educational Objective- Illustrate that insurance/financial status has an impact on decisions regarding MAT options.

Buprenorphine is much more expensive per month than methadone for patients that pay out-of-pocket. Generic versions of buprenorphine tabs/films have become more affordable recently, but still are much more expensive in lump sums of money every month than going to a methadone clinic. In addition, methadone programs will often provide supportive and counseling services that a patient may not be able to obtain from a mental health clinic when they don’t have insurance.

B is thus the best option and most groups consistently made this selection.

-------------------------------------------

**Question 3:**

Educational Objective- Educate residents on the pharmacology of MAT options for opioid use disorder, showing how one solution may not be the best choice for every patient depending on their circumstances.

A- incorrect, as per question #2

**B- Still acceptable, and actually maybe preferred here because of the opportunity to harness methadone’s full opioid agonist activity to improve pain control**

**C- Correct- NSAID’s are an acceptable adjunct for pain control for bony metastases and may help reduce quantity of opioid required**

*D- The jury is still out on buprenorphine for this patient- arguments can be made for and against this option, so we have counted this as a neutral answer. On the one hand, buprenorphine is not a great pain control agent and due to its partial agonist activity, it would impede the use of additional prescribed opioids for pain control. On the other hand, arguments can be made that it is still safer than methadone and can be used for pain control in some patients.*

E- incorrect- rapidly tapering off buprenorphine or methadone is a method of detox and is proven to NOT be effective long-term, especially in someone likely to require long-term opioids for pain control

F- incorrect- same reasoning as #2

G- incorrect- same reasoning as #2

**H- Correct!- narcan kit should ALWAYS be given to anyone for whom you prescribe or refer for long-term narcotics, whether that is buprenorphine, methadone, or opioid pills- has the potential to save other people’s lives also!**

Most groups selected D&H for this question, many also chose B. C was rarely chosen, due to perception that NSAID’s would not contribute to pain control in a patient using so much opioid. This was an opportunity to further teach harm reduction principles- i.e. that *some* reduction in opioid usage is still an acceptable goal and can be achieved with NSAID’s for bone pain.

---------------------------------------------------

**Question 4:**

Educational Objective- Introduce residents to potential treatment options for cocaine use disorder.

**A- Correct- Rehab facilities and counseling are two of our very few tools in treating cocaine use disorder**

**B- Correct- NA can be a resource for many different substance use disorders, not just opioids**

C- incorrect- no proven usefulness in cocaine use disorder, but could be helpful for co-occurring mood disorders

D- incorrect- no proven efficacy in cocaine use disorder

E- incorrect- no proven efficacy in cocaine use disorder

F- incorrect- should remain on some beta blocker for CAD/HTN

*G- maybe correct?- the risk of unopposed alpha activity with cocaine use (while someone is getting a beta blocker) is still theoretical and not proven to carry any weight clinically- nevertheless, if given the option to switch to a less cardio-selective beta blocker such as carvedilol, it may be safer to do so*

H- incorrect, for now- but MAT for cocaine use disorder is being studied and may soon be a correct option!

Most groups selected A, many were not aware that Narcotics Anonymous (B) could also accommodate cocaine and other psychotropic drug addictions. G was controversial as expected, as noted above.

-----------------------------------------
**Question 5:**

Educational Objective- Recognizing unhealthy alcohol use, diagnosing a disorder, and suggesting treatment options.

A- incorrect- has several criteria for a disorder, but AA/counseling are acceptable treatment options

B- incorrect- same as A, and disulfiram is only for select patients that are highly motivated

C- incorrect- correct diagnosis but outpatient rehab for a mild disorder is not usually necessary- counseling alone would likely be helpful for now

D- incorrect- is a mild disorder, based on number of criteria met (see following slides); it may be useful to note, though, that naltrexone would be an acceptable medication choice for this patient

**E- Correct!**

F- incorrect- is a mild disorder

**G- Correct! Topiramate has potential efficacy in curbing long-term alcohol use and could be helpful for her migraines as well**

H- incorrect- wrong diagnosis again

Most groups chose E. Very few chose G because of unawareness of topiramate’s potential role in treating alcohol use disorder. Several chose D&F incorrectly.

----------------------------------------------

**Question 6:**

Educational Objective- Identify appropriate candidates for outpatient alcohol detoxification and treatment of withdrawal.

**A- Correct!- his cirrhosis places him at higher risk for DT’s and severe EtOH withdrawal- hence he needs to be admitted for detox/withdrawal- lorazepam is best drug option in the setting of his cirrhosis**

B- incorrect- not a candidate for outpatient treatment

C- incorrect- not a candidate for outpatient treatment- however, if you were to do outpatient detox with another patient, gabapentin taper regimens have been shown to be near-equally efficacious to benzodiazepines in low-risk patients

D- incorrect- not a candidate for outpatient treatment- but worth noting that PRN regimens are preferred to scheduled benzodiazepine tapers

E- incorrect- not a candidate for outpatient treatment

F- incorrect- inpatient treatment is generally with benzodiazepines- gabapentin is not proven (or well-studied) for inpatient populations and has not been shown to reduce DT’s or seizures in these high-risk patients

G- incorrect- chlordiazepoxide is less safe for cirrhotics

H- incorrect- not a candidate for outpatient treatment, and disulfiram is not a detox option

Most groups got the correct answer here, choice A. Some chose G and were unaware of the risks of chlordiazepoxide in cirrhotic patients.

---------------------------------------------

**Question 6A: Follow-up to #6**

Educational Objective- Identifying appropriate medication-assisted treatments for alcohol use disorder- and a reminder about appropriate pneumococcal vaccines in this population.

*A- maybe correct?- some evidence has been found to support the use of gabapentin or baclofen for maintaining abstinence from alcohol- but evidence is weak (short-term studies) and both drugs have abuse potential- PPSV-23 vaccine is correct though*

B- incorrect- naltrexone not studied or recommended for cirrhotics, also worth mentioning that it is contraindicated in acute hepatic failure

C- incorrect- acamprosate would be acceptable but PCV-13 vaccine is incorrect

D- incorrect- disulfiram is third-line to acamprosate and naltrexone and also not advised in cirrhosis- should only be used in highly motivated/uncomplicated patients- the vaccine is also incorrect

**E- Correct!- topiramate has shown efficacy in treating alcohol use disorder, though side effects are sometimes limiting- PPSV-23 vaccine is correct**

F- incorrect- same reasoning as B

G- incorrect- same reasoning as D

H- incorrect- combination therapy is not well-studied and naltrexone is contraindicated in cirrhotics

Several groups chose E correctly. However, this question generated much discussion. Many groups incorrectly chose B, unaware that naltrexone is contraindicated in most cirrhotic patients. Other groups incorrectly chose C because of incorrect understanding of pneumococcal vaccine indications.
