# Supplementary material for: A Novel Team-Based Learning Approach for an Internal Medicine Residency: Medication-Assisted Treatments for Substance Use Disorders
Source: MedEdPORTAL. 2021 Feb 1;17:11085. doi: 10.15766/mep_2374-8265.11085 (PMC7852341; doi:10.15766/mep_2374-8265.11085)
Supplement: Supplementary file 1 — iRAT without Answers.docxiRAT with Answers.docxTeam Application Exercise.pptxFacilitators Guide to the Team App Exercise.docxResident Evaluation of the TBL Activity.docx [file mep_2374-8265.11085-s001.zip › E. Resident Evaluation of the TBL Activity.docx]

**Evaluation- *A Novel Team-Based Learning Approach for an Internal Medicine Residency: Medication-Assisted Treatments for Substance Use Disorders***

Questions (Likert Scale):

1. The TBL increased my knowledge and understanding of the DSM-5 criteria for substance use disorders.

| Strongly Disagree | Disagree | Neutral | Agree | Strongly Agree |
| --- | --- | --- | --- | --- |

1. The TBL increased my knowledge and understanding of the negative and dangerous effects of various substances of abuse- e.g. cocaine, opioids, alcohol, cannabis.

| Strongly Disagree | Disagree | Neutral | Agree | Strongly Agree |
| --- | --- | --- | --- | --- |

1. The TBL increased my knowledge and understanding of the various options available for the treatment of opioid use disorder.

| Strongly Disagree | Disagree | Neutral | Agree | Strongly Agree |
| --- | --- | --- | --- | --- |

1. As a result of this TBL, I feel more comfortable diagnosing and counseling patients with substance use disorders.

| Strongly Disagree | Disagree | Neutral | Agree | Strongly Agree |
| --- | --- | --- | --- | --- |

1. As a result of this TBL, I am more likely to suggest prescribing buprenorphine or referring to a methadone clinic, rather than reflexively sending the patient to an addiction specialist.

| Strongly Disagree | Disagree | Neutral | Agree | Strongly Agree |
| --- | --- | --- | --- | --- |

1. As a result of this TBL, I am more likely to suggest medication-assisted therapies for my patients with alcohol use disorder (e.g. Acamprosate, naltrexone).

| Strongly Disagree | Disagree | Neutral | Agree | Strongly Agree |
| --- | --- | --- | --- | --- |

1. I feel more competent in treating patients that suffer from addiction.

| Strongly Disagree | Disagree | Neutral | Agree | Strongly Agree |
| --- | --- | --- | --- | --- |

1. The information in the TBL was relevant to my practice in primary care.

| Strongly Disagree | Disagree | Neutral | Agree | Strongly Agree |
| --- | --- | --- | --- | --- |

1. Compared to lectures in similar topics, I found the TBL to be more informative and engaging.

| Strongly Disagree | Disagree | Neutral | Agree | Strongly Agree |
| --- | --- | --- | --- | --- |

Open-Ended Question:

10) Do you have any positive comments or suggestions for improving this activity?
