# Supplementary material for: A Novel Team-Based Learning Approach for an Internal Medicine Residency: Medication-Assisted Treatments for Substance Use Disorders
Source: MedEdPORTAL. 2021 Feb 1;17:11085. doi: 10.15766/mep_2374-8265.11085 (PMC7852341; doi:10.15766/mep_2374-8265.11085)
Supplement: Supplementary file 1 — iRAT without Answers.docxiRAT with Answers.docxTeam Application Exercise.pptxFacilitators Guide to the Team App Exercise.docxResident Evaluation of the TBL Activity.docx [file mep_2374-8265.11085-s001.zip › _Educational_Summary_Report_11085.pdf]

MedEdPORTAL® The AAMC Journal of Teaching and Learning Resources

Original Publication Open Access

A Novel Team-Based Learning Approach for an Internal Medicine Residency: Medication-Assisted Treatments for Substance Use Disorders

Daniel Matassa, MD*, Benjamin Perrella, MD, Mirela Feurdean, MD

*Corresponding author: matassdm@njms.rutgers.edu

Abstract

Introduction: It is estimated that approximately one-tenth of the US population suffers from substance use disorders (SUD), a problem that is compounded when one considers the impact that drug addiction could have on treatment outcomes for many other chronic diseases. Thus, addiction medicine has become an important component of many successful urban primary care practices and residencies across the country. Our program sought to improve the confidence of our residents in managing SUD by instituting a team-based learning (TBL) activity that focused on the diagnosis and medication-assisted treatment of these illnesses. **Methods:** The class of 80 internal medicine residents were divided into groups of approximately 16 residents, and during the TBL sessions further divided into teams of three to four. Each TBL session consisted of an individual readiness assurance test, a group discussion of the correct answers, and a PowerPoint-based team application activity. Surveys were conducted for each group to assess the residents' attitudes after completing the activity. **Results:** Of residents, 69 of 80 completed the survey. The response to the TBL exercise was overwhelmingly positive, with most residents in agreement that the activity increased their knowledge and confidence in diagnosing and treating patients with SUD. **Discussion:** Overall, this TBL activity was well received by the residents and subjectively increased their competence in managing patients with SUD. In addition, our modification to the traditional TBL format suggested that the theories and spirit behind TBL can be successfully adapted to meet the challenges and intricacies of internal medicine residency education.

Keywords
Team-Based Learning, TBL, Substance Use Disorder, Medication Assisted Treatment, Addiction, Internal Medicine

Educational Objectives

By then end of this activity, learners will be able to:

1. Conduct an appropriately detailed substance use history.
2. Identify the signs of intoxication and withdrawal from alcohol, marijuana, cocaine, and opioids.
3. Define the criteria for a substance use disorder and make an appropriate diagnosis.
4. Select appropriate candidates for medication-assisted treatment for opioid use disorder.
5. Recommend treatment options in the management of alcohol use disorder.

Citation:
Matassa D, Perrella B, Feurdean M. A novel team-based learning approach for an internal medicine residency: medication-assisted treatments for substance use disorders. *MedEdPORTAL*. 2021;17:11085. https://doi.org/10.15766/mep_2374-8265.11085

Introduction

Approximately 10% of the US population struggles with drug addiction,[1] and physicians in all medical and surgical specialties are bound to meet these patients on a daily basis. Addressing addictions appropriately is paramount to improved patient outcomes on many different levels, from chronic disease management to psychological health. As such, all internists, especially those who practice as front-line primary care providers, should be trained in recognizing and initiating appropriate treatment for addiction.[2,3]

Managing patients with addictions can often be challenging and most internal medicine residents feel unprepared to treat substance use disorders (SUD).[4] Integration of SUD education in residency curricula varies widely across the country and by specialty, with the most common obstacle cited being lack of time. New strategies that integrate into existing residency structures are needed to improve SUD training.[5]

We proposed a modified team-based learning (TBL) activity to enhance patient care, resident awareness, and education in SUD

Copyright © 2021 Matassa et al. This is an open-access publication distributed under the terms of the Creative Commons Attribution license.

diagnosis and treatment, with a focus on medication-assisted treatment of opioid and alcohol use disorders. To our knowledge, a similar module has not been published. The development of this TBL module was based upon motivational, reflective, and scaffolding learning theories and followed published guidelines with regard to structure[6,7] and reporting outcomes.[8] In addition, our PowerPoint-based TBL exercise integrated research articles and evidence-based guidelines to facilitate discussion and critical thinking. This activity was incorporated into the established structure of our ambulatory care curriculum.[9]

Methods

Team Formation

Our residency program follows a block schedule, with five groups of residents rotating through ambulatory care in 2-week increments. During ambulatory blocks, Friday mornings are dedicated to ambulatory half days, which include TBL exercises and other workshops. At the time that this module was developed, each ambulatory group was composed of an average of 16 residents. We divided the group into five teams of three to four residents, with all PGY levels represented. Teams were organized by the facilitator and changed with every TBL exercise.

Advance Preparation

One week prior to our in-class TBL activities, we required residents to review two online modules from Johns Hopkins' Physician Education and Assessment Center, Addiction: Illicit Drugs and Alcoholism and Unhealthy Alcohol Use,[10] and completion of this task was monitored. Use of this preparatory resource insured that the residents possessed similar fundamental knowledge in order to participate in the case-based scenarios of our TBL exercise.

Readiness Assurance Process

The individual readiness assurance test (iRAT; Appendices A and B), composed of several open-ended and fill-in-the-blank style questions, was delivered in paper format immediately prior to the TBL exercise. Residents were not permitted to use books, articles, or mobile devices during the iRAT. The iRAT was employed purely as a means to assess compliance with, and establish the baseline knowledge of, the advance preparation modules. Because of its very limited impact on a resident's overall performance evaluation, we did not feel that a formal grading of the iRAT would be a justified use of the facilitator's time. Therefore, instead of iRAT grading, our next step was to review the quiz as a group. No specific, individualized feedback was provided to the residents.

The facilitator worked through the iRAT and asked the group, as a whole, for an answer to each question. Follow-up questions were often asked based on the residents' responses and inquiries. During this review, the facilitator encouraged involvement from everyone in the group discussion, indirectly assessed each participant's understanding of the advance preparation resources, and insured group readiness for the application activity. After completion of the iRAT group review, which took the place of a traditional gRAT, the group was broken up into teams for the case-based application exercise.

Team Application Exercise

The team application exercise PowerPoint (Appendix C) met the 4S criteria (i.e., significant problem, same problem, specific choice, simultaneous reporting) of a typical TBL: teams worked on the same, significant problem, and selected specific choices simultaneously.[6] The exercise included six different clinical vignettes. There were eight multiple-choice options for each question, and teams were instructed that there could be more than one correct answer for each one. Allowing different combinations of acceptable options helped to create a vigorous debate and enhance critical thinking amongst the teams, as well as demonstrate that there can be more than one solution to a clinical problem. Teams moved through one vignette at the same time. Approximately 5 minutes were allowed for them to debate the answers within their teams, after which they revealed their choice(s) simultaneously by raising colored cards with the letter(s) of their selection(s). The facilitator then probed the teams to highlight important clues within the vignette, in order to illustrate salient learning points. The teams defended their choices, and the facilitator helped guide discussions when different opinions emerged. The correct answer(s) were subsequently revealed, points for correct answers were tallied, and a few educational slides followed, which detailed relevant literature and learning aids to support the vignette's conclusions. The group continued to the next vignette, and the same process was followed until completion of the PowerPoint.

To aid the facilitator, we have included a separate file with detailed explanations for each question in the PowerPoint (Appendix D).

Facilitation Schema

The total time was 2 hours, broken up as the following:

- iRAT individual completion, 12 minutes.
- iRAT group discussion of correct answers, 15 minutes.
- Formation of TBL teams, 3 minutes.
- Team application exercise, 1.5 hours.

Copyright © 2021 Matassa et al. This is an open-access publication distributed under the terms of the Creative Commons Attribution license.

During the team application exercise roughly 15 minutes were allotted to each question: 5 minutes were spent allowing teams to debate the answer choices amongst themselves, 5-7 minutes were spent discussing the vignette and correct answers as a group, and the remaining 3-5 minutes were devoted to a review of the interactive and educational supporting slides that followed.

Evaluation
Within 4 weeks of attending the TBL activity, a survey was electronically delivered to all participating residents (Appendix E). This evaluation, created by the primary TBL moderator, used a 5-point Likert scale (1 = *strongly disagree*, 5 = *strongly agree*) to gauge resident attitudes about their management of SUD, specifically after attending the TBL exercise. Specifically, our survey sought to measure residents' perceived completion of the educational objectives and asked them to self-assess their competence in managing patients with SUDs. The survey also included one optional open-ended question seeking constructive feedback about the activity.

Results

Of the 80 categorical internal medicine residents that participated in the TBL, there were 27 PGY 1, 26 PGY 2, and 27 PGY 3 trainees. Of residents, 69 of 80 (86%) responded to the survey. The Table summarized the responses from the residents, specifically seeking to understand how the TBL impacted their perceived level of confidence, knowledge, and understanding of this topic. Negative responses included selections of *disagree* and *strongly disagree*. Positive responses included selections of *agree* and *strongly agree*. Seven of the nine questions had a positive response rate of greater than 91%. The other two questions on residents' postTBL confidence in offering medication-assisted treatments for their patients with opioid or alcohol use disorders (as opposed to referring to an addiction specialist) had an 81% positive response with neutral responses near 17%.

The evaluation also included an open-ended question, seeking constructive feedback for the TBL exercise. Of responses, 16 of 22 offered positive reflection on the activity; one learner described the TBL activity as a "structured, very informative learning session," while another stated that the "cases and questions were stimulating and helped reinforce the material." Five comments offered minor suggestions for improvement, which mostly included "focusing more on motivational interviewing in future sessions." One comment sought to use the material to improve practice in our clinic by providing "pocket cards of available treatment options for SUDs to our residents and nurses."

Discussion

Our program is located in an urban area with a high prevalence of patients with SUDs and limited resources for adequate treatment. Other health systems throughout the US are undoubtedly facing some of the same issues during the current national opioid epidemic. As mentioned above, population data suggest that around 10% of the US population suffers from SUDs.[1] Hence, training and experience in diagnosis and treatment of addictions is essential to our internal medicine residency graduates.

We developed and implemented this modified TBL model within our program's ambulatory curriculum, and published survey data has demonstrated an overall resident satisfaction score $>$ 95% with our TBL-centered curriculum.[9] In our experience, TBL activities offer a structured but flexible framework that is engaging and fun for both faculty and trainees, and which can be successfully adapted to the realities and cultures of various types of residency programs. By publishing successful content within

Table. Resident Responses to TBL Evaluation (*N* = 69)

Question	Strongly Disagree or Disagree (%)	Neutral (%)	Strongly Agree or Agree (%)
The TBL increased knowledge and understanding of DSM-5 criteria for substance use.	2	2	96
The TBL increased knowledge and understanding of the negative and dangerous effects of various substances of abuse (e.g., cocaine, opioids, alcohol, cannabis).	2	3	95
The TBL increased my knowledge and understanding of the various options available for the treatment of opioid use disorder.	0	2	98
As a result of this TBL, I feel more comfortable diagnosing and counseling patients with substance use disorders.	4	5	91
As a result of this TBL, I am more likely to suggest prescribing buprenorphine or referring to a methadone clinic, rather than reflexively sending the patient to an addiction specialist.	2	17	81
As a result of this TBL, I am more likely to suggest medication-assisted therapies for my patients with alcohol use disorder.	4	16	80
I feel more competent in treating patients that suffer from addiction.	3	5	92
The information in the TBL was relevant to my practice in primary care.	2	7	92
Compared to attending-based lectures in similar topics, I found the TBL to be more informative and engaging.	2	5	93

Abbreviations: DSM-5, Diagnostic and Statistical Manual of Mental Disorders 5th edition; TBL, team-based learning.

Copyright © 2021 Matassa et al. This is an open-access publication distributed under the terms of the Creative Commons Attribution license.

this pedagogical framework, we hoped to help other residency educators to implement TBL activities into their curricula, while adapting the workflow to their programs' needs and constraints.

There is a paucity of published literature on the use of TBL in internal medicine residency education, and even less when the focus is narrowed to topics in addiction medicine. We believe that our work has been valuable in two distinct ways: it has contributed to a very small repository of resources in teaching addiction medicine to residents, and it has also detailed the workflow of a modified TBL pedagogy that may be more efficient to administer to residents than traditional formats.

Our modified approach remained grounded in the educational theories that support traditional TBL, including the seven core design elements and the 4S of application.[6-8] However, our approach departed at a few points from the traditional and rigorously evidence-based TBL structure.[6] To start, our teams were constructed differently than has been traditionally reported in the literature. First, they were not established to be permanent units, but rather were randomly selected with each session. This was necessary for us, mainly because our residents often have complicated schedules, and establishing permanent groups would not have been feasible. Secondly, we also chose to make group sizes of only three to four residents, as opposed to the traditional five to seven.[6-7] Because learners from three PGY levels were included in each group, we had found during prior TBL activities that the voices of the more inexperienced trainees were often overshadowed within larger teams. Smaller teams have consistently led to more spirited and inclusive team debates over our 5 years of delivering a TBL-based curriculum. Adapting within changing teams is also a skill expected of practicing physicians, which we also hoped to foster in this design.

In addition to modifying our team structure, our exercise also deviated from customary TBL in its readiness assurance materials. In traditional TBL format, the iRAT is completed and followed by a group readiness assurance test (gRAT) using the same questions. These activities, which generally impact a learner's score or grade, ensured that a baseline knowledge level was met, promoted accountability for the preclass preparation materials, and helped the teams to develop cohesion prior to the application exercise.[6-7] In our model, we essentially condensed the iRAT and gRAT into one activity. Residents completed the iRAT, and the answers were then reviewed as a group, with every learner expected and encouraged to contribute. Although this process was different than classic TBL and not as robust, it still accomplished the aforementioned goals and set the stage for further discussion moving into the application exercise. Formal grading for these activities was not necessary for us, as it would not have been useful within our current residency evaluation framework.

Lastly, our exercise did not include a formal appeals process typical for classic TBL. We instead allowed appeals informally throughout the application exercise, which we then used to elevate the discussion that took place. Learners were encouraged to research ambiguities in real time and contribute those findings to the class.

It is important to note that most TBL literature in medical education has been focused on medical school curricula. Residency education possesses innate differences that impact the implementation of curricular objectives, especially with consideration of its structure, evaluative process, and often its time constraints. Therefore, we felt that these TBL modifications were necessary to maximize the impact of this TBL activity on our residents' education. Nevertheless, adaptation of this exercise can certainly be made to fit the needs of another program or learner type. For example, implementation of a classic iRAT, gRAT, and appeals process can be done using our framework, specifically for learner groups in which greater accountability is needed or desired. TBL is an evolving pedagogy in graduate medical education, and active scholarship detailing varieties of implementation, according to published guidelines, is necessary to grow a body of evidence in support of a gold standard.[8]

The survey results showed that our residents appreciated our modified TBL approach and its content. The addiction medicine TBL activity subjectively enhanced their knowledge in general SUDs and medication-assisted treatments and helped them to feel more competent in managing addictions. Of residents, 80% agreed that the TBL increased their likelihood of suggesting medication-assisted treatments for opioid and alcohol use disorders, rather than reflexively referring to an addiction specialist. It is unclear to us whether the 16% of neutral responders remained uncomfortable with suggesting treatment options themselves, or if they already felt comfortable doing so and the TBL had no impact on this; reasons for this response were likely varied. Compared to a traditional lecture, all residents found TBL to be more engaging and informative. The comments were universally positive, most residents remarking about the enjoyable and educational nature of the activity. Suggestions for further content improvement included motivational interviewing techniques and pocket cards with addiction treatment resources. Our results supported and highlighted the need to include addiction medicine education in internal medicine residency curricula.

Copyright © 2021 Matassa et al. This is an open-access publication distributed under the terms of the Creative Commons Attribution license.

We did recognize a number of limitations in our work. First of all, as discussed above, our workflow departed at several points from traditional TBL formats. While we felt that these adjustments were necessary for our program and did not detract from the educational theory behind TBL, our specific modifications have never been directly studied or validated. Secondly, our only mode of evaluation was a survey looking at residents' perceived knowledge of and attitudes towards addiction treatment. The impact of our addiction TBL activity on patient care outcomes, which would have clearly strengthened the value of our work, would have also been too difficult for us to assess and separate from other educational experiences (e.g., participation in buprenorphine prescribing in resident clinics, exposure to SUD treatment for inpatients, etc.). Thirdly, we recognized that our approach may not be generalizable to every program. Every institution has different variables with regard to residency program size, protected time for resident education, patient demographics, and faculty expertise and availability. Therefore, while our approach was adapted to fit our needs and was well received in our program, it may need to be further modified to fit others' specific situations.

The success we have had in administering this module to our residents has led to a number of changes in our ambulatory curriculum. As one example, we have used residents' feedback to improve other team-based modules we have developed in various ambulatory topics. In addition, we have also harnessed the residents' strong desire for more training in addiction medicine, translating this into more robust addiction services for our patients and more in-depth rotation experiences for our trainees. As we move into the future, we look to further expand our curriculum in addiction medicine and our commitment to team-based educational methods. We hope that other programs can benefit from our work and use it to expand their own addiction and TBL curricula.

Appendices

A. iRAT without Answers.docx

B. iRAT with Answers.docx

C. Team Application Exercise.pptx

D. Facilitators Guide to the Team App Exercise.docx

E. Resident Evaluation of the TBL Activity.docx

All appendices are peer reviewed as integral parts of the Original Publication.

Daniel Matassa, MD: Assistant Professor, Department of Medicine, Rutgers New Jersey Medical School; ORCID: https://orcid.org/0000-0001-7390-7007

Benjamin Perrella, MD: Resident, Department of Medicine, Rutgers New Jersey Medical School

Mirela Feurdean, MD: Associate Professor, Department of Medicine, Rutgers New Jersey Medical School; ORCID: https://orcid.org/0000-0003-2620-3837

Disclosures
None to report.

Funding/Support
None to report.

Ethical Approval
Reported as not applicable.

References

1. *National Survey on Drug Use and Health (NSDUH)*. Substance Abuse and Mental Health Services Administration. 2019. Accessed October 6, 2020. https://www.samhsa.gov/data/data-we-collect/nsduh-national-survey-drug-use-and-health
2. Weimer MB, Tetrault JM, Fiellin DA. Patients with opioid use disorder deserve trained providers. *Ann Intern Med*. 2019;171(12):931-932. https://www.doi.org/10.7326/M19-2303
3. O'Connor PG, Nyquist JG, McLellan AT. Integrating addiction medicine into graduate medical education in primary care: the time has come. *Ann Intern Med*. 2011;154(1):56-59. https://www.doi.org/10.7326/0003-4819-154-1-201101040-00008
4. Wakeman SE, Baggett MV, Pham-Kanter G, Campbell EG. Internal medicine residents' training in substance use disorders: a survey of the quality of instruction and residents' self-perceived preparedness to diagnose and treat addiction. *Subst Abus*. 2013;34(4):363-370. https://www.doi.org/10.1080/08897077.2013.797540
5. Isaacson JH, Fleming M, Kraus M, Kahn R, Mundt M. A national survey of training in substance use disorders in residency programs. *J Stud Alcohol*. 2000;61(6):912-915. https://doi.org/10.15288/jsa.2000.61.912
6. Parmelee D, Michaelsen LK, Cook S, Hudes PD. Team-based learning: a practical guide: AMEE guide no. 65. *Med Teach*. 2012;34(5):e275-e287. https://doi.org/10.3109/0142159x.2012.651179
7. Burgess AW, Mcgregor DM, Mellis CM. Applying established guidelines to team-based learning programs in medical schools.

Copyright © 2021 Matassa et al. This is an open-access publication distributed under the terms of the Creative Commons Attribution license.

Acad Med. 2014;89(4):678-688. https://www.doi.org/10.1097/ACM.0000000000000162

8. Haidet P, Levine RE, Parmelee DX, et al. Perspective: guidelines for reporting team-based learning activities in the medical and health sciences education literature. *Acad Med*. 2012;87(3): 292-299. https://doi.org/10.1097/acm.0b013e318244759e
9. Feurdean M, Matassa D, Kang M, Matthews G, Kothari N. A modified team-based learning approach to enhance resident education in outpatient internal medicine. *MedEdPublish*. 2017;6(4). https://www.doi.org/10.15694/mep.2017.000176
10. Physician Education and Assessment Center (PEAC). Accessed May 15, 2020. https://peaconline.org/

Received: May 19, 2020
Accepted: October 19, 2020
Published: February 1, 2021

Copyright © 2021 Matassa et al. This is an open-access publication distributed under the terms of the Creative Commons Attribution license.
